# Exploring the induction and measurement of positive affective state in equines through a personality-centred lens

**DOI:** 10.1038/s41598-025-98034-8

**Published:** 2025-05-27

**Authors:** Loni Loftus, Amy Newman, Matthew Leach, Lucy Asher

**Affiliations:** 1 School of Natural and Environmental Sciences, Newcastle University, Newcastle upon Tyne, NE1 7RU UK; 2 Royal (Dick) School of Veterinary Studies and the Roslin Institute, Easter Bush Campus, University of Edinburgh, Midlothian, EH25 9RG UK; 3 University Centre Askham Bryan, Askham Bryan, York, YO23 3FR UK; 4 Comparative Biology Centre, Medical School, Newcastle University, Framlington Place, Newcastle upon Tyne, NE2 4HH UK

**Keywords:** Equine, Emotion, Positive affect, Behavioural measures, Physiological measures, Personality, Animal behaviour, Personality

## Abstract

There is increasing focus on how to induce and measure positive affective states in animals and the development of social license to operate has brought this to the forefront within equestrianism. This study aimed to utilise a range of methods to induce and measure positive affect in horses in real-world settings. Twenty healthy horses were scored for personality, exposed to four induction methods (wither scratching, high value food provision, positive reinforcement training and the addition of an affiliative conspecific), and data collected on their behaviour (QBA and ethograms) and physiology (heart and respiratory rate, heart rate variability, eye and ear thermography and salivary cortisol). Analyses identified potentially sensitive and specific behavioural (ear and eye position, QBA items, frustration items) and physiological (RR mean, HF power, LF power, LF/HF ratio, mean HR, RMSSD and pNN50) measures of affective state across the four quadrants of core affect. Individual difference effects were found, and personality traits such as unfriendly, nervous and unresponsive were associated with differing responses to induction stimuli indicating that all four induction stimuli are potentially useful for inducing positive affect depending on their salience to the individual. Research measuring and inducing positive affect in animals rarely considers personality, but this study underscores its importance. The dimensional approach taken allowed for assessment of the broad arousal and valence components of affect without ascribing measures to discrete emotions. Accurate, real-world measures of affect could benefit 116 million equines globally, and exploring ways to promote positive affect in horses can significantly enhance their welfare.

## Introduction

Emotions (short-term valenced states)^1^ are notoriously difficult to study in animals since they are not able to self-report. However, over the last twenty years research has progressed our understanding of how to assess animal emotion^2,3^. ‘*Affect*’ is an overarching term for states (affective states) that have valence (they are positive or negative). Affect includes short-term emotions and longer-term moods but also the valenced components of sensations and non-conscious components such as behavioural indicators and neural changes^1^. Core affect refers to the change in the internal environment in two key dimensions; emotional valence (positive and negative) and arousal (a state of physiological alertness and readiness for action)^1,4^. A rise in welfare standards, coupled with the requirement to consider both physiological and psychological welfare within welfare assessment, has led to a renewed focus on emotions in animals and positive welfare states including positive affective states^5^. The development of Social Licence to Operate (SLO) (societal approval or acceptance of the activity) in the equestrian industry further motivates research focussed on promoting positive affect in domesticated equines to enhance welfare^6,7^.

Behavioural and physiological measures are used as proxies for identification of affective state in animals. These outputs can be considered in terms of opportunities for pleasure (positive affect) or avoidance of threats (negative affect)^8^. From a behavioural perspective, affiliative behaviour, play and information gathering activities have been proposed as potential positive indicators^9^. These are behaviours said to be mediated by the seeking, care, and play systems^10^ and therefore unlikely to be performed by animals in negative affect^1,2,11,12^. Some of these are included in behavioural measures of the AWIN project^13^ which developed a welfare assessment protocol for horses which included: social interaction; stereotypy performance (or absence of); fear (or absence of); human-animal relationships assessed via avoidance distance, voluntary animal approach and forced human approach tests; and qualitative behaviour assessment (QBA). QBA was included in the 2004–2009 European Commission’s Welfare Quality Audit and identified as the only measure which captures positive welfare^14^ however, some authors have reported lower levels of reliability when using QBA as a standalone welfare assessment tool^15^. Hall and Heleski^16^ suggest that ethograms of behaviour are important means of evaluating both the affective and physical welfare of horses alongside physiological measures. Whilst overt body language is important in understanding horse welfare, more subtle signs may be displayed by equines either prior to, or in place of, more overt body language^17^; each can be evaluated via ethograms. Facial actions can be reliably recorded using the Equine Facial Action Coding System, EquiFACS^18^, which has previously been utilised in attempts to study positive affective state^19–21^. Subtle indicators might also include lateralisation of behaviour, which has been more widely studied in other species (reviewed by Rogers et al.^22^) and recently in horses^23,24^. One further aspect of behaviour that is relevant to the current study is frustration, defined as the thwarting of impulses or actions that prevents individuals from obtaining something they expect based on past experience^25,26^. In studies presenting affective stimuli there is potential for transient alteration in affective state, for example where initial positive affect may switch to negative affective states, such as frustration, if the goal is not achieved^27^. Frustration may be evaluated using previously identified behavioural signs, including repetitive behaviour^28^.

Physiological measures have also been used as indicators of affective state in horses. Heart rate and heart rate variability (HRV, using time-, frequency- and non-linear-domains) have been widely used in the assessment of stress in horses^29–32^. Whilst heart rate may indicate arousal (an important component of core affect), there have also been efforts to decompose the heart rate signal into a physical and an emotional component^33^. Visser et al.^34^ found high heart variability (HRV) was associated with exploratory behaviour, but not freezing behaviour, whereas heart rate was associated with anxious behaviour. Another candidate physiological indicator of positive affect is cortisol. Just as increases in cortisol are likely reflective of acute stress-like states, decreases in cortisol may indicate a transition to more positive, lower arousal, affective states^35^. However studies have found lower levels of faecal cortisol metabolites and plasma cortisol in equines with chronically compromised welfare^36^ and increases in cortisol levels can be associated with arousal^37^, suggesting that this measure should not be utilised in isolation to measure affective state. Peeters et al.^38^ proposed using salivary cortisol as a refinement to serum, given a high correlation between the two when exposed to an adrenocorticotropic (ACTH) challenge. In horses lower respiratory rate has been associated with positive affect for example, Briefer et al.^39^ identified a lower respiratory rate in horses listening to affiliative reunion vocalisations. Supporting this Menchetti et al.^40^ identified lower respiratory rates in horses with higher scores for calmness. Finally, Waran and Randle^41^ proposed that eye temperature may be a suitable metric for assessing affective state. Ramirez Montes De Oca^42^ identified changes in the lateralisation of nostril, eye and ear temperatures in dairy calves in rewarded and non-rewarded trials with increasing and decreasing temperatures identified at different anatomical sites, a finding also supported by Kappel^43^ in horses. However, evidence in horses is currently limited and requires further validation.

Whilst there are promising candidate measures of positive affective state, research is still at an early stage compared to identification of negative states^44^. To be able to measure affective state it must be reliably induced. One of the challenges of research on affective states is therefore to identify species-specific stimuli that are likely to induce short- or longer-term changes in affective state that are consistent in valence. The ethology of the species combined with prior experience, current environment and internal state will influence whether a particular stimulus induces a positive or negative state. In horses, possibly the best validated induction method is scratching the withers which appears to induce a low arousal positive affect^19,20,45,46^. A further complication is the individual’s personality. Strong evidence from the human literature^47–50^ and developing evidence from the animal literature^51–56^, suggests that personality should be considered within studies of animal affect and this was supported by our prior Delphi consultation^57^.

High value food provision is ethologically relevant to equines^58^ but has the potential to induce alternative affective responses. For example, it is recognised that high value food provision may induce higher arousal in some equines^59^, which may lead to anticipatory behaviours^60^ and frustration-related responses^28^ rather than a low arousal, positive affective state. In addition, the relative value of perceived high value feedstuff within individuals, according to their personality and prior experiences, is deemed likely to result in a range of affective experiences to induction using this method^61^. The addition of a known affiliative companion has been demonstrated to enhance equine welfare through providing for ethological needs^62–64^, potentially inducing positive affect in low arousal. However, understanding the nature of the relationship between the two parties is a crucial factor to ensure that an appropriate selection of social partner is undertaken to induce positive rather than negative affect, and the emotional state of the added conspecific must be considered to avoid instances of negative emotional contagion^65^. Prior research has suggested that positive reinforcement training may enhance short- and longer-term positive affective state^66–68^ and increase motivation^69^ but could conversely lead to frustration if reinforcement is poorly timed or withheld^28^. Wither scratching is supported within the literature as a potential measure of positive affect induction^19,20,70,71^, imitating the relationship building and reassurance seen in allogrooming^58,72^. However, individual preferences have been identified relating to scratching site location^19,45,73^ and further individual variation should be considered relating to personality and prior experience when identifying whether an equine is expected to positively respond to human touch^46,74,75^.

The aim of this study was to utilise a range of expert suggested methods (wither scratching, high-value food provision, positive reinforcement target training and the addition of an affiliative conspecific)^57^ to attempt to induce positive affect in a mixed cohort of equines and to use expert suggested behavioural and physiological measures^57^ to evaluate the actual affective state induced in a real-world setting. The study also assessed and explored the effects of equine personality on the type of affective state induced^76^. Firstly, it was hypothesised that the presentation and subsequent removal of the four induction stimuli would induce differing levels of arousal, and types of valence (positive or negative), within individual horses. We also hypothesised that these affective states (differences in types of valence and levels of arousal) would be evidenced through differences in the behavioural and physiological responses recorded between induction stimuli, and during the phases of the trial (during and after presentation of the stimulus). Thirdly, we hypothesised that the affective states produced via each of the four induction methods may, in part, be affected by individual personality traits. It was also hypothesised that there may be identifiable groupings of measures that may indicate differing levels of arousal and types of valence across the subject cohort.

## Results

### Reliability of measures

All measures which were recorded by human observers showed good to excellent rater agreement (Respiratory rate intra-rater reliability: ICC, two-way mixed effects, single measures, absolute agreement = 0.995, *p* < 0.001; other behaviour measures inter-rater reliability between 5 raters, ICC, one-way random effects, average measures, absolute agreement = 0.793–0.988, all *p* < 0.001).

### Data reduction

From an initial 65 measures, 27 potential measures of affective state were identified to be taken forward (Table 1), based on (1) removal of variables which were highly correlated with other variables; (2) correlation with highly validated QBA measures; (3) support from literature to select between highly correlated measures; (4) ensuring a range of measures across core affect dimensions to distinguish between negatively and positively valenced states and high and low arousal. Measures with low n occurrence were removed. Correlations can be viewed in supplementary information S1 and further statistical and literature-based rationale for measure inclusion can be found in supplementary material S2.

**Table 1:** Potential measures of affective state selected after data reduction procedures, rationale for inclusion and other correlated measures.

Measure	Inclusion rationale				Positively correlated variables (≥ 0.5)	Negatively correlated variables (≥ − 0.5)
	Correlation with QBA measures	Literature support	Valence	Arousal		
Ears forward*	0.05	Yes^77^	+	$\wedge / \vee$	None	None
Ears sideways*	− 0.01	Yes^77–79^	+	$\vee$	None	None
Ears asymmetrical*	− 0.08	Yes^20,78^	−	$\wedge$	Ears gently back	None
Ears gently back*	0.15	Yes^78,79^	+	$\vee$	Ears asymmetrical	None
Ears fully back*	− 0.56	Yes^77^	−	$\wedge$	Frustration acute conflict	None
Eyes half open*	0.04	Yes^20,80^	+	$\vee$	None	None
Eye sclera*	− 0.44	Yes^20,79^	−	$\wedge$	None	None
Eye bias both*	− 0.20	Yes^81^	+/−	$\wedge / \vee$	Eye bias right, Eye bias left	None
Eye bias left*	− 0.18	Yes^79,82,83^	+/−	$\wedge / \vee$	Eye bias both	None
Eye bias right*	− 0.25	Yes^81^	+/−	$\wedge / \vee$	Eye bias both	None
Frustration acute conflict	− 0.43	Yes^28^	−	$\wedge$	Ears fully back	None
Frustration increased locomotion	0.05	Yes^28^	−	$\wedge$	None	None
Mean heart rate (BPM)*	0.19	Yes^84^	+/−	$\wedge / \vee$	None	RR mean
LF power*	− 0.02	Yes^85^	+/−	$\wedge$	None	HF power
HF power*	− 0.03	Yes^85^	+/−	$\vee$	None	LF power
LF:HF ratio*	− 0.22	Yes^85^	+/−	$\wedge / \vee$	None	None
RR mean*	− 0.08	Yes^85^	+/−	$\wedge / \vee$	None	None
pNN50*	0.06	Yes^85^	+/−	$\wedge / \vee$	RMSSD	None
RMSSD*	0.01	Yes^85^	+/−	$\wedge / \vee$	SDRR, pNN50	None
SDRR*	− 0.01	Yes^85^	+/−	$\wedge / \vee$	RMSSD	None
Salivary cortisol*	− 0.03	Yes^86–91^	+/−	$\wedge / \vee$	None	None
Respiratory rate*	0.01	Yes^39,40,92–94^	+/−	$\wedge / \vee$	None	None
QBA Friendly	N/A	Yes^13,95–97^	+	$\wedge / \vee$	None	None
QBA Relaxed	N/A	Yes^13,95–97^	+	$\vee$	None	None
QBA At ease	N/A	Yes^13,95–97^	+	$\vee$	None	None
QBA Alarmed	N/A	Yes^13,95–97^	−	$\wedge$	None	None
QBA Uneasy	N/A	Yes^13,95–97^	−	$\wedge$	None	None

Indication as to the proposed different dimensions of core affect are represented by positive (+) or negative valence (−) and high ($\wedge$) or low arousal ($\vee$). * Denotes inclusion within later Linear Discriminant Analysis.

### Linear discriminant analysis (LDA)

**Fig. 1:**
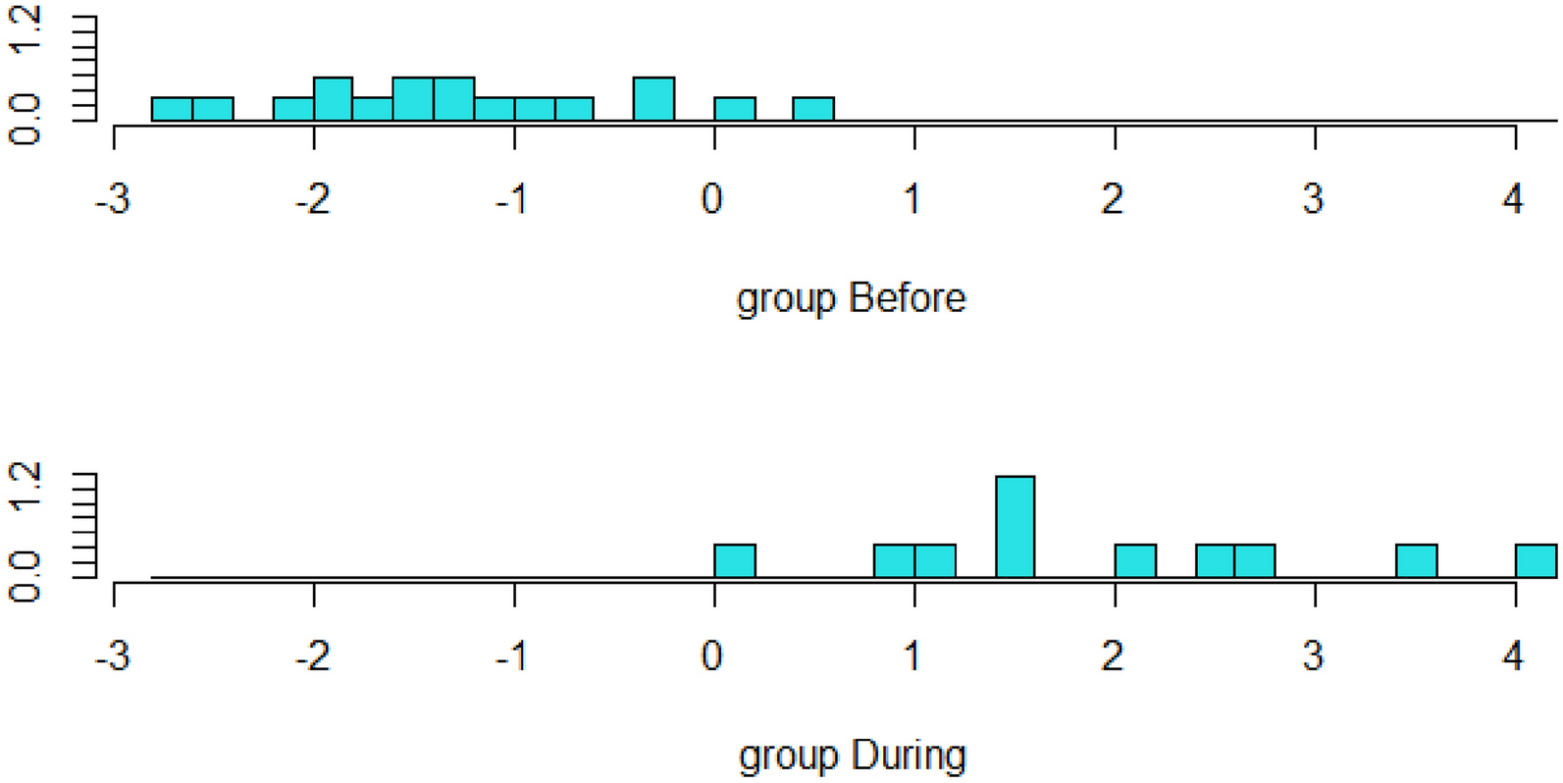
Separation of variables between the ‘before’ and ‘during’ phases of the scratch trial. With correlations of greater than 0.7 identified between variables ‘LF power’ and ‘LF:HF ratio’.

Using wither scratch as the best validated induction method^19,20,46,70,71^ to identity potential measures of positive affective state, a linear discriminant analysis was able to distinguish before and during phases (Fig. 1). The variables included in this LDA (denoted by * in Table 1) were taken forward to Principal Component Analysis (PCA), alongside the other measures listed in Table 1.

### Principal component analysis (PCA)

**Fig. 2:**
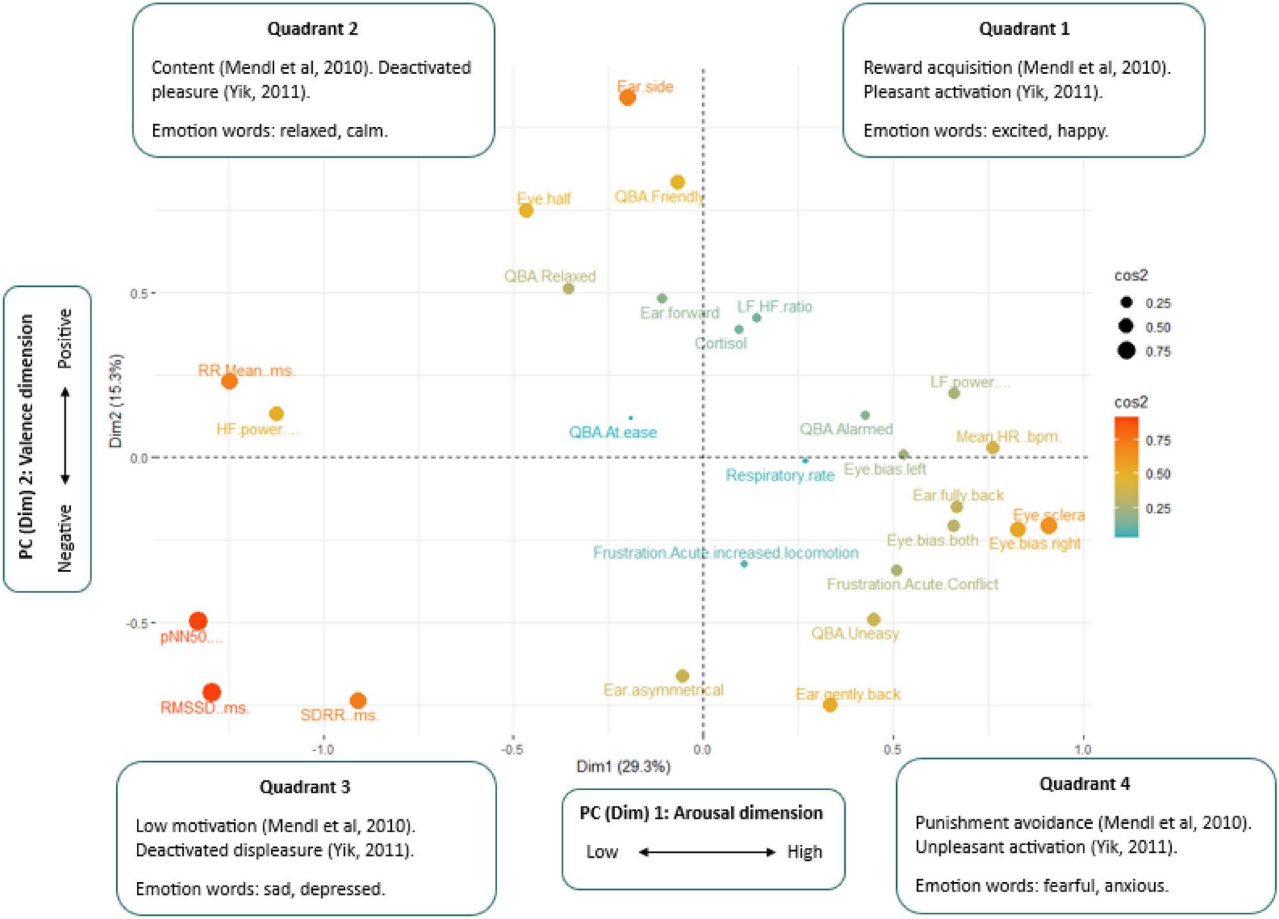
Proposed model for measures of valence and affect in equines based upon the framework of Mendl et al.^2^ following evaluation of measurement variable relationships. Dimension 1 is proposed as the arousal dimension and dimension 2 as the valence dimension. Quadrant 1 is proposed as a higher arousal, positively valenced domain which may be evaluated via assessment of several HR/HRV parameters (Low -Frequency power, LF-HF ratio and Mean heart rate) as well as cortisol levels and potentially one QBA descriptor. Quadrant 2 is proposed as a lower arousal, positively valenced dimension which may be evaluated via assessment of different HRV parameters (RR Mean and High-Frequency power) as well as several QBA descriptors indicative of relaxation and positive valence. Quadrant 3 is proposed as a negatively valenced, lower arousal domain evaluated via assessment of further discrete HRV parameters (pNN50, RMSSD and SDRR) as well as one QBA descriptor. Lastly, quadrant 4 is proposed as a higher arousal, negatively valenced domain evaluated via a number of discrete QBA (Uneasy), behavioural (Frustration, eye bias, eye position and ear position) and physiological (respiratory rate) parameters.

In order to consider the hypothesis that there may be identifiable groupings of measures that may indicate differing levels of arousal and types of valence across the subject cohort PCA analysis was applied to all induction methods across all trials (before, during and after) and identified that the first five components accounted for 71% of the variation within the data (PC1: 29%, PC2: 16%, PC3: 13%, PC4: 8%, PC5: 5%). The PCA loading plot was considered in reference to the available literature and using relationships between the selected measures to infer the association with varying valenced states (positive and negative) and levels of arousal (low and high) (Fig. 2). Principal component (PC) 1 (Dim1) appears to be related to the arousal dimension with grouping of measures indicative of high arousal such as ‘low-frequency power’, ‘mean heart rate’, ‘cortisol’, ‘ears fully back’, ‘frustration’ and negative ‘QBA’ measures loading positively on PC1 and groupings of measures indicative of low arousal including ‘high-frequency power’, ‘RR mean’, ‘ears forward’ and positive ‘QBA’ measures, conversely loading negatively on PC1. PC2 is suggested as a valence dimension with grouping of indicators of potentially negative valence such as ‘ears fully back’, ‘respiratory rate’, ‘ears asymmetrical’, ‘eye sclera’, negative ‘QBA’ measures and ‘frustration’ responses loading negatively and indictors of grouping of measures potentially indicating positive valence such as ‘ears forward’, ‘ears side’, positive ‘QBA’ measures and ‘eyes half’ loading positively (Fig. 2).

**Fig. 3:**
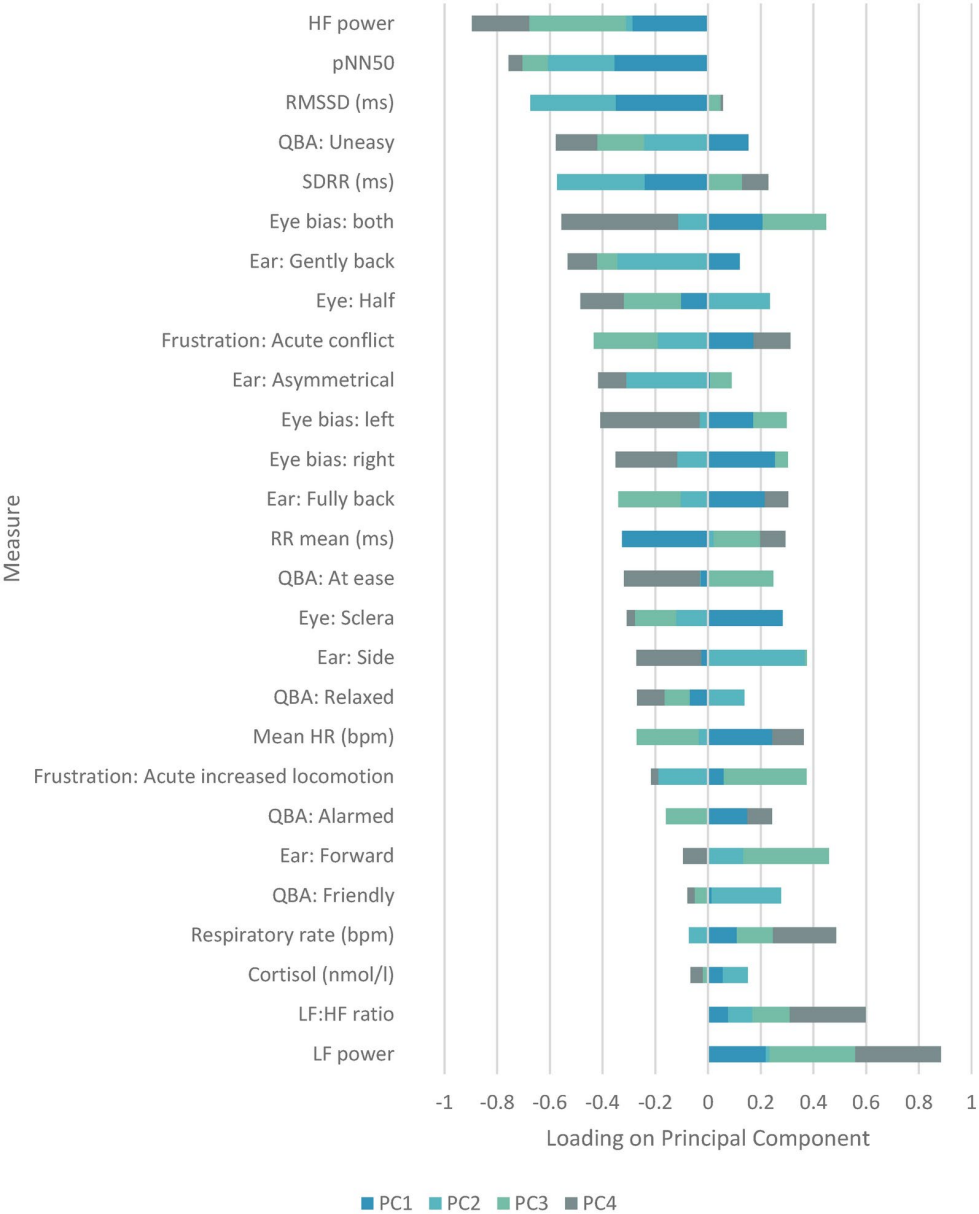
Loadings of behavioural and physiological measures on principal components 1 to 4.

Principal components three and four may represent elements of activation such as seeking (goal-directed engagement with the environment^98^) and conflict (engaging and protective emotions generated by the same experience^99^) potentially representative of positive and negative affective states respectively. PC 3 may be indicative of the activation of seeking type states, potentially in positive valence when aligned with positive PC2 loadings. PC 4 may be indicative of conflicted emotions, potentially in high arousal when aligned with positive PC 1 loadings (Fig. 3). PC 5 appeared unimportant with no trends identified.

### Linear modelling of PCA components by stimulus and trial

**Fig. 4:**
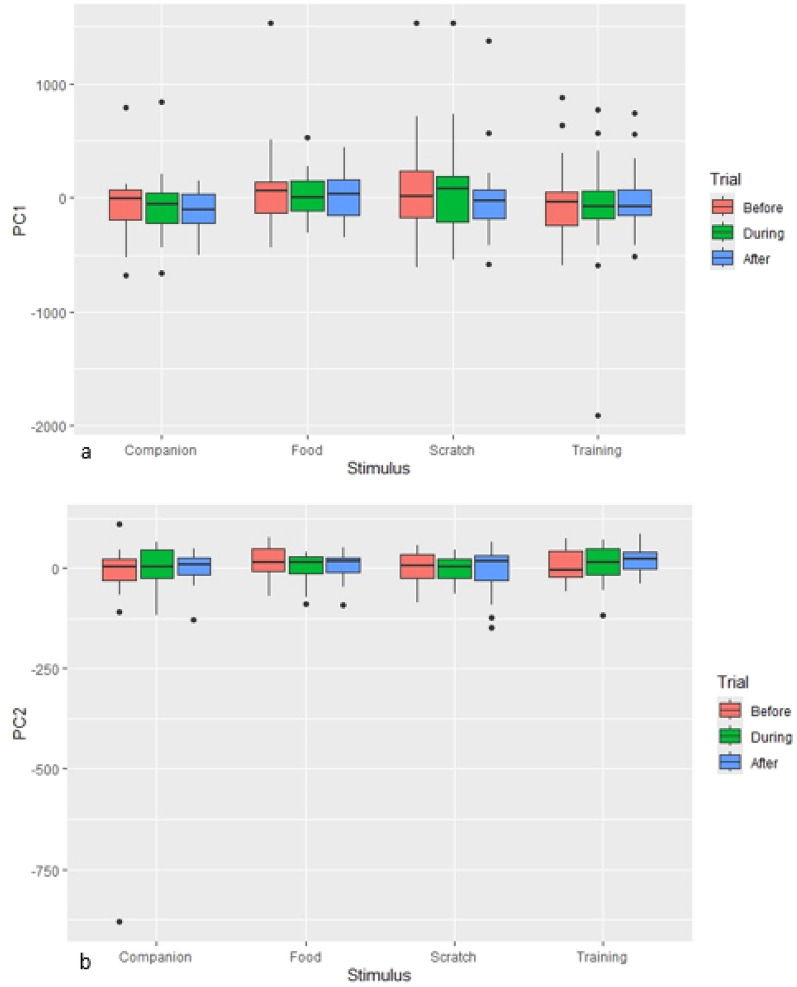
(**a**) Box and whisker plot displaying loadings on PC1 accounting for the effect of stimulus and trial and (**b**) Box and whisker plot displaying loadings on PC2 accounting for the effect of stimulus and trial.

Considering the hypothesis that the presentation and subsequent removal of the four induction stimuli would induce differing levels of arousal, and types of valence (positive or negative), linear models run on PCA components, did not find consistent changes in components according to induction stimulus and phase. A small, effect of stimulus type was identified within principal component 1 (F = 2.5609, df = 3, *p* = 0.55; Fig. 4a) but no effect of trial phase was found. No significant effects of trial or phase were identified for principal component 2 (Fig. 4b).

To investigate the hypothesis that affective states (differences in types of valence and levels of arousal) would be evidenced through differences in the behavioural and physiological responses recorded between induction stimuli, and during the phases of the trial (during and after presentation of the stimulus) a mixed model was run, controlling for the effect of individual horses (as random effects), on principal component 1 (arousal) which identified a significant difference, with a large effect size (F value greater than 4) between induction stimuli (Type iii ANOVA with Satterthwaite’s method, Stimulus: F = 4.3784, df 3, *p* = 0.005) with the behavioural and physiological responses to the induction stimulus ‘wither scratching’ significantly different (with a large effect size, t value greater than 2) when compared with the other three induction stimuli (t = 2.702, df 193.554, *p* = 0.007). It was identified via evaluation of boxplots (See supplementary material S3) that the frequency rate per minute of the behavioural measures ‘ears gently back’, ‘ears asymmetrical’, ‘eye half’ and ‘QBA relaxed’ were higher during wither scratching when compared with the other three induction stimuli. However, the ‘ears side’ measure was lower during wither scratching than for the other three induction stimuli. Physiological measure ‘salivary cortisol’ was lower during wither scratching and the measures ‘mean heart rate’ and ‘pNN50’ were lower after wither scratching compared to the other three induction stimuli. ‘Ears gently back’, ‘eye half’, ‘QBA relaxed’ and ‘QBA at ease’ remained higher after wither scratching compared to the other three induction stimuli. Variable ‘RR mean’ showed a greater distribution during and after wither scratching compared with the other three induction stimuli.

Within principal component 2 (valence) there was no significant difference in the behavioural and physiological measures between all trial phases and induction stimuli overall (Type iii ANOVA with Satterthwaite’s method, Trial: F = 0.5811, df 193.44, *p* = 0.56; Stimulus: F = 1.3920, df 194.18, *p* = 0.25). However, there was a significant effect of trial phase on component 2 for ‘trial during’ (t = 2.525, df 192.86, *p* = 0.01), ‘trial after’ (t = 2.130, df 193.42, *p* = 0.03), and a significant effect of ‘stimulus food’ (t = 2.898, df 193.81, *p* = 0.004), ‘stimulus scratch’ (t = 2.406, df 193.54, *p* = 0.01) and ‘stimulus training’ (t = 2.425, df 193.19, *p* = 0.01); particularly ‘trial during stimulus food’ (t = − 2.223, df 192.97, *p* = 0.02), ‘trial after stimulus food’ (t = − 2.065, df 193.73, *p* = 0.04) and ‘trial during stimulus scratch (t = − 1.995, df 192.86, *p* = 0.04), indicating that these induction methods and trial phases generated different behavioural and physiological responses across the cohort. Evaluation of boxplots (See supplementary material S3) identified that the behavioural variables ‘ears forward’, ‘gently back’ and ‘asymmetrical’ were lower during the food stimulus compared with the other three induction stimuli. ‘Eye bias both’ and ‘left’ were lower during the food stimuli than during the other three induction stimuli, as was ‘QBA at ease’. After the food stimulus the measures ‘ears forward’, ‘gently back’ and ‘asymmetrical’ were lower than for the other three induction stimuli while ‘frustration acute locomotion’ and ‘HF power’ were higher after the food stimulus compared with after the companion, training and wither scratching trials. During the wither scratching trial, it was identified that there were some high outliers within the measure ‘eye sclera’ alongside a larger ‘mean heart rate’ and ‘RR mean’ distribution and low ‘RMSSD’ distribution. Other differences in measures of note were a high frequency of ‘eye bias right’, ‘frustration acute conflict’ and ‘QBA uneasy’ during the companion stimulus. Interestingly the measure ‘frustration acute conflict’ increased after all four induction stimuli were removed.

### Linear modelling of PCA components by personality group

To investigate the hypothesis that the affective states produced via each of the four induction methods may, in part, be affected by individual personality traits mixed linear models were run to consider the effect of personality on equine responses to trial phase and stimulus which identified some effects of personality traits on components as combined measures of affect (Table 2). These are proposed as increases in arousal displayed by horses within certain personality traits (unfriendly, unresponsive, nervous) to some induction stimuli. Additionally, increases in positive valence are suggested within some personality traits (bold, unresponsive) in response to certain induction stimuli.

**Table 2:** Results of linear models examining the effect of personality traits on responses to stimuli.

Principal component	Independent variable	Personality trait	Overall model result	Effect variable	Effect result	Suggested affective influence
**PC1**	**Stimulus**	**Unfriendly**	**F = 5.8926, df = 3, p < 0.001**	**Scratch**	**t = 0.610, df = 199.44, p < 0.001**	**Increased arousal to scratch**
PC1	Stimulus	Affiliative	F = 0.5222, df = 3, p = 0.667	Scratch	t = 2.056, df = 199.68, p < 0.05	Increased arousal to scratch
PC1	Stimulus	Bold	F = 0.6095, df = 3, p = 0.6096	Food	t = 1.993, df = 199.80, p < 0.05	Increased arousal to food
**PC1**	**Stimulus**	**Unresponsive**	**F = 2.7937, df = 3, p < 0.05**	**Training**	**t = 2.889, df = 200.27, p < 0.05**	**Increased arousal to training**
**PC1**	**Stimulus**	**Nervous**	**F = 8.7709, df = 3, p < 0.001**	**Food**	**t = 2.765, df = 199.991, p < 0.01**	**Increased arousal to food**
**PC1**	**Stimulus**	**Nervous**	**F = 8.7709, df = 3, p < 0.001**	**Scratch**	**t = 4.365, df = 200.533, p < 0.001**	**Increased arousal to scratch**
PC2	Stimulus	Exuberant	F = 0.5272, df = 3, p = 0.664	Companion	t = -2.630, df 74.142, p = 0.01	Decreased positive valence to companion
**PC2**	**Stimulus**	**Bold**	**F = 2.3902, df = 3, p = 0.06**	**Training**	**t = 2.633, df = 199.130, p < 0.01**	Increased positive valence to training
**PC2**	**Stimulus**	**Bold**	**F = 2.3902, df = 3, p = 0.06**	**Companion**	**t = -2.073, df = 84.244, p < 0.05**	Decreased positive valence to companion
**PC2**	**Stimulus**	**Unresponsive**	**F = 2.3713, df = 3, p = 0.07**	**Training**	**t = 2.542, df = 201.695, p = 0.01**	Increased positive valence to training
PC2	Stimulus	Nervous	F = 1.7283, df = 3, p = 0.16	Companion	t = -1.999, df = 85.500, p < 0.05	Decreased positive valence to companion
PC2	Stimulus	Nervous	F = 1.7283, df = 3, p = 0.16	Training	t = 2.180, df = 200.734, p < 0.05	Increased positive valence to training
PC2	Trial	Unfriendly	F = 2.4939, df = 2, p = 0.08	After	t = 2.184, df = 204.779, p < 0.05	Increased positive valence after induction stimuli

Results where the overall model was significant are highlighted in bold.

## Discussion

The aim of this study was to induce positive affective state in a cohort of horses to enable identification of potential measures of positive affective state and to differentiate these from potential markers of negative affective state. It was recognised a priori that the induction of positive affect would likely differ between individuals, due in part to their personality traits, as well as the impact of prior life experience, current environment, and learning. The results of this study have identified that the measures utilised to attempt to evaluate affective state in equines appear to be reliable in terms of their inter and intra-rater reliability. Principal component analysis has identified clusters of variables that appear to be informative regarding the affective state of the equines within this study and the variables appear to cluster along the axes of principal components that could be described as valence and arousal components. From the results it appears that some behavioural measures may be useful proxies for their physiological counterparts within the PCA clusters (for example respiratory rate and eye bias left are clustered, as to some extent are eye half, ear side, QBA relaxed and at ease with RR mean and HF power) and there appears to be an effect of individual personality on equine responses to the four induction stimuli.

Whilst intra-rater agreement was excellent across the respiratory rate measurements these values were not externally tested through inter-rater agreement and as such this reduces the external validity of this measure within this trial. Behavioural data measurements appear extremely reliable, with excellent agreement across all five raters, likely related to the stringent methodology applied for the scoring of behavioural data. Given the range of experience between the raters (from behavioural specialists to raters with no behavioural or equine specialist knowledge), these results appear promising for future use in horses, when behavioural measures are evaluated alongside other parameters.

Prior research has identified LDA analysis as an optimal method for data reduction when taking into account sensitivity, specificity and overall classification accuracy of both physiological and behavioural scientific measures^100^. Here we used wither scratching as the best validated method of inducing affect to identify which measures to apply across all data to make component scores representing aspects of core affect. Wither scratching was selected due to the evidence within the literature^19,20,45,46^ and the high rank of this as an induction stimulus by the Delphi experts^57^. The study supported wither scratching as a method for inducing affect since the potential measures of affect were separate between phases of before and during wither scratching when exposed to LDA. A limitation of this approach is that some of the measures could have been specific to wither scratching and therefore less applicable as general indicators of positive affective state.

Examination of the PCA loading plot output identified what appears to be a clear separation between measures that are reported in the literature to account for high and low levels of arousal in PC1 and indicative of positive and negative valence in PC2 (see additional discussion of measures in supplementary material S4). This study was designed around the premise of the core affect 2-dimensional model^2,101,102^ with the proposal that different physiological and behavioural measures may be identified which could be proposed as potential measures of varying affective states. The output derived from the PCA plot provides an initial starting point for further exploration of the identified measures of affective state across the four quadrants.

It is postulated that components three and four may be linked to seeking-type motivation and conflicted emotions respectively. Further research could investigate this by measuring plasma dopamine which is positively associated with seeking behaviours in other species^103^ and negatively associated with fearfulness in horses^104^. Variables loading positively on PC4 appear to relate to reward acquisition and punishment avoidance^87,90,105^ which could be further investigated using reward acquisition or risk-based tasks. This is particularly relevant where frustration may be evident in behavioural outputs^28^, as was seen in some equines within this study (with the measure ‘acute conflict’ increasing after each of the stimuli were removed), which may be related to seeking a different type of interaction (for example with the conspecific) or the removal of reinforcement (for example high value food or the cessation of training). Additionally conflicted emotions, potentially related to con- or hetero-specific interactions during the trial may heighten arousal (potentially with negative valence) thereby dampening any positive effects of the stimulus^106,107^. These are facets that should be considered in future studies.

Interpretation of PCA data is somewhat subjective in terms of attributing meaning to the principal components produced. Relative loadings on components indicates that the variables vary together^108^, however, the interpretations made here may not be valid, either due to the over or under estimation of the significance of individual variables or due to inconsistencies that arise within the literature in relation to the potential meaning of variables.

The components did not consistently change in response to the induction methods making it difficult to interpret whether the components were capturing changes in affect, or whether the affective states assumed were not being induced. The four selected induction stimuli had valid rationales for their inclusion however some equines may have found other induction stimuli more intrinsically reinforcing or less negatively valenced or arousing than those that were selected^109–111^. Individual difference was important in the statistical models, and personality was associated with component scores for some stimuli. This supports our earlier Delphi study^57^ in which experts expressed opinions that personality was an important aspect to consider with respect to affective state in horses. Results indicate that consideration should be given to individual personality differences in equines during handling, training and enrichment processes which may alter affective state. Influential personality traits may augment arousal and valence relating to the properties of differing reinforcers meaning that careful examination of personality may enable selection of optimal reinforcement strategies for the individual. For example, our results suggest that equines high in trait ‘unresponsive’ may show increased arousal in positive valence to target training using positive reinforcement methods, a facet that could be well utilised to improve affective state in these types of equines.

Personality assessment was carried out by owners/carers which may lead to bias. However, these horses and their scores were included in another study^76^ where scores were evaluated for both inter and intra-rater reliability, as well as comparisons made between a short and long form of the personality questionnaire; with agreement deemed to range between good and excellent, strengthening the support for the use of these measures within the current study. Additionally, trait ratings such as these have been demonstrated to be substantially more reliable than behaviour coding for assessment of animal personality^112^. It is important to acknowledge the potential for subjective bias within owner scoring of their own animal’s personality traits, which may impact upon the reliability of their ratings^113^. Additionally, individual scorers may interpret the personality descriptors differently which may lead to increased variation in scores. Within future research independent personality raters could be included to further test for inter-rater variability and improve the objectivity of personality evaluations, providing that the raters were sufficiently familiar with each study subject. Furthermore cross-validation of personality assessments with behavioural observations during affect induction could strengthen future studies^114^.

An important consideration, which is a limitation within this study, is the circular argument that exists where a set of measures were selected to identify positive affective state produced using the selected induction methods where neither measures nor induction methods are all validated. We took several steps to minimise circularity. Firstly, we used a combination of measures which had stronger and weaker evidence that they were related to affective state in horses. By investigating correlations using the PCA between similar (convergent) and dissimilar (divergent) measures we provide a level of concurrent criterion validity (see Williams et al.^115^ for how validity terms can be applied to welfare measures). The prior Delphi expert panel^57^ could be considered as providing face validity for both measures and induction techniques. By using the best validated induction method, wither scratching, to select measures to take forwards there is a level of concurrent validity. Further studies could look to disentangle arousal and valence components in relation to positive and negative affective states whilst also considering the proposition of neutral affect further within their methodologies. Further investigation of the potential independent, positive linear, symmetric and asymmetric relationships between arousal and valence components, including positivity offset and negativity bias are important facets to consider in this sphere^116^. The identification of physiological and behavioural markers which may be discrete to valence or arousal components could be a useful next step to further understand and assess affective state in equines.

This exploratory study has several limitations which limit the reliability and generalisability of the results reported. The sample size within this study is relatively modest at 20 equines, however, studies like this one have sample sizes within the same range (13–32 horses)^20,43,80,117^. The sample within this study was broadly evenly distributed across sexes, age (young = 0–4 years, mature = 5–15 years and aged = 15 + years) and represented a range of breeds including pure and mixed-breed horses which aids generalisability of the results across equine demographics, but the low sample size prohibits wider generalisation and further analysis of the effects of these demographic differences within the statistical modelling. Further data collection using the same methodological design would add greater power to the study, analysis of a larger dataset may provide instructive evidence around the strength and stability of the observed measurement clusters and retesting of the horses utilised within the current study on at least two further occasions would enable evaluation of the temporal stability of the results reported within this trial. It would be beneficial for researchers in this area to collaborate more widely on a large-scale project to collect a substantial dataset of wide-ranging measurement and induction methods across a much larger cohort of equines to allow for the generation of reliable evidence in this field.

Furthermore, several potential induction stimuli were omitted from the methodological design due to the perceived difficulty in recording the selected behavioural and physiological measures during their implementation. The four selected induction stimuli had valid rationales for their inclusion however it is possible that some equines may have found some of the omitted induction stimuli more intrinsically reinforcing or less negatively valenced or arousing than those that were selected. In particular, some ethologically relevant induction stimuli such as access to graze and browse, turnout and horse-horse interaction at liberty may have provided further useful data in relation to the physiological and behavioural responses to these stimuli and further work could focus on the attempted induction of positive affect using only at liberty, ethologically relevant induction stimuli to reduce the potentially negative effects of confinement and restraint on the achievement of positive affect^109–111^.

## Conclusion

This study has generated a dataset which provides further advancement of our knowledge around the induction of positive affect and consideration of the meaning and usefulness of variables used to attempt to measure affective state in equines.

The approach was exploratory and provides a novel template for the study of affective state, where there are gaps in knowledge about both methods to induce and the parameters used to measure affective state. In combination the novel aspects of the study were: (i) Selection of measures based on expert opinion and support from the literature; (ii) Dimension reduction based on the best validated induction methods and comparison with the best validated measures to date; (iii) Inclusion of personality scoring to account for a significant factor likely to lead to variation in equine responses to induction stimuli.

The findings of this work suggest that there is variation within the equine cohort in terms of their physiological and behavioural responses to the induction stimuli that, to some extent, is influenced by personality. The results also suggest variation in the usefulness of the measures utilised for identifying different types of affective state and the dimensional approach taken has allowed for assessment of the broad arousal (high and low) and valence (positive and negative) components of affect without ascribing measures to discrete emotions such as fear for example.

This study is relevant in the current climate where social license to operate has become a significant factor within the equine industry, within which equine welfare is being scrutinised, especially within the competitive sphere. Increased knowledge of how to induce positive affect in horses could improve their welfare and thus prevent the development of pre-pathological and pathological conditions resulting from chronic impaired emotional welfare.

## Methods

### Ethical approval

The protocol and methods utilised throughout this study were approved by the Animal Welfare Ethics Review Board (AWERB) of Newcastle University (Project ID: 1025), complied with the AWERB guidelines and conformed with ARRIVE guidelines.

### Outcomes from the pilot study

A pilot study consisting of 26 horses recruited, with consent, from two university sites was undertaken for refinement of the methodology employed in this study including the consideration of study area set-up, wash-out period between treatments and methods utilised for data processing and analysis. The results of the pilot study identified some interesting trends in relation to changes in both physiological and behavioural parameters in response to different induction stimuli and highlighted the requirement for preference testing of induction methods within the trial to select methods most positively salient to each equine. Furthermore, the pilot study highlighted that a multi-measure approach to measuring affective state would be more reliable than looking at individual measures in isolation.

### Trial preparation and subject recruitment

Equine subjects were recruited via individual invitations to their owners. Informed consent was confirmed, and pre-trial demographic and prior history information was gathered for all equines via an online form (See supplementary material S5). Equine personality scoring was undertaken pre-trial, using the Equine Personality Eight-Factor Model (EPEM) full and abridged scoring system^76^.

Trials for each equine were conducted across two consecutive days and data collection ran from June to August 2023. Prior to the trial days each subject underwent a food and wither scratching preference testing protocol prior to data collection (See supplementary material, S6).

### Subjects

Twenty healthy horses were recruited to the study, from five independent facilities, between March and May 2023 (Table 3).

**Table 3:** Demographic data for the twenty subjects including test location.

Horse number	Age (years)	Breed	Sex	Use	Time in ownership or care	Test location
1	3	Mini Cob	Gelding	Non-ridden	> 6 months < 1 year	1
2	9	Thoroughbred	Gelding	Unaffiliated competition	> 6 years	1
3	5	Irish x Thoroughbred x Arab x Trakehner	Mare	Unaffiliated competition	5–6 years	2
4	16	Warmblood	Gelding	Non-ridden	> 6 months < 1 year	2
5	11	Native crossbreed	Gelding	Unaffiliated competition	1–2 years	2
6	25	Highland	Mare	Hacking	> 6 years	3
7	28	Native crossbreed	Gelding	Non-ridden	> 6 years	3
8	9	Thoroughbred	Gelding	Elite competition	> 6 years	4
9	10	Thoroughbred	Gelding	Elite competition	> 6 years	4
10	4	Thoroughbred	Gelding	Elite competition	1–2 years	4
11	10	Thoroughbred	Gelding	Elite competition	> 6 years	4
12	5	Thoroughbred	Gelding	Elite competition	1–2 years	4
13	26	Cob	Mare	Hacking	> 6 years	5
14	30	Connemara cross	Mare	Non-ridden	> 6 years	5
15	18	Native crossbreed	Mare	Non-ridden	> 6 years	5
16	6	Thoroughbred	Mare	Elite competition	1–2 years	4
17	6	Thoroughbred	Mare	Elite competition	1–2 years	4
18	5	Thoroughbred	Mare	Elite competition	> 6 months < 1 year	4
19	6	Thoroughbred	Gelding	Elite competition	1–2 years	4
20	7	Thoroughbred	Gelding	Elite competition	3–4 years	4

### Trial area set up and habituation

**Fig. 5:**
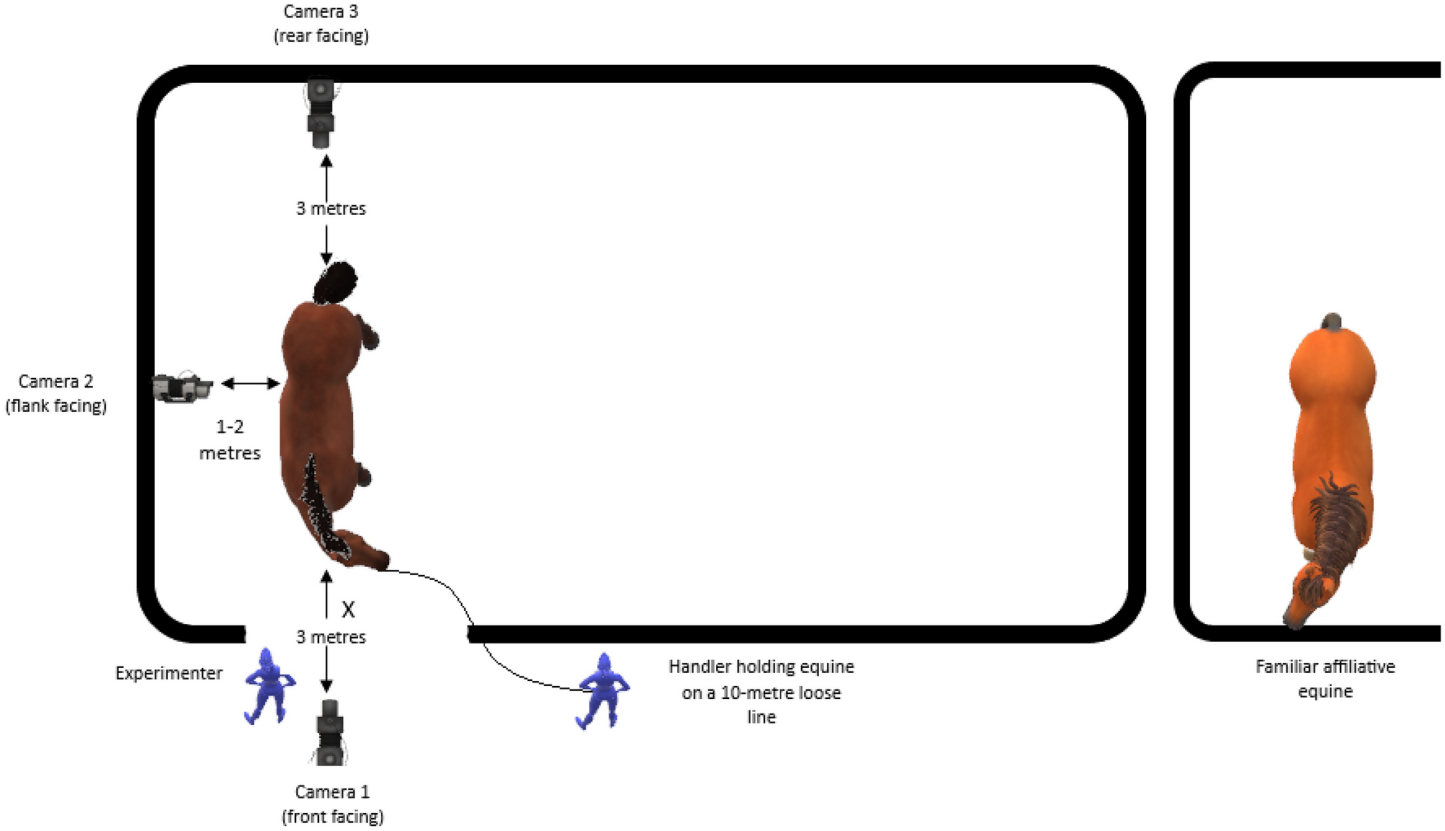
Trial area set up which was replicated at each location. X marks the position that thermographic images were taken from (1 m in front of the equine).

The trial area was set up in an identical manner at each of the five test locations (Fig. 5) with three GoPro Hero 8 cameras (GoPro Inc, USA) placed 3 m to the front of the horse, 1–2 m to the side of the horse and 3 m to the rear of the horse to capture a wide field of view and enable scoring of behavioural and respiratory data.

A thermography camera FLIR T430SC (FLIR LLC, USA) was used to collect thermography data and was time paired with both the GoPro cameras and heart rate monitor. All equines were habituated to the area used for data collection and the equipment used on at least three occasions 24–72 h prior to the trials. Habituation was deemed to have occurred when heart rate did not increase more than 20 beats per minute from baseline during the habituation period^118^ and when they quickly (within 1 min) showed postural (relaxed muscle tone, low head carriage) and behavioural (standing and eating hay or resting/dozing) signs of relaxation on entering the area. Horses who did not habituate to the stable area were to be excluded from the trial, however all equines habituated well. All subjects were held on a ~ 10 m loose lead rope for each trial by a known handler to avoid potential stress responses confounding results^119^. The handler was instructed not to interact with the horse during the trial (apart from to place the equine in the correct location for filming) and stood outside the stable and to the right of the closed stable door or through another form of barrier to reduce handler effect^120,121^. A familiar affiliative equine was placed nearby (approximately 10–15 m) during the entire trial to avoid isolation stress^20,122^ but not so close as to confound the ‘affiliative companion’ induction test.

### Test procedure

**Fig. 6:**
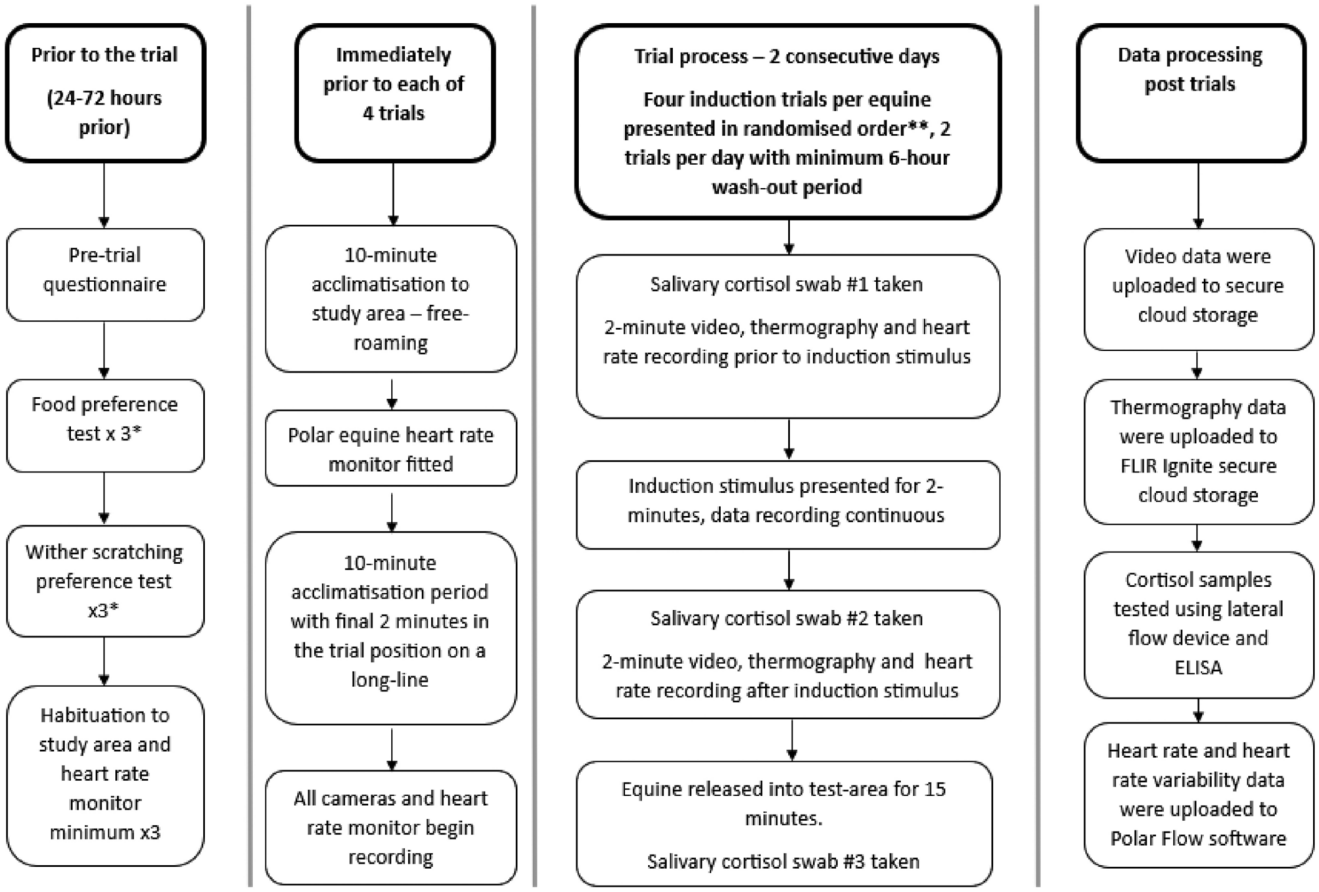
Schema depicting the pre, during and post-trial processes. * denotes that full methods and results for the preference tests can be found in the supplementary material S6, ** denotes that induction trial stimuli (wither scratching, high value food provision, target training and addition of an affiliative companion) presentation order (randomised) can be found in supplementary material S7.

The test protocol was identical for each equine. The subject was led to the research area by a known handler and handled by this handler throughout each trial. The trial process is outlined in full in Fig. 6.

### Induction methods

To maximise the likelihood of inducing positive affect in the equine cohort within this study the induction stimuli utilised were those rated most highly by the expert group^57^ and supported by the literature. The cross-applicability of induction methods was also considered within the selection process whereby methods that may be highly appetitive to equines but only in a specific or limited circumstance (such as access to a substrate to roll in) were excluded. This process resulted in the selection of the following four induction methods: (i) High value food provision, (ii) The addition of an affiliative conspecific, (iii) Wither scratching and (iv) Application of positive training via target training.

#### Addition of an affiliative conspecific

Ranked second by the experts and supported within the ethological literature^62–64^, this measure was included within the study as a potential induction method for positive affect in low arousal in equines. To utilise the addition of a conspecific to induce positive affect in equines an understanding the nature of the relationship between the two parties is critical.

Within the trial the affiliative conspecific was defined as such following observation of at least five occasions of allogrooming^64^ and/or affiliative interactions such as play^64,123^ and assessment of proximity to conspecifics, duration of proximity, and the performance of active affiliative behavioural expressions; such as approaches^63,124^ with the subject horse in the 4 weeks preceding the trial. The conspecific was brought into view and walked up to the subject horse whereupon the two horses were free to choose to interact or not for the two-minute trial duration. Following this the conspecific was led away out of view.

#### High value feedstuff

As the highest ranked induction method^57^ and one well supported by the literature^59,125^ this method was included within the study. This induction stimulus was of particular interest due to its high value, ethologically, to equines^58^ but also due to the potential for it to induce alternative affective responses to those aimed for within the study. In addition, the relative value of perceived high value feedstuff within individuals, according to their personality and prior experiences, was deemed likely to result in a range of affective experiences to induction using this method^61^, a factor which was identified as potentially useful within later modelling of the measures employed to evaluate affective state achieved. Following preference testing (See supplementary material S6), this trial included the presentation of 1 kg of the preferred substrate in one of the preconditioned buckets^126^ which the horse could eat continuously for the two-minute trial period, after which the stimulus was removed.

#### Wither scratching

Whilst ranked fourth by the experts^57^, wither scratching is highly supported within the equine literature as a phenomenon that is broadly experienced as positive by equines. It is thought to contribute to relationship building and comfort^127,128^ and has been observed between heterospecifics^58,72^ with research identifying links between grooming and/or massaging around the wither area and reduction in heart rate combined with relaxed behavioural responses^19,20,70,71^. Therefore, wither scratching may induce a positive affective state similar to that seen in allogrooming. Prior work has demonstrated a preference for scratching of the wither over other areas such as the neck or face^45^ and as such this was selected as the focal scratching area within this study. Within this study wither scratching was trailed with all subjects prior to the trial (See supplementary material S6), and the preferred method and exact site was utilised for each individual subject. The subject received wither scratching, from their known handler, for the entirety of the two-minute trial period and it then abruptly stopped.

#### Positive reinforcement training

Ranked 9th by the experts^57^, this method was deemed to be a novel method which could have good application for inducing positive affect but one that is not widely researched to date. This technique has been previously proposed as a method to optimise equine welfare^66^ and although it remains unutilised within equine studies attempting to induce positive affect there is evidence of its efficacy in dogs and wolves^129^. Furthermore, in pigs enhanced attention and cognitive activity has been shown to induce greater positive emotional states^130^, a finding that may be transferable to equines and one considered by Sankey et al.^67,68^ within their studies looking at reinforcement and positive long-lasting memories in equines, indicating that human attention may be a source of positive emotions for equines. Each equine undertook target training, completed by one of the researchers (LL), for a two-minute duration for this induction trial. The equine was presented with the target stick and upon touching the target with the muzzle, a previously conditioned secondary marker (verbal ‘yes’ or ‘good’) was utilised prior to withdrawal of the target and delivery of a low value food reinforcer (chaff) to negate, as much as possible, any crossover in effect between this trial and the high value food trial^59^. Following which the target was presented again to the equine; this process continued for the two-minute trial duration, following which the trainer moved away.

### Behavioural and physiological measures

In an earlier phase of this research, we conducted a Delphi consultation^57^ to support selection of measures of positive affect for this study. Feedback from the experts indicated consensus agreement that heart rate variability, salivary cortisol, respiratory rate, body language, facial action scoring and qualitative behaviour assessment were reliable methods to assess emotional state in equines. However, many participants commented on the promise but lack of validation of newer methods (eye bias or lateralisation, infra-red thermography of the eye and ear). As such it was decided in this study to utilise a combination of measures which had lesser or greater evidence to validate them as measures of positive affective state and understand how measures covary.

### Behavioural measures

#### Ear and eye position

Ear position (forward, asymmetrical, sideways, gently back, fully back) data were scored for both frequency and duration via continuous recording. Eye positions ‘open’, ‘half open’ and ‘sclera’ were scored for frequency and duration via continuous recording (See supplementary material S8).

#### Eye bias (lateralisation)

Eye bias was scored continuously for left, both and right eye bias (predominant focus with the left or right eye, or both eyes), with scoring of both duration and frequency undertaken (See supplementary material S8).

#### Qualitative Behaviour Assessment (QBA)

QBA assessment was undertaken continuously using the full AWIN QBA 13-item descriptor list^95^ with both frequency and duration of any behaviours identified (binary presence/absence of the descriptor) recorded for each horse across all trials (See supplementary material S9).

#### Indicators of frustration

This measure utilised the ten descriptors reported by Pannewitz and Loftus^28^ and scored for both frequency and duration of occurrence via continuous sampling^131^, (See supplementary material S10).

### Physiological measures

#### Heart rate and heart rate variability (HRV)

From a methodological perspective it is important that when HRV is used to assess emotional state, inter-beat-interval (IBI) data lasting at least five minutes consecutively is measured during stationary conditions to negate the effect of movement behaviour on cardiac function as well as pre-behavioural physiological anticipatory responses and persisting post-behavioural cardiac responses^132^. To satisfy these requirements both heart rate and heart rate variability were recorded continually from the start to the end of each trial phase and for a minimum of 2 min per phase of each trial giving a minimum recording time of 6 min and a maximum of 12 min within this study. All data were recorded using the Polar V800 device and Polar H10 Heart rate sensor band.

#### Respiratory rate

Respiratory rate was scored via video footage where the rise and fall of the flank was counted as 1 unit. A period of 30 s was scored within the middle section of each trial phase across each induction stimulus for each horse. Scoring was repeated later by the same rater to assess intra-rater reliability.

#### Salivary cortisol

Within this study each horse acted as its own control for each trial with salivary samples taken before each trial as well as immediately after and 15 min after each trial following a similar test protocol to Kedzierski et al.^133^. Soma equine salivary cortisol test kits (Soma Bioscience, Wallingford, UK) were utilised to take salivary cortisol samples alongside the Soma Cube reader (Soma Bioscience, Wallingford, UK) which analysed lateral flow device (LFD) samples at penside (See supplementary material S11). Subsequently all samples were laboratory analysed via Enzyme Linked Immunosorbent Assay (ELISA)^134^ by Soma Bioscience to evaluate the reliability of the LFD analysis.

#### Ear and eye temperature

Within this study eye and ear temperature were recorded using a Flir T430SC (FLIR Systems AB, Danderyd, Sweden) camera on a moveable tripod set one metre away from the subject. Data were recorded at ~ ten second intervals, and data files uploaded to Flir Ignite online software for analysis. The camera was calibrated to the observation distance, temperature and relative humidity and recalibrated for each subject. Still images of the medial posterior palpebral border of the lower eyelid and lacrimal caruncle of the left and right eyes^135^ and the base of each ear^43^ were taken at a 90-degree angle in relation to the sagittal plane^43,83^ around every 10 s when the horses head was in the optimal position for imaging (example in supplementary material S12).

### Personality assessment

Personality assessment was undertaken for each horse as per the method outlined in Loftus et al.^76^ All twenty horses included in this final study were rated by their owners or carers on all the individual personality traits and using the full version of the questionnaire and via the abridged questionnaire.

### Data processing and statistical analysis

#### Data processing

*Cortisol data*. Cronbach’s Alpha reliability analysis was undertaken to compare the LFD and Elisa results which showed moderate agreement (*r*^2^ = 0.4, *p* < 0.001). Therefore to ensure accuracy the Elisa results were taken forward for analysis.

*Heart rate data*. Heart rate data were extracted from Polar Flow individually per horse, per stimulus. The data were then uploaded to Kubios version 3.5.0 (Kubios OY, Finland)^136^. Data were treated using the method outlined by Scopa et al.^85^, initially the R peaks of the QRS complexes were determined using Kubios’ inbuilt QRS detector and the Pan-Tompkins algorithm method. Data were passed through a bandpass filter (frequency band of 0.05–32 Hz and window width of 150 ms) to smooth close-by peaks. Medium correction was utilised to avoid over-correcting and missing normal inter-beat intervals. Three spectral bands were identified that have been specifically recommended for equine HRV analysis^85^; Very Low-frequency (VLF) was set to 0–0.01 Hz, Low-frequency (LF) was set to 0.01–0.07 Hz and High-frequency was set to 0.07–0.6 Hz.

In the time domain, Mean RR, SDNN, Mean HR, SDHR, Minimum HR, Maximum HR, RMSSD and pNN50 data were extracted. In the frequency domain, AR values were extracted as these are most applicable for data collected over shorter timeframes. These included Peak (Hz), Power (ms^2^), Power (%) and Power (n.u.) for the VLF, LF and HF domains; LF-HF ratio was also computed. In the non-linear domain, SD1 and SD2 were computed.

*Respiratory rate data*. Respiratory rate was calculated from video data extracted from a side-facing GoPro camera which was mounted at a height and position to capture the rise and fall of the flanks. Each rise and fall were counted as one respiratory unit. Respiratory rate was counted using a four-digit clicker counter (Sdarming, Dongguan Xunze Trading Company, China) for 30 s in the middle section of each phase of each trial and the number was doubled to give a beat per minute value. Intra-observer reliability was tested through rescoring of 20% of the counts 2 weeks following the initial count.

*Thermography data*. Still images from each phase of each stimulus presentation were analysed using Flir Ignite software. Initially, images were inspected for clarity, focus and availability of measurement of each of the four data points. Eye temperature was taken from the medial canthus of the eye as suggested by Kim and Cho^135^.

*Behavioural data*. All behavioural measures were scored by the principal researcher (LL) using ethograms constructed prior to scoring (See supplementary material S8-10) and 20% of videos were tested for inter-rater reliability.

Behavioural data were scored from video using CowLog (v3.0.2) software^137,138^ and followed a predetermined method (See supplementary material S13).

Once scored the behavioural data were placed into Microsoft Excel v16 (Microsoft Corporation, 2016) with pre-set formulas which calculated total duration and frequency of each type of scored behaviour.

Inter-observer reliability was tested utilising intraclass correlation coefficient (ICC) analysis on data from 5 trained additional raters who between them re-scored 20% of the videos (at least one per horse and three per stimulus). Two raters were specialists in equine behaviour, two raters were equine specialists but not specialists in behaviour and the final rater was neither an equine nor a behaviour specialist. All raters received the same training, scoring software, ethograms and scoring instructions and all scored the videos independently of each other. Random allocation of videos to the raters was determined using online randomisation software (www.random.org).

### Statistical analysis

An exploratory approach was undertaken throughout the statistical analysis to attempt to identify behavioural and physiological markers of affective state (both positive and negative) and to consider the effect of the treatment (stimulus), personality and the random effect of the horse within analyses. Four a priori assumptions were made (i) There would be covariation of measures^139–142^; (ii) Wither scratching induces positive affect^19,20,70^, (iii) Personality would influence behavioural and physiological responses to different induction stimuli^143,144^ and iv) QBA measures are valid indicators due to their prior validation^13,95,145^. These assumptions were made following review of the literature which has previously identified covariation between some behavioural and physiological measures of equine affective state^19,20^ and identified wither scratching as a method which is likely to induce relaxation and positive emotions in horses^20,45,46,146^. Evidence from the literature also identified the likely influence of personality traits on equine behavioural responses^147–149^.

**Fig. 7:**
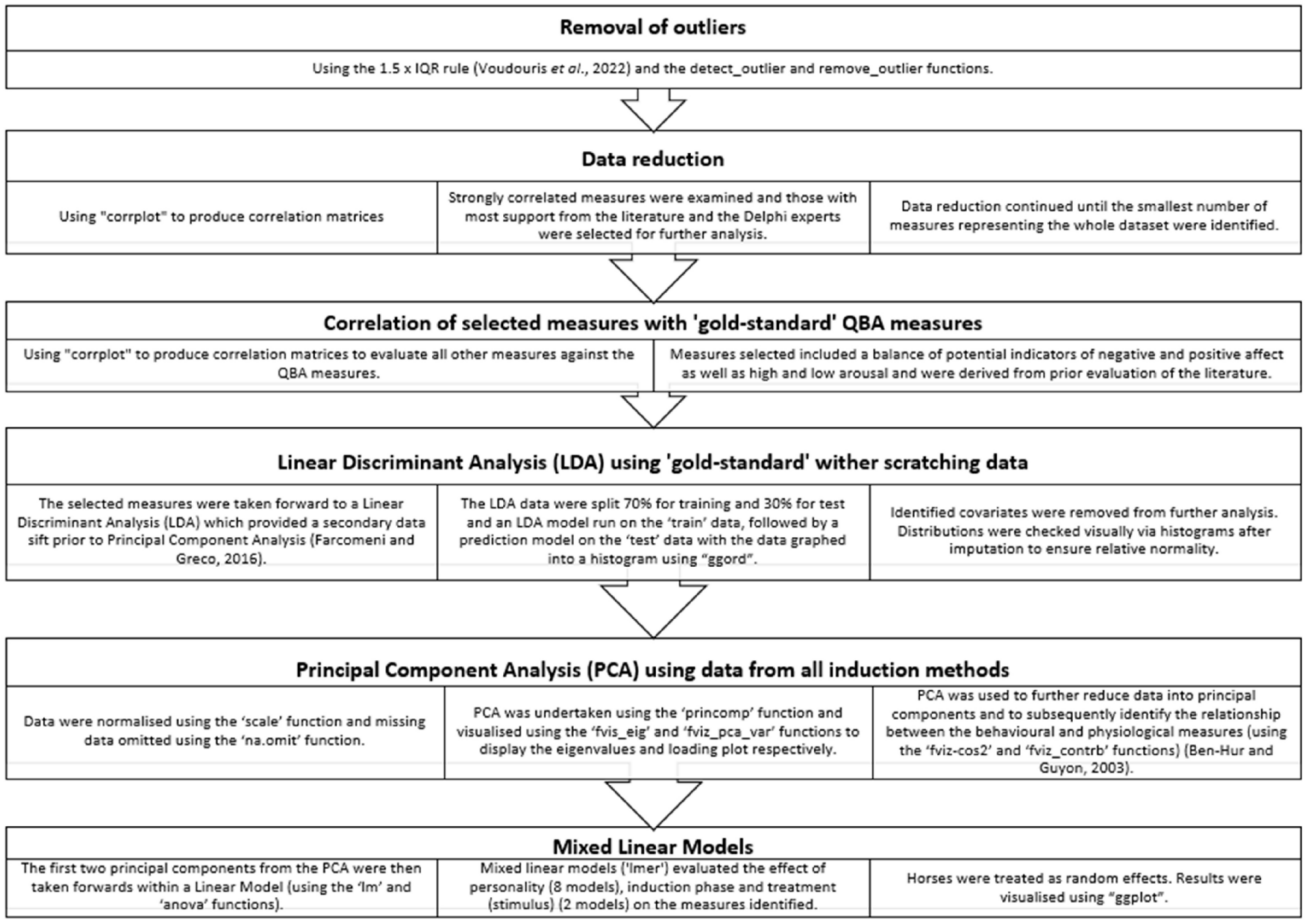
Flow of statistical analysis undertaken.

All analyses were undertaken using R Studio 12.1^150^ utilising the packages “car”, “corr”, “corrplot”, “devtools”, “FactoMineR”, “factoextra”, “fansi”, “ggcorrplot”, “ggord”, “KlaR”, “lme4”, “MASS” and “Psych”. Initially, outliers were removed from the dataset using the 1.5 × IQR rule^151^ and the detect_outlier and remove_outlier functions; following which correlation matrices were computed using “corrplot” to identify measures which appeared to covary to allow for later data reduction^152^ (See supplementary material S1). Variables with a high number of missing values (> 10 per variable) were excluded from analysis. This included several of the QBA and frustration measures and where variables contained low levels of missing values (≤ 10 per variable) the median value for the variable was computed and imputed in place of the missing values (this occurred mainly for thermography and HRV data). Distributions were checked visually via histograms after imputation to ensure relative normality. The statistical methodology is described in full in Fig. 7.

The QBA data frame was scaled and normalised. The well validated QBA measures^13,95,97^ were evaluated for correlations (using corrplot) with the other measures (ear data, eye data, frustration data and heart rate data) (See supplementary material S1). A balance of measures with both negative and positive correlations to the QBA variables were selected to be taken forward using evidence for their value from the literature. Following initial review of the relationships between variables it was noted that there did not seem to be sufficient variables included within the PCA to meaningfully distinguish between potential indicators of negative and positive affective states. Therefore, further variables were included within the PCA analysis (See supplementary material S2). Cortisol and respiratory rate data were also added as physiological variables of interest as highlighted by the literature and as there appeared to be some differences between treatments and trial phase within the cortisol and the respiratory data collected within this study (See supplementary material S14). As an additional data sift an LDA was run using only the wither scratch data with the rationale that wither scratching was the most highly validated induction method for calm, positive affect according to evidence from the literature^19,20,45,46^. The LDA was only run on phases ‘before’ and ‘during’ to avoid the impact of any carry over effect during phase ‘after’ which may have skewed the data^153^. Initially, linear models were run using principal components 1 and 2 to identify any effect of trial phase (before, during and after) and stimulus (food, wither scratching, companion and training) on the variables within each component. Following this, mixed linear models were run to identify any effect horse as a random effect (2 models, one for PC1 and one for PC2) on the treatments evaluated (trial phase and stimulus). Subsequently, eight mixed models were run for PC1 (one for each of the eight personality traits, which were considered fixed effects), and the same process was repeated for PC2 to identify any effect of personality trait on the variables within the principal components. Following the linear models on the principal components, data on relevant behaviours were visualised by stimulus and trial phase to gain more detail of which specific behaviour changed in response to the stimuli (See supplementary material S3).

## Supplementary Information

Supplementary Information.

## Data Availability

The dataset analysed during the current study is available from the corresponding author on reasonable request.
