# Supplementary material for: Exploring the induction and measurement of positive affective state in equines through a personality-centred lens
Source: Sci Rep. 2025 May 27;15:18550. doi: 10.1038/s41598-025-98034-8 (PMC12116887; doi:10.1038/s41598-025-98034-8)
Supplement: Supplementary file 1 — Supplementary Information. [file 41598_2025_98034_MOESM1_ESM.pdf]

Supplementary materials

S1 Corrplot with all variables included

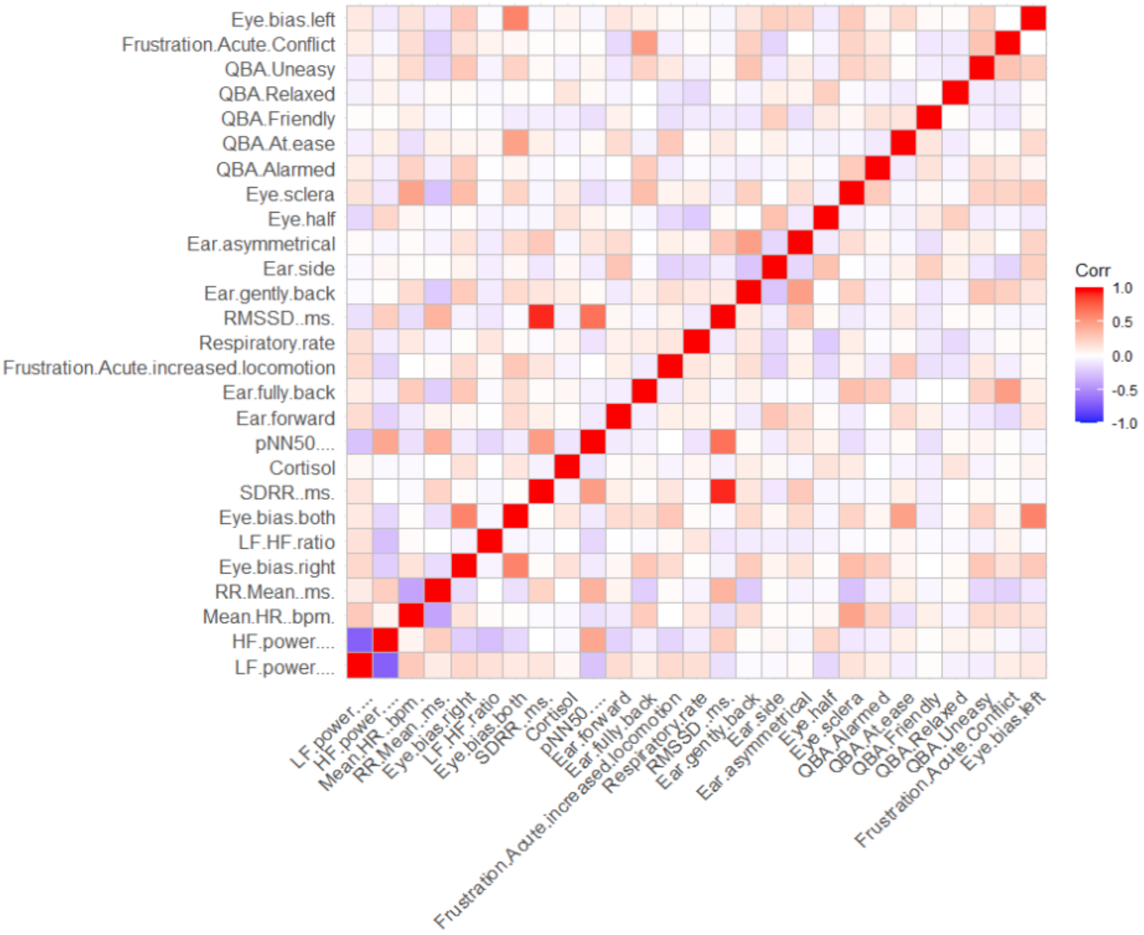

Correlation between all variables shown on a correlation plot. Positive correlation between SDRR and RMSSD identified by the bright red squares slightly above and below the central within variable correlation line. Negative correlations were identified, as anticipated, between low and high frequency power (darker purple squares to the bottom left of the plot).

S2 Measures taken forward for further analysis. Rationale for taking each measure forward is presented along with the results of the Pearson's product-moment correlation, the correlation estimate and whether the item positively or negatively correlated with the QBA measures.

Measure	Rationale	t value	df	p value	Correlation	+/- valence
Ears forward (rate per minute)	Included as a potential positive measure [1].	0.779	232	0.4363	0.051	+
Ears fully back (rate per minute)	Included as a potential negative measure [1].	-10.442	232	<0.001	-0.565	-
Eye bias both (rate per minute)	Included as one of the more highly negatively correlated measures [2].	-3.155	232	<0.01	-0.203	+/-
Eye bias right (rate per minute)	Included as one of the more highly negatively correlated measures. Inclusion support by the pilot study and Des Roches, Richard-Yris [2].	-4.051	232	<0.001	-0.257	+/-
Frustration: Acute increased locomotion (rate per minute)	Included as a positively correlated measure and potential negative affect measure [3].	0.985	232	<0.001	0.058	-

Mean heart rate (beats per minute)	Included as a positively correlated measure supported by Loftus, Marks [4].	3.052	228	0.002	0.198	**+/-**
RR mean (ms)	Included as a comparison to mean heart rate [5].	-1.240	228	0.216	-0.081	**+/-**
SDRR (ms)	Included as a negatively correlated measure. Inclusion support by the literature [5].	-0.038	228	0.969	-0.002	**+/-**
RMSSD (ms)	Included as a positively correlated measure. Inclusion support by the literature [5].	0.042	228	0.965	0.002	**+/-**
pNN50 (%)	Included as a positively correlated measure. Inclusion support by the literature [5].	0.950	228	0.343	0.062	**+/-**
Low frequency power (%)	Included as a positively correlated	0.494	228	0.621	0.032	**+/-**

	measure. Inclusion support by the literature [5].					
High frequency power (%)	Included as a positively correlated measure. Inclusion support by the literature [5].	51	228	0.725	0.023	**+/-**
Low frequency/high frequency ratio	Included as a negatively correlated measure. Inclusion support by the literature [5].	-0.334	228	0.738	-0.022	**+/-**

Additional variables added to PCA analysis with rationale for inclusion.

**Measure**	**Rationale**
Ears sideways (rate per minute)	Supported as a potential measure of affect by Bornmann, Randle [6] and Ricci-Bonot and Mills [7]. Action defined by Wathan, Burrows [8].
Ears gently back (rate per minute)	Supported as a potential measure of affect by Bornmann, Randle [6] and Ricci-Bonot and Mills [7]. Action defined by Wathan, Burrows [8].
Ears asymmetrical (rate per minute)	Supported as a potential measure of affect by Bornmann, Randle [6] and Lansade, Nowak [9]. Action defined by Wathan, Burrows [8].
Eye sclera (rate per minute)	Supported as a marker of negative affect by Lansade, Nowak [9] and Ricci-Bonot and Mills [7]. Action defined by Wathan, Burrows [8].
Eye half (rate per minute)	Supported as a marker of positive affect by Lansade, Nowak [9] and of changes in affect by Hintze and Schanz [10]. Action defined by Wathan, Burrows [8].
Eye bias left (rate per minute)	Supported as a potential measure of negative affect by Ricci-Bonot and Mills [7] and arousal with variable valence by Leliveld, Langbein [11] and Evans, Cameron-Whytock [12].

Frustration Acute conflict (rate per minute)	Supported as a potential measure of negative affect by Pannewitz and Loftus [3].
QBA relaxed (rate per minute)	Previously validated measures [13-16]. Potential measure of positive affect.
QBA at ease (rate per minute)	Previously validated measures [13-16]. Potential measure of positive affect.
QBA friendly (rate per minute)	Previously validated measures [13-16]. Potential measure of positive affect.
QBA uneasy (rate per minute)	Previously validated measures [13-15]. Potential measure of negative affect.
QBA alarmed (rate per minute)	Previously validated measures [13-15]. Potential measure of negative affect.

S3 Boxplots of individual measures

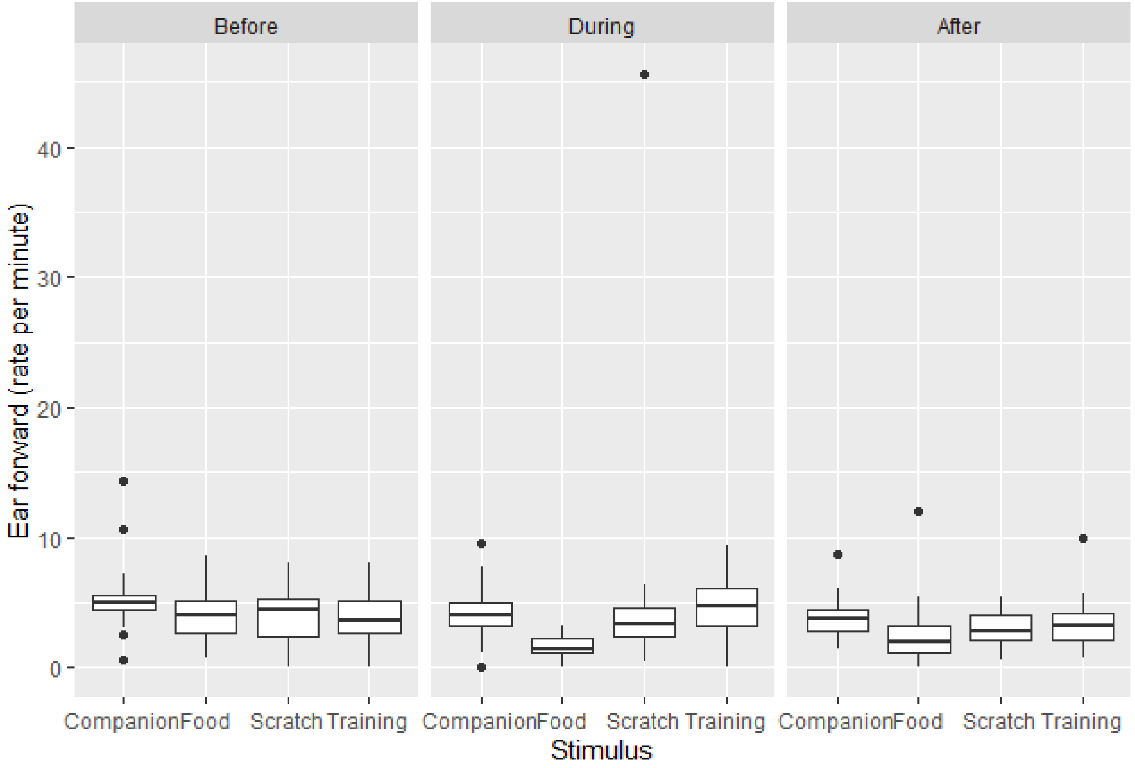

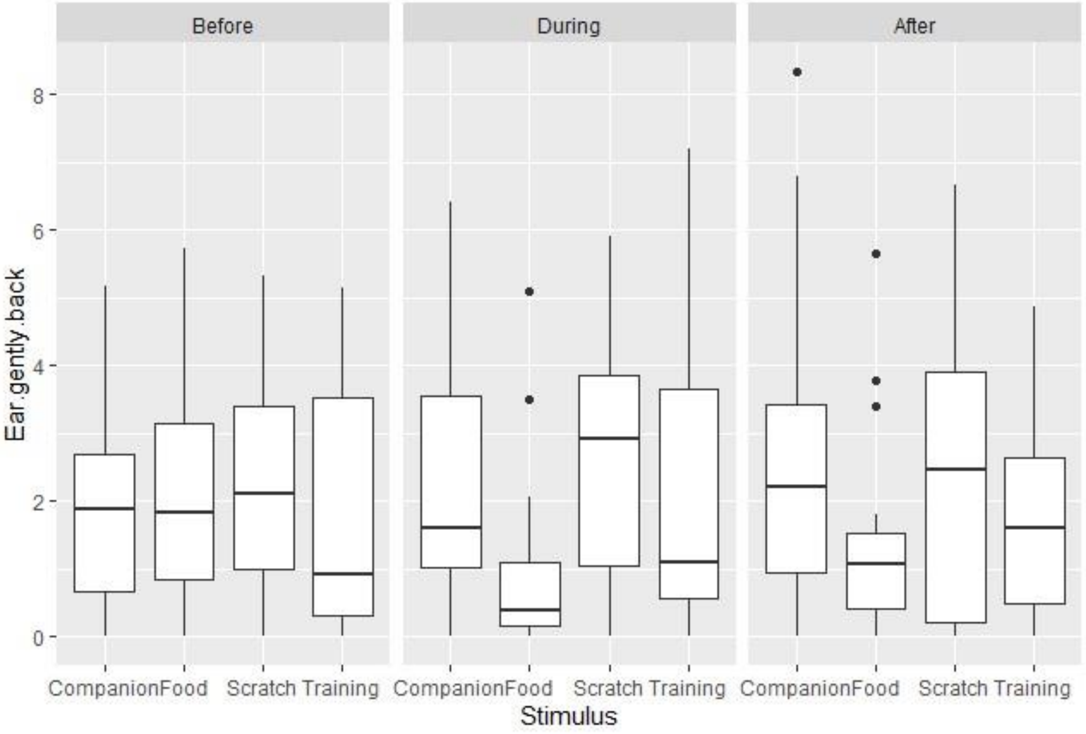

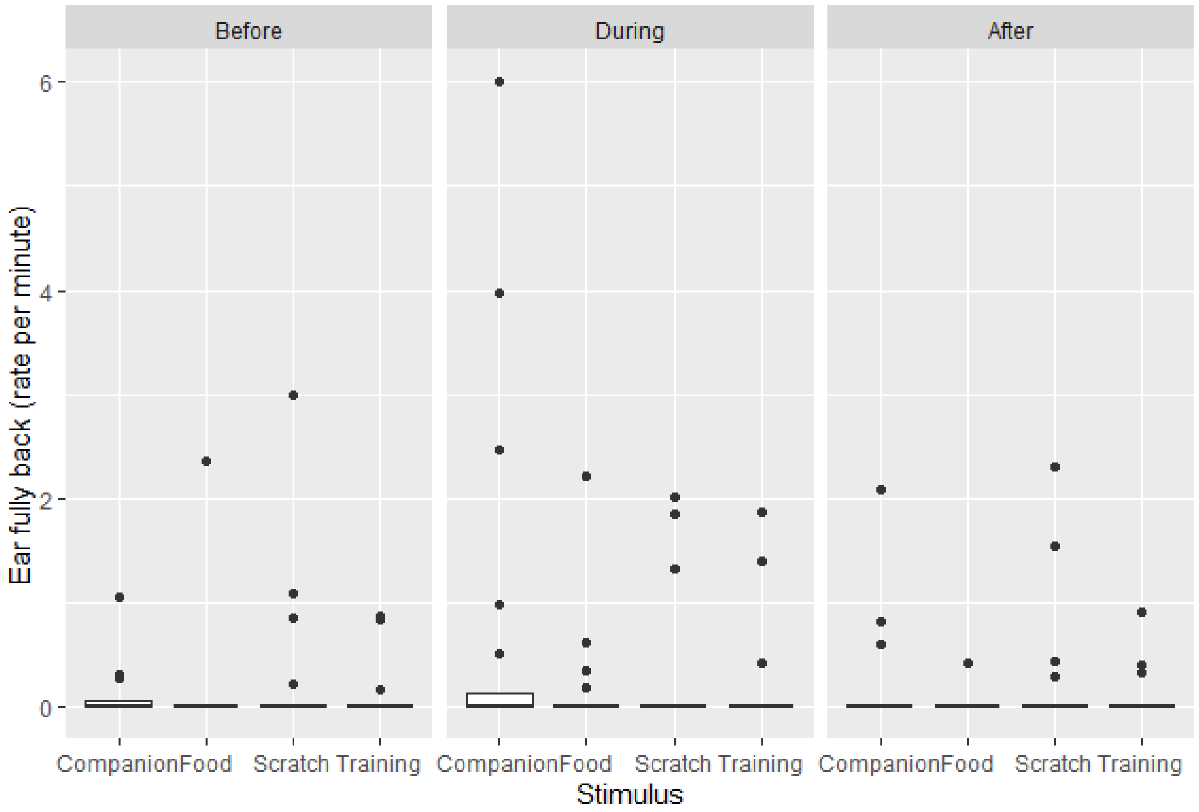
Before
During
After
Ear fully back (rate per minute)
6
4
2
0
CompanionFood
Scratch Training
CompanionFood
Scratch Training
CompanionFood
Scratch Training
Stimulus

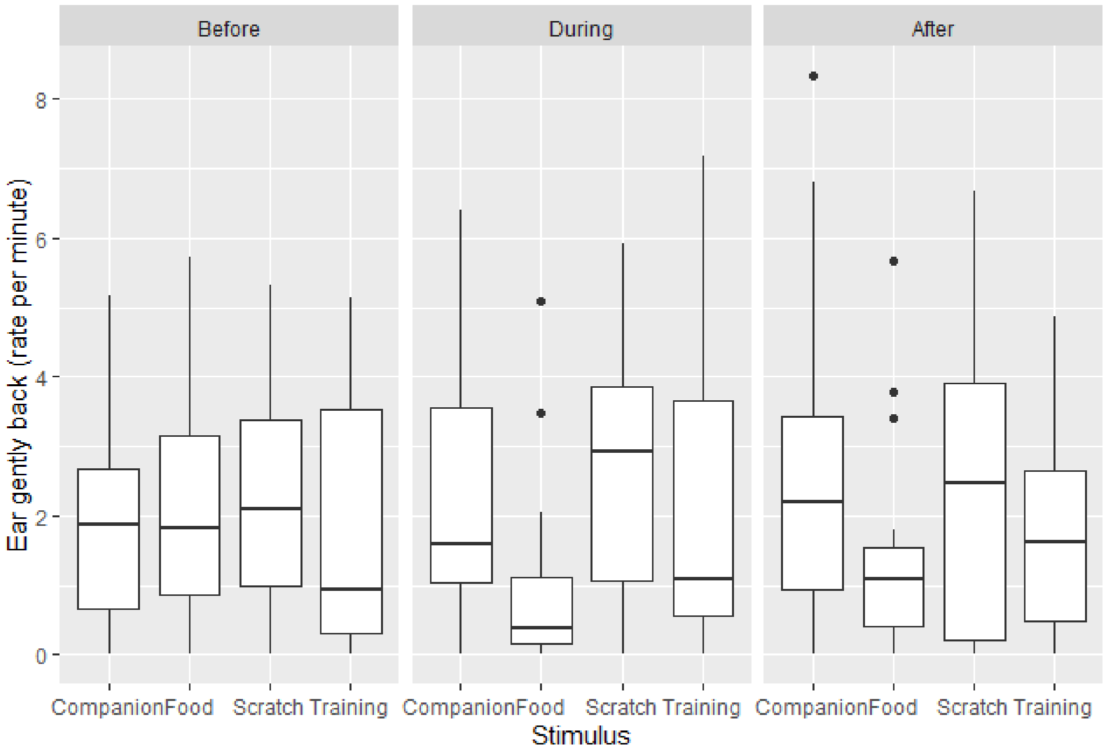
Before
During
After
Ear gently back (rate per minute)
8
6
4
2
0
CompanionFood
Scratch Training
CompanionFood
Scratch Training
CompanionFood
Scratch Training
Stimulus

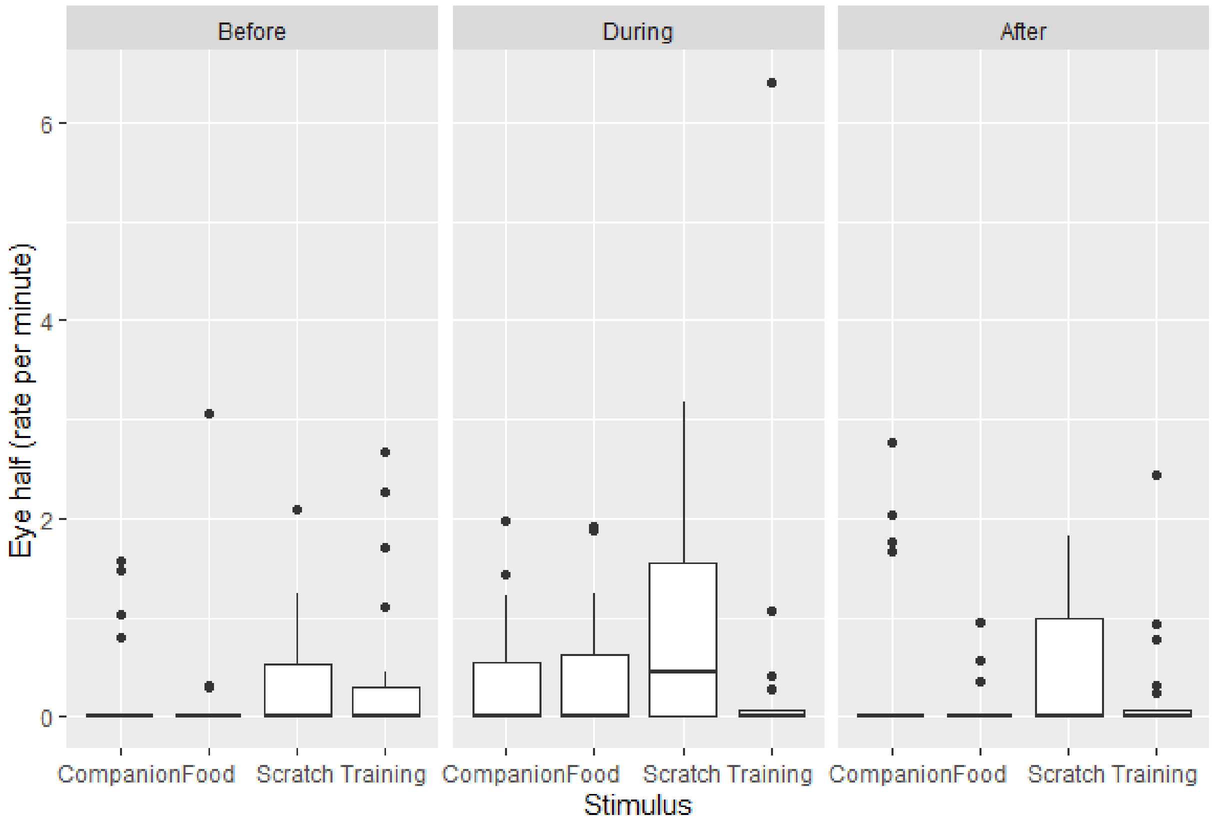
Before
During
After
Eye half (rate per minute)
0
2
4
6
CompanionFood
Scratch Training
Stimulus

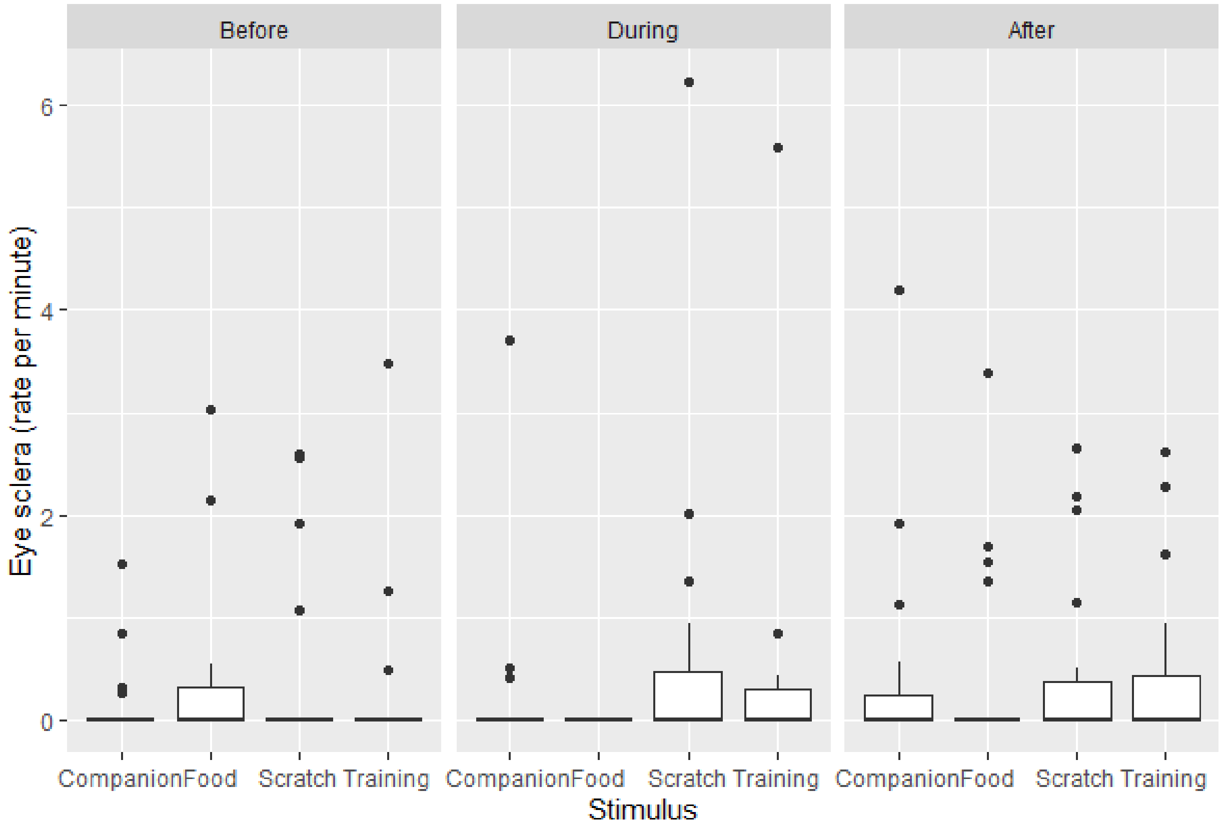
Before
During
After
Eye sclera (rate per minute)
0
2
4
6
CompanionFood
Scratch Training
Stimulus

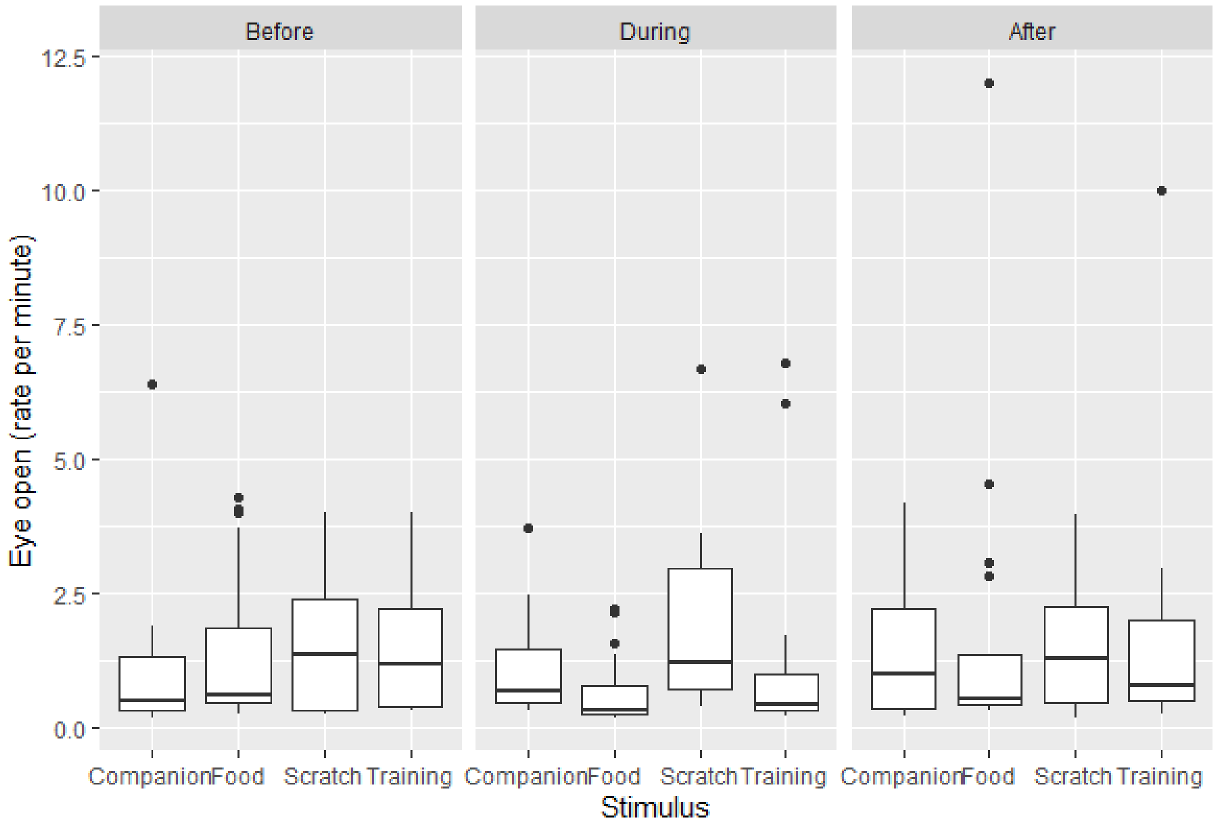
Before
During
After
12.5
10.0
7.5
5.0
2.5
0.0
Eye open (rate per minute)
CompanionFood
Scratch Training
Stimulus

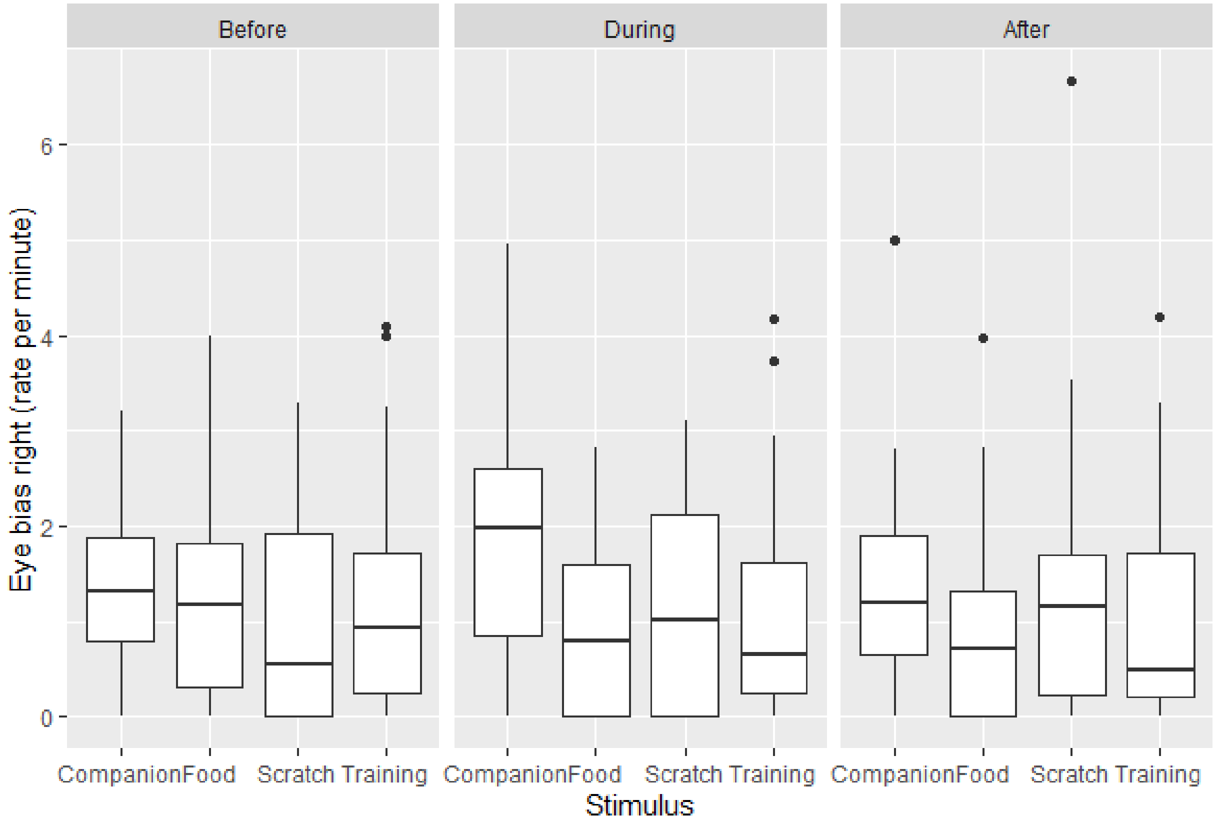
Before
During
After
6
4
2
0
Eye bias right (rate per minute)
CompanionFood
Scratch Training
Stimulus

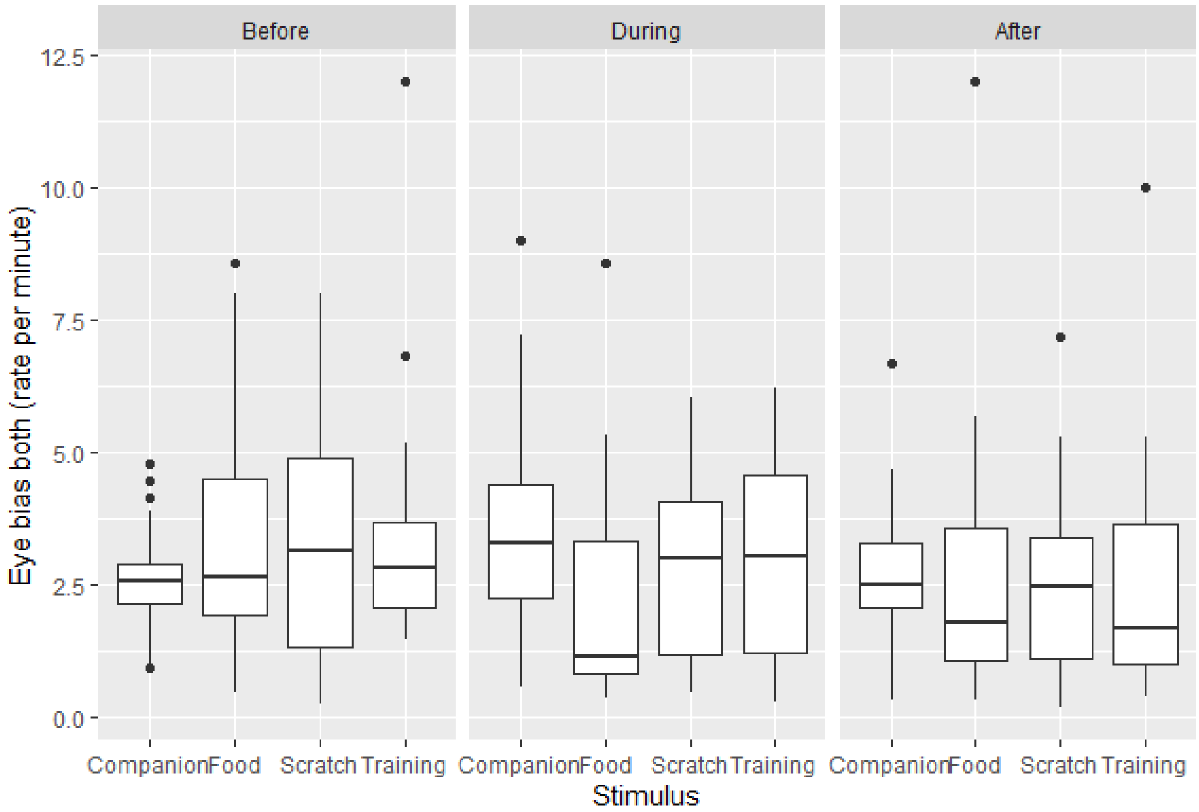
Before
During
After
Eye bias both (rate per minute)
12.5
10.0
7.5
5.0
2.5
0.0
CompanionFood
Scratch Training
Stimulus

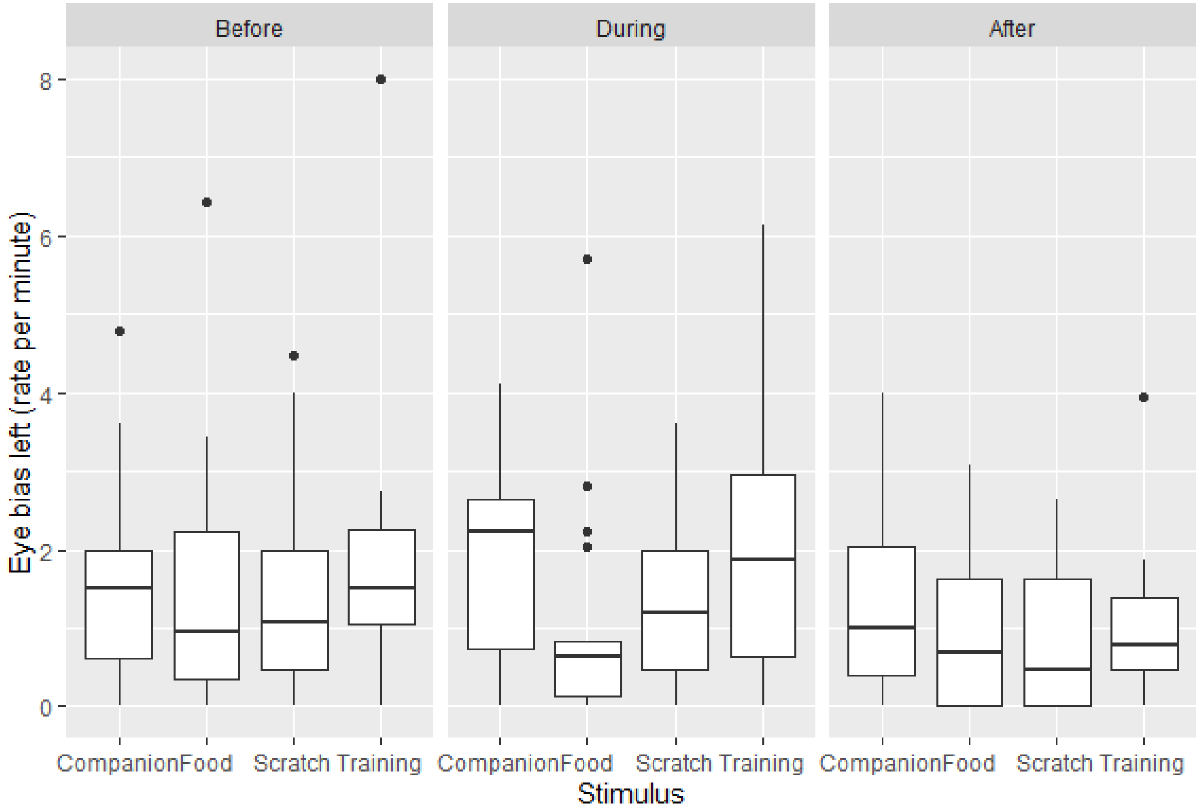
Before
During
After
Eye bias left (rate per minute)
8
6
4
2
0
CompanionFood
Scratch Training
Stimulus

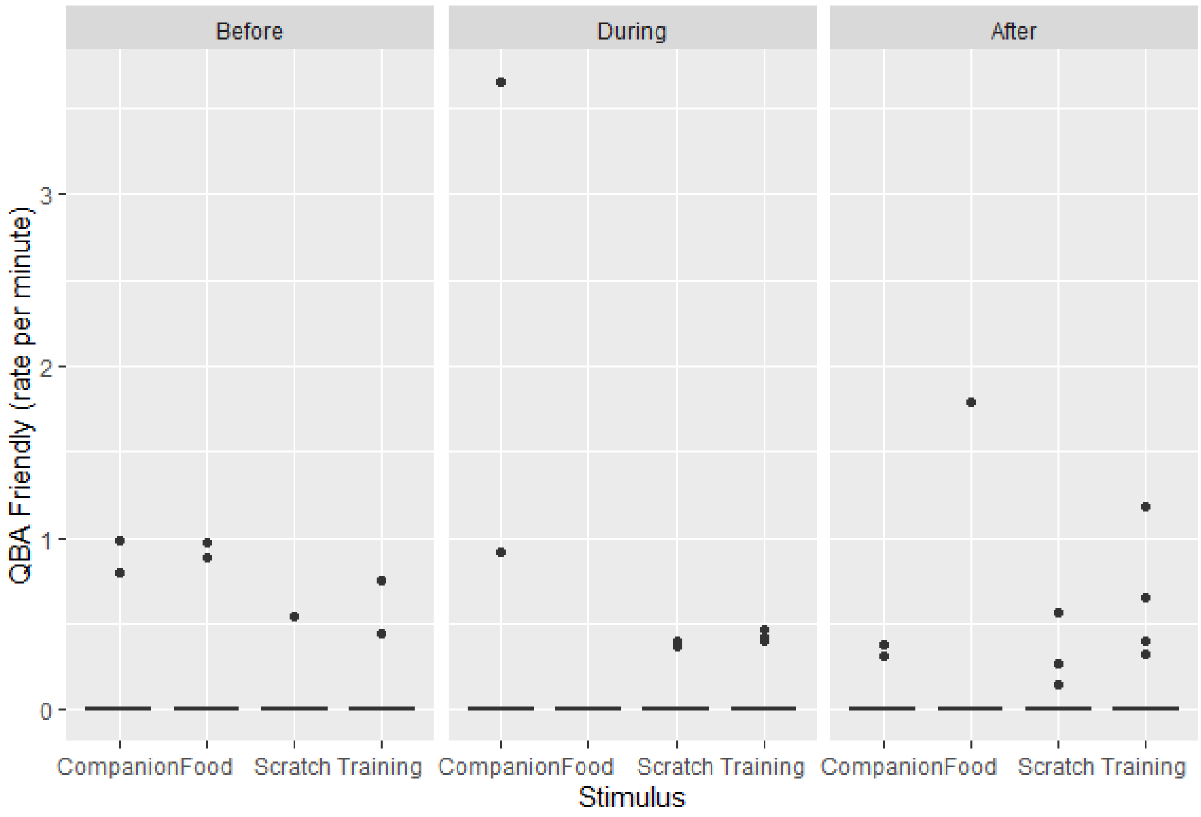

Before
During
After
QBA Friendly (rate per minute)
0
1
2
3
CompanionFood
Scratch Training
CompanionFood
Scratch Training
CompanionFood
Scratch Training
Stimulus

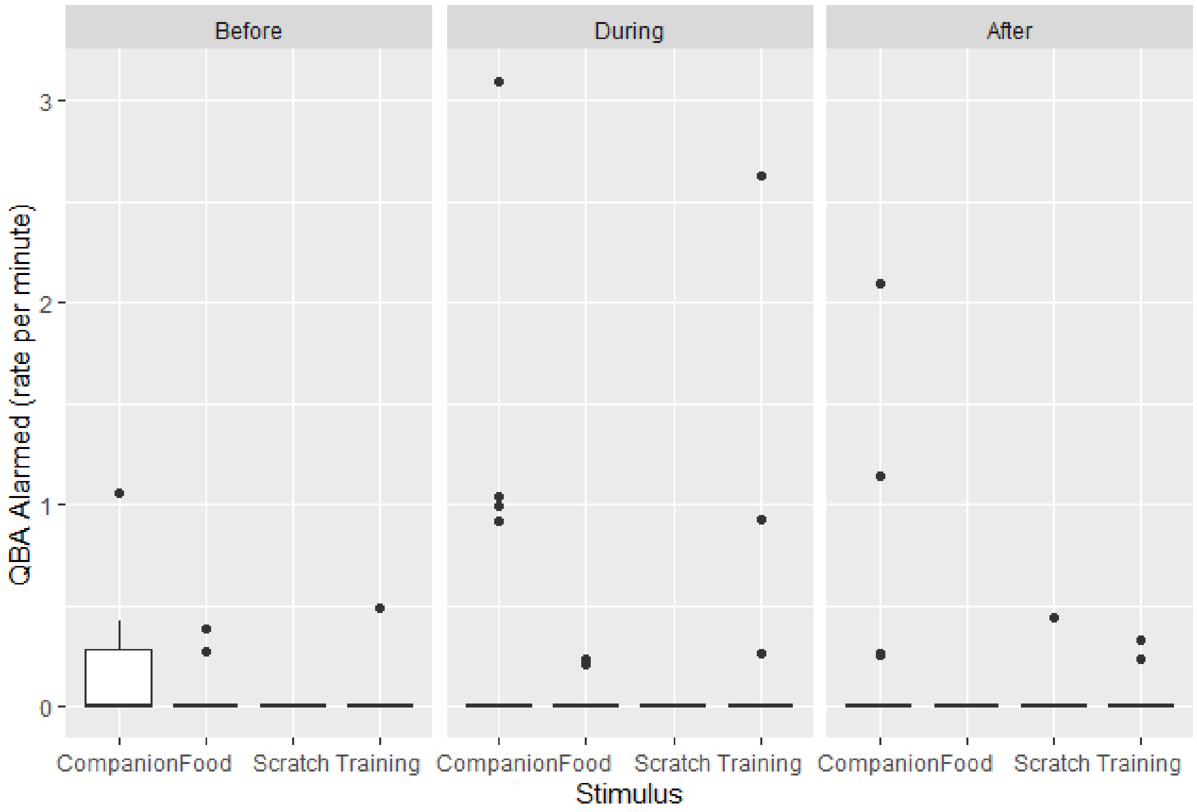

Before
During
After
QBA Alarmed (rate per minute)
0
1
2
3
CompanionFood
Scratch Training
CompanionFood
Scratch Training
CompanionFood
Scratch Training
Stimulus

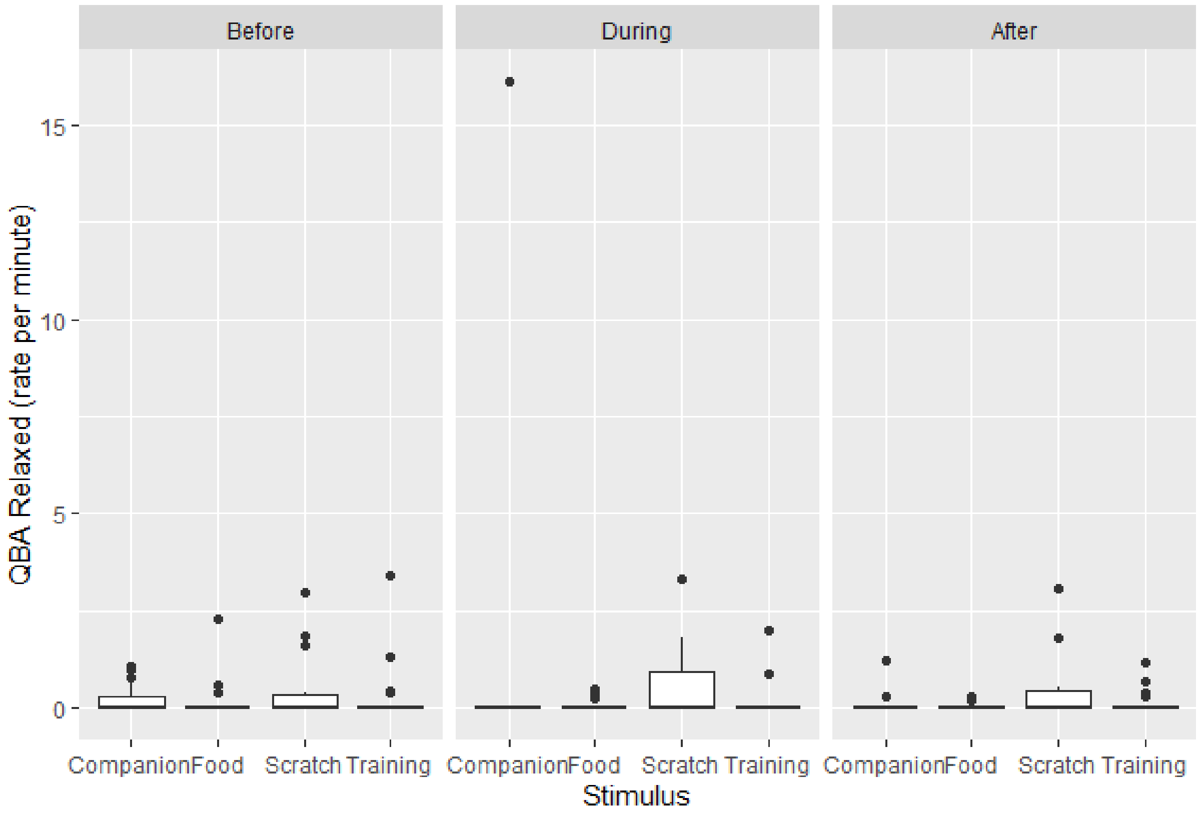
Before
During
After
QBA Relaxed (rate per minute)
15
10
5
0
CompanionFood
Scratch Training
Stimulus

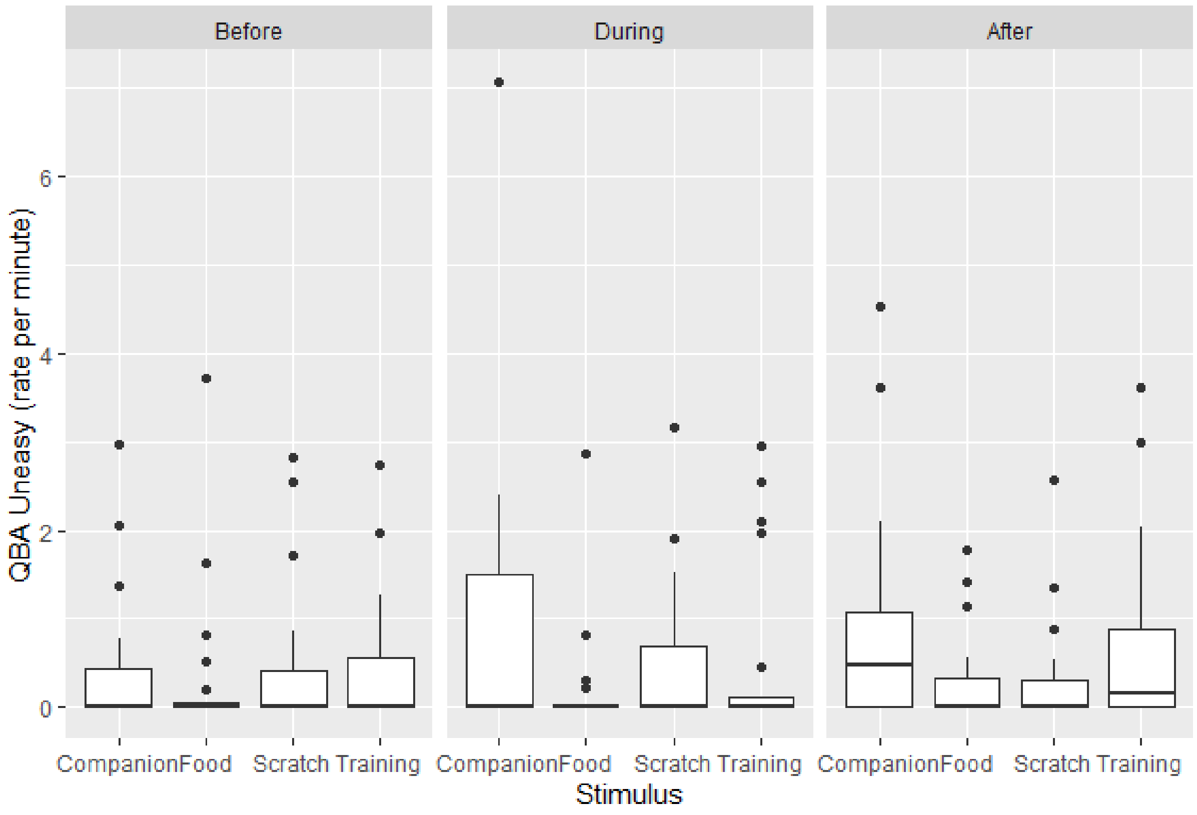
Before
During
After
QBA Uneasy (rate per minute)
6
4
2
0
CompanionFood
Scratch Training
Stimulus

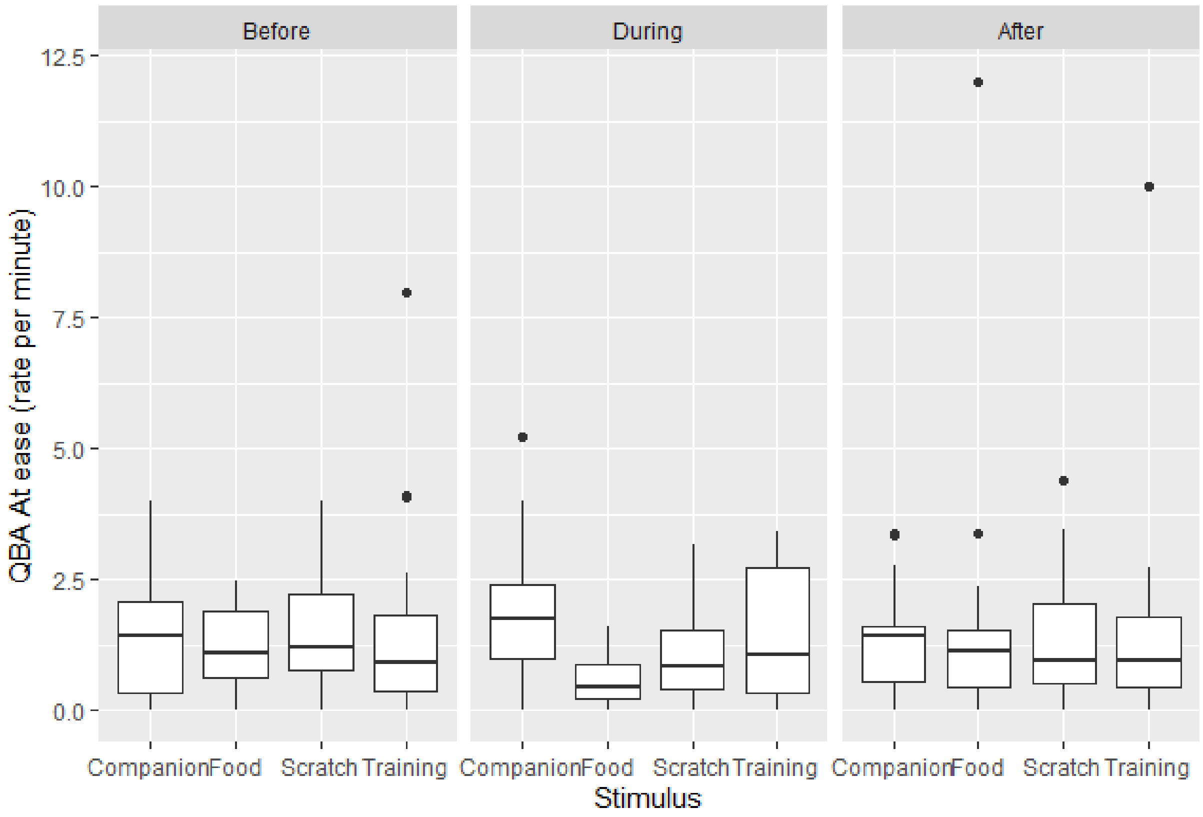
Before
During
After
QBA At ease (rate per minute)
12.5
10.0
7.5
5.0
2.5
0.0
Companion Food Scratch Training
Stimulus

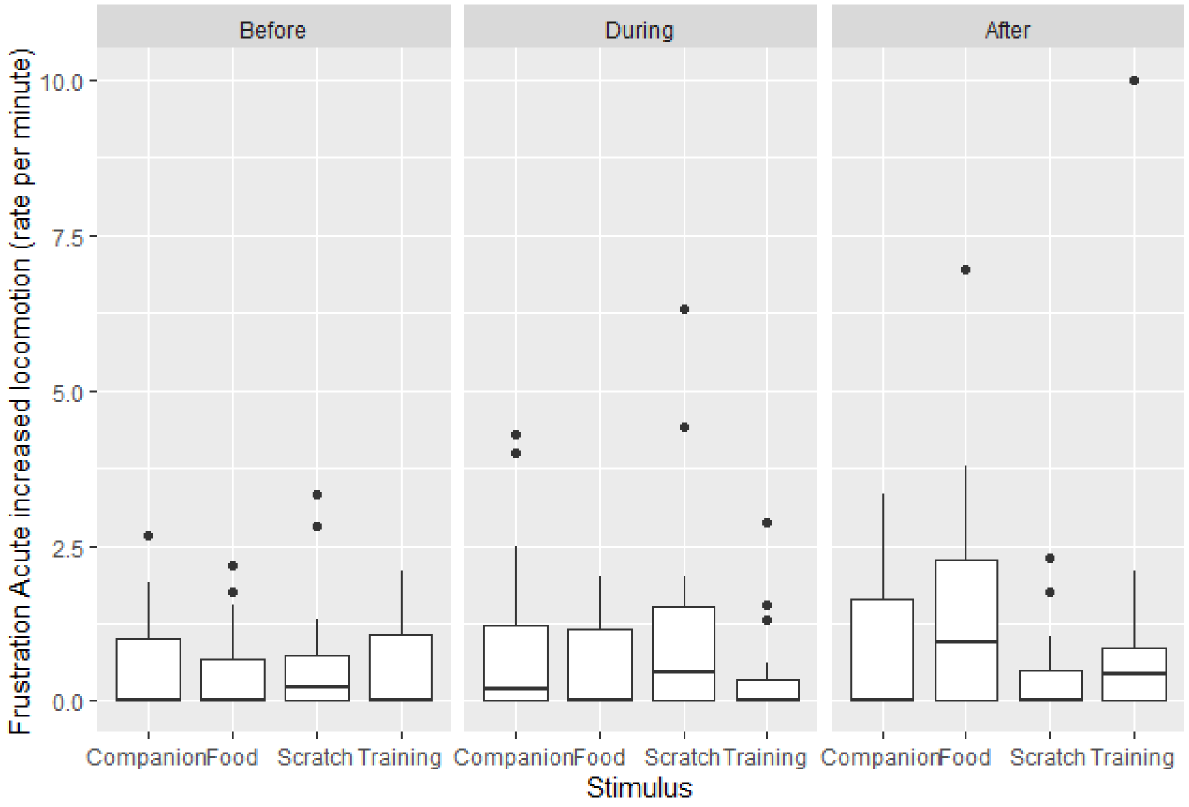
Before
During
After
Frustration Acute increased locomotion (rate per minute)
10.0
7.5
5.0
2.5
0.0
Companion Food Scratch Training
Stimulus

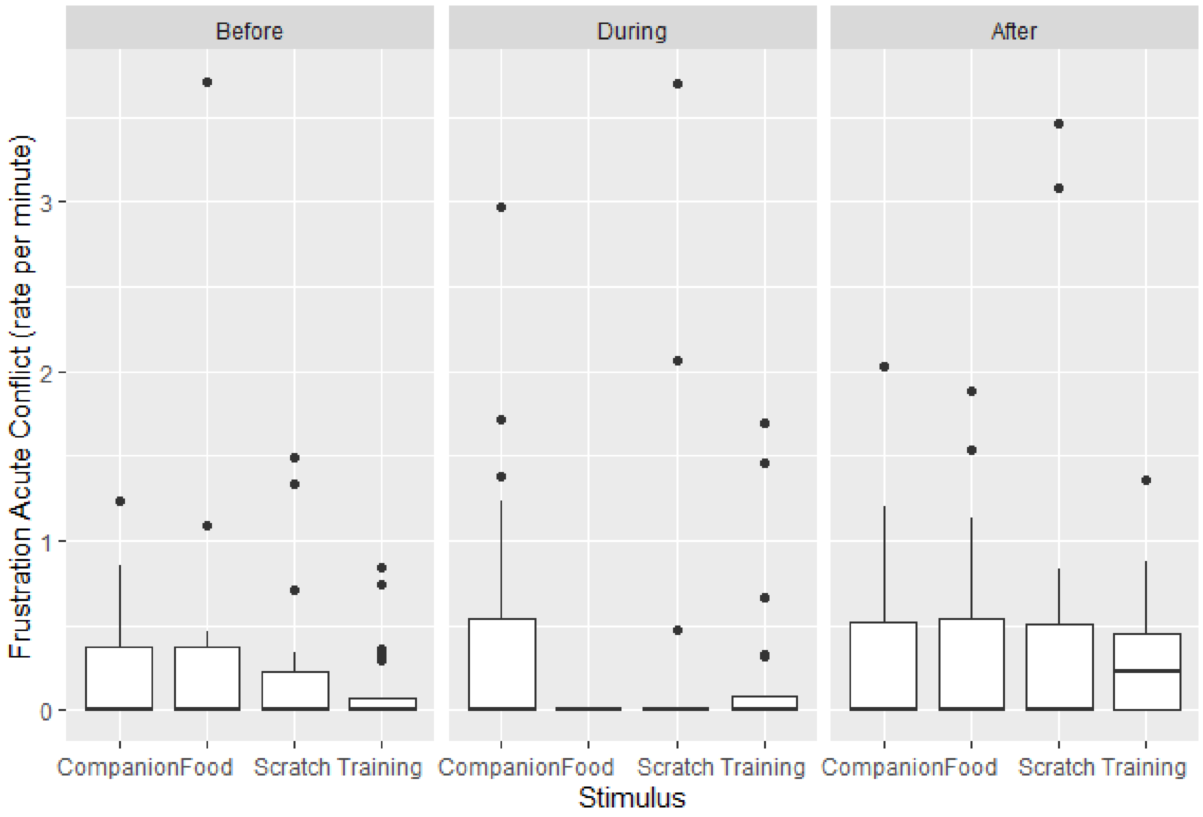
Before
During
After
Frustration Acute Conflict (rate per minute)
0
1
2
3
CompanionFood
Scratch Training
Stimulus

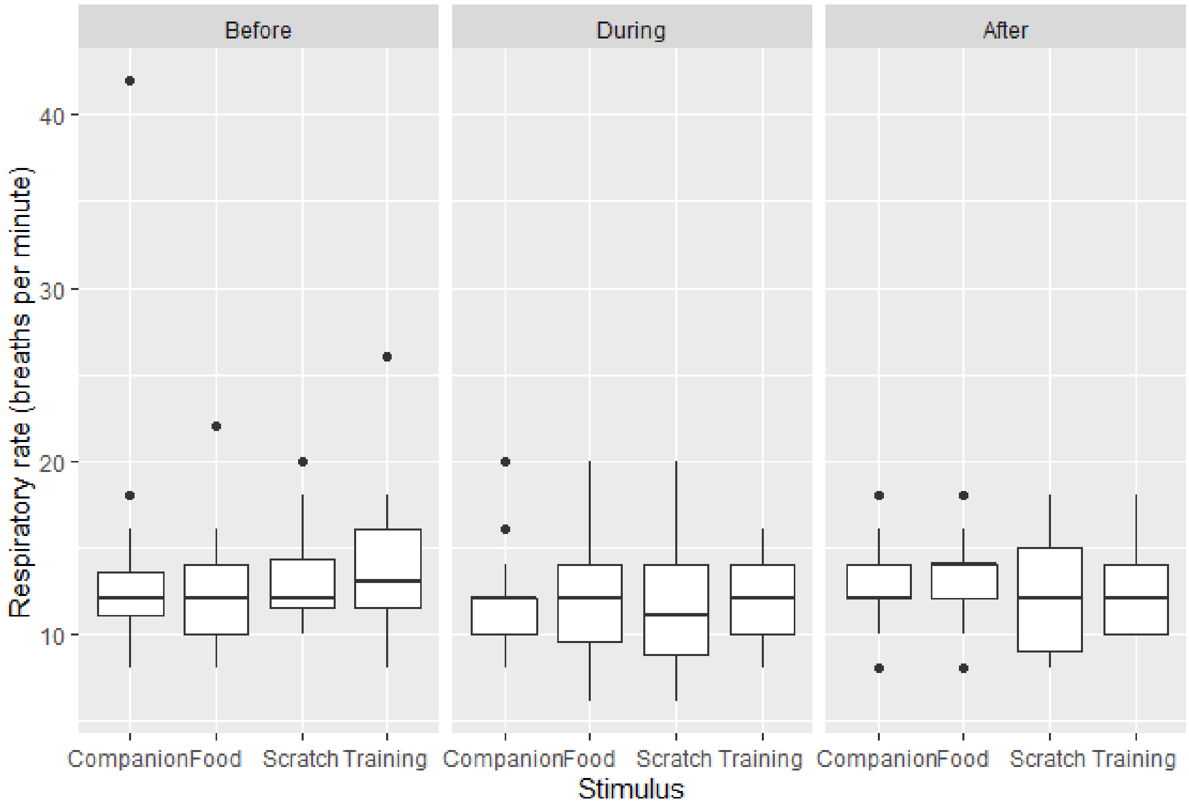
Before
During
After
Respiratory rate (breaths per minute)
10
20
30
40
CompanionFood
Scratch Training
Stimulus

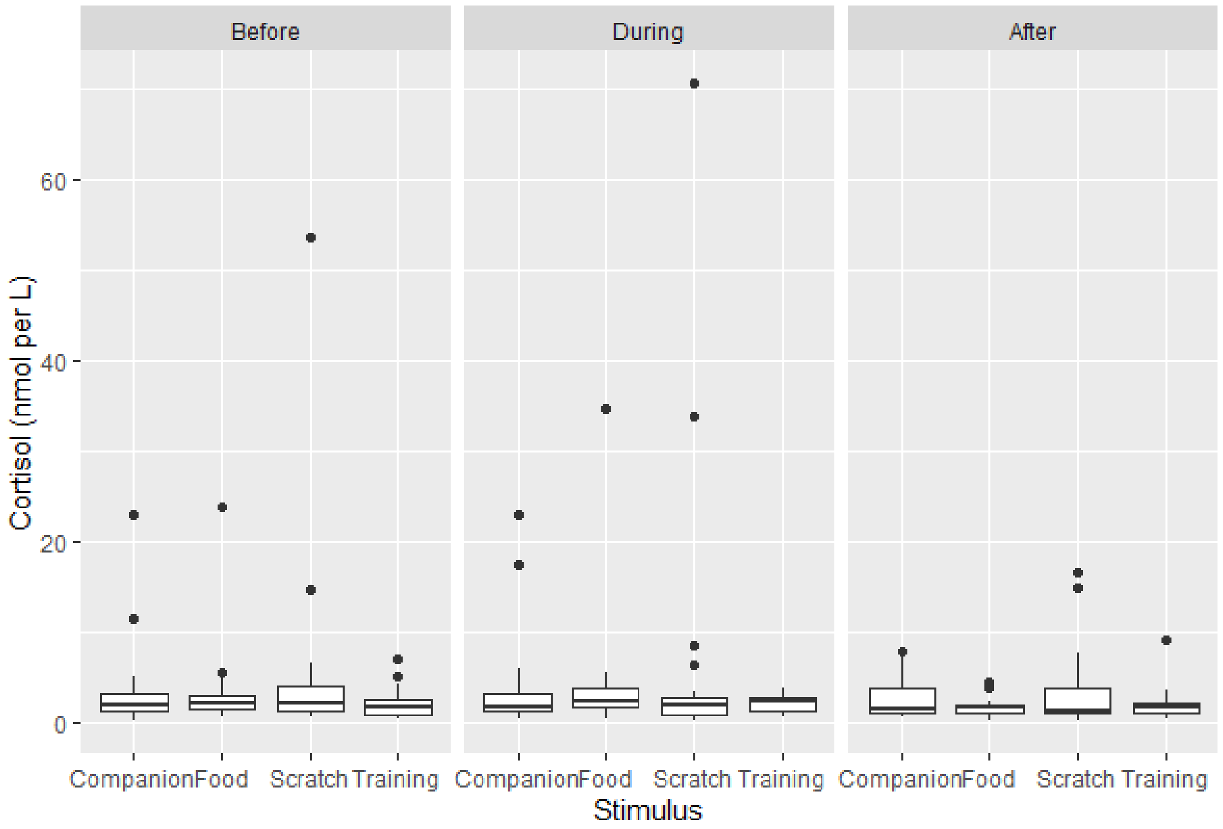
Before
During
After
Cortisol (nmol per L)
60
40
20
0
CompanionFood
Scratch Training
Stimulus

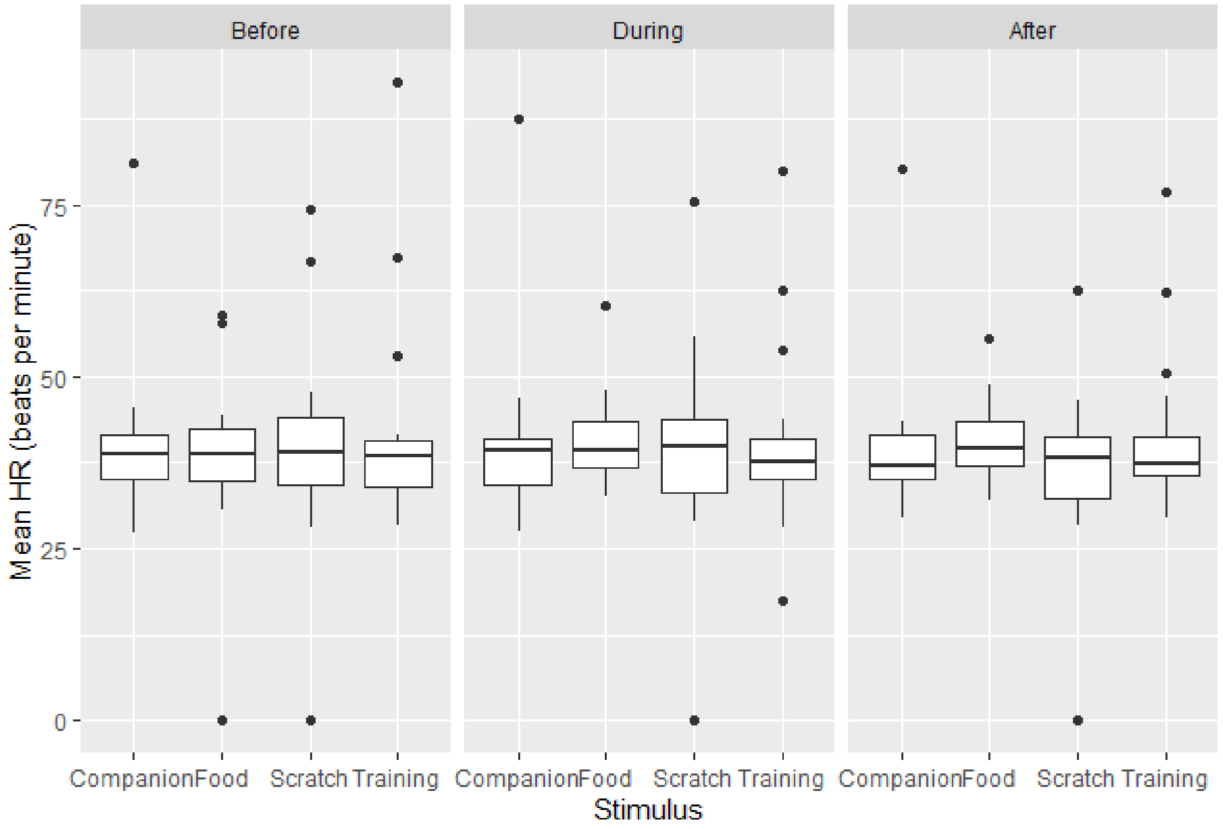
Before
During
After
Mean HR (beats per minute)
75
50
25
0
CompanionFood
Scratch Training
Stimulus

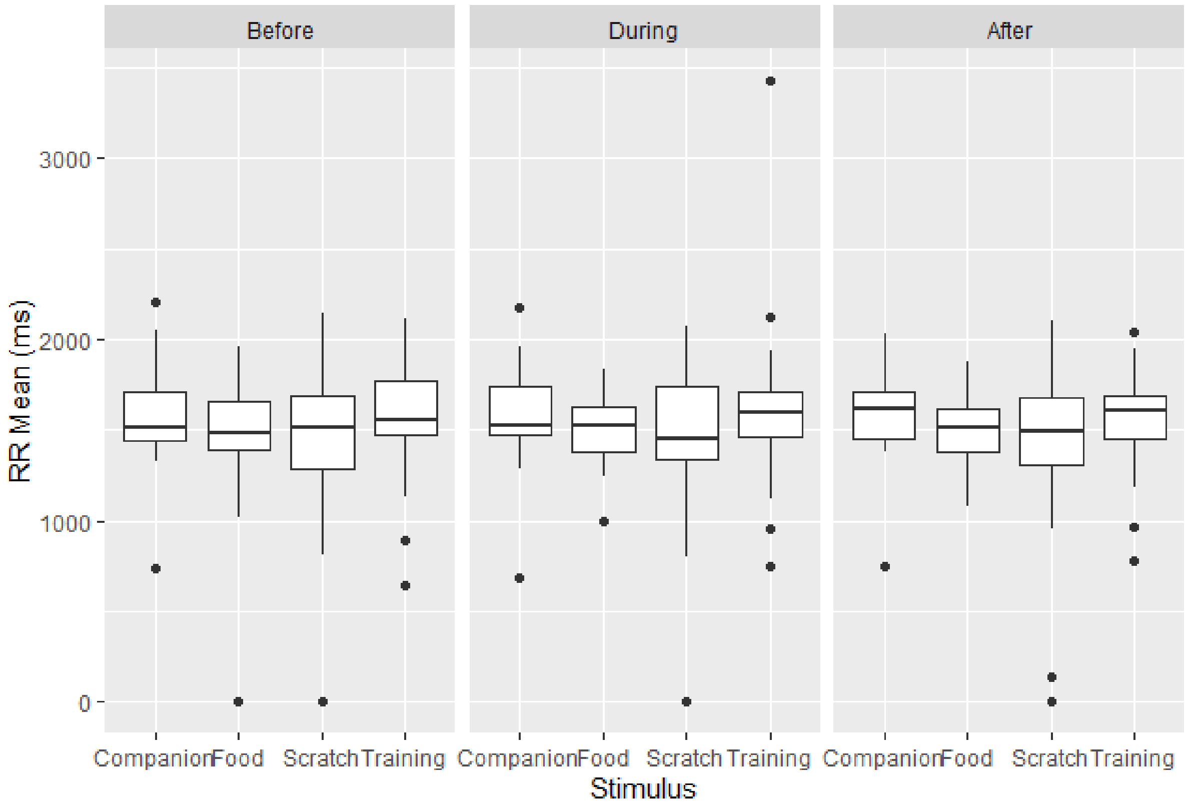
Before
During
After
RR Mean (ms)
3000
2000
1000
0
CompanionFood
Scratch Training
CompanionFood
Scratch Training
CompanionFood
Scratch Training
Stimulus

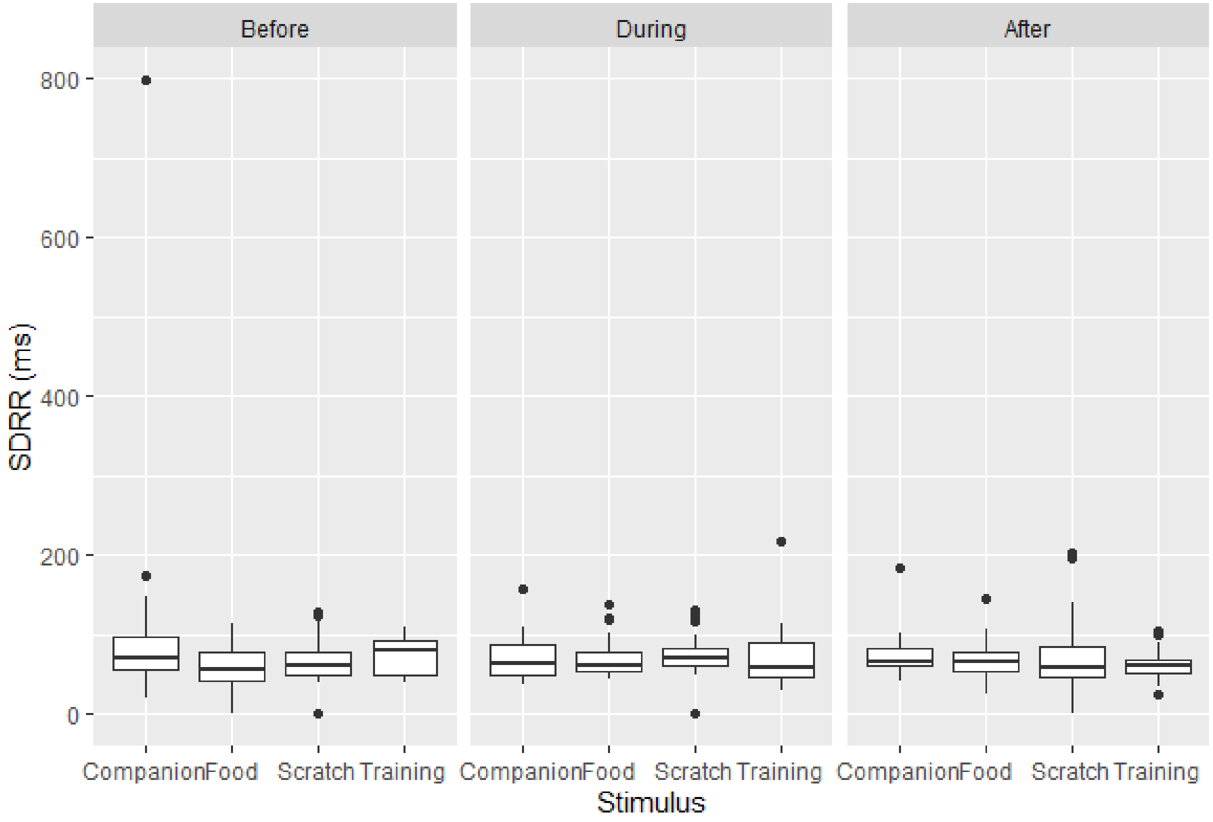
Before
During
After
SDRR (ms)
800
600
400
200
0
CompanionFood
Scratch Training
CompanionFood
Scratch Training
CompanionFood
Scratch Training
Stimulus

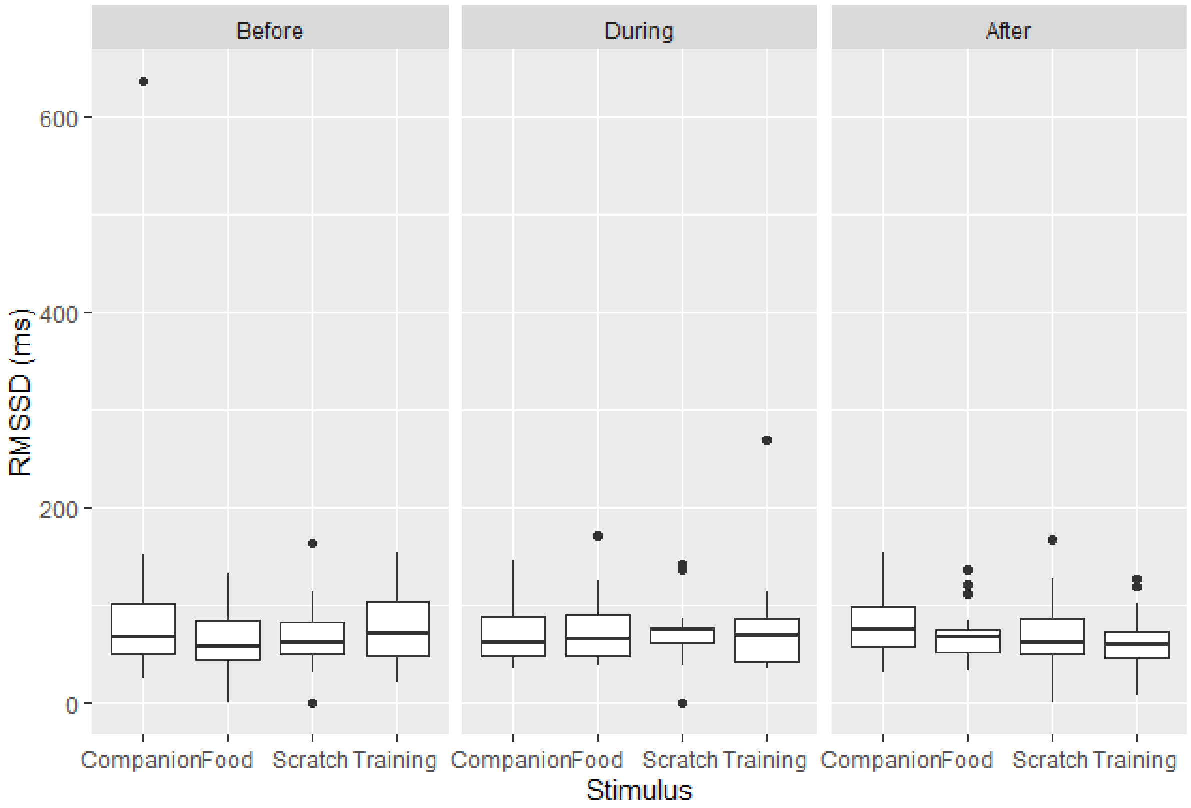
Before
During
After
RMSSD (ms)
0
200
400
600
CompanionFood
Scratch
Training
Stimulus

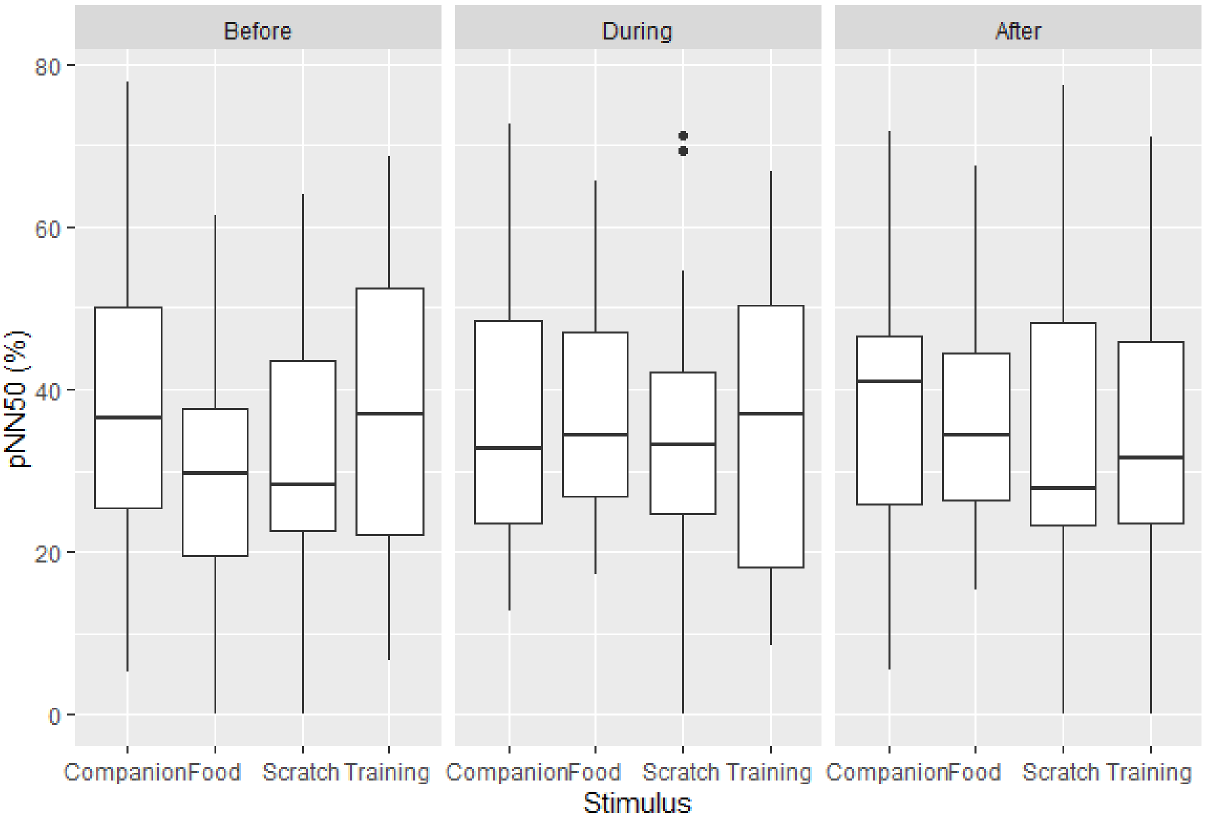
Before
During
After
pNN50 (%)
0
20
40
60
80
CompanionFood
Scratch
Training
Stimulus

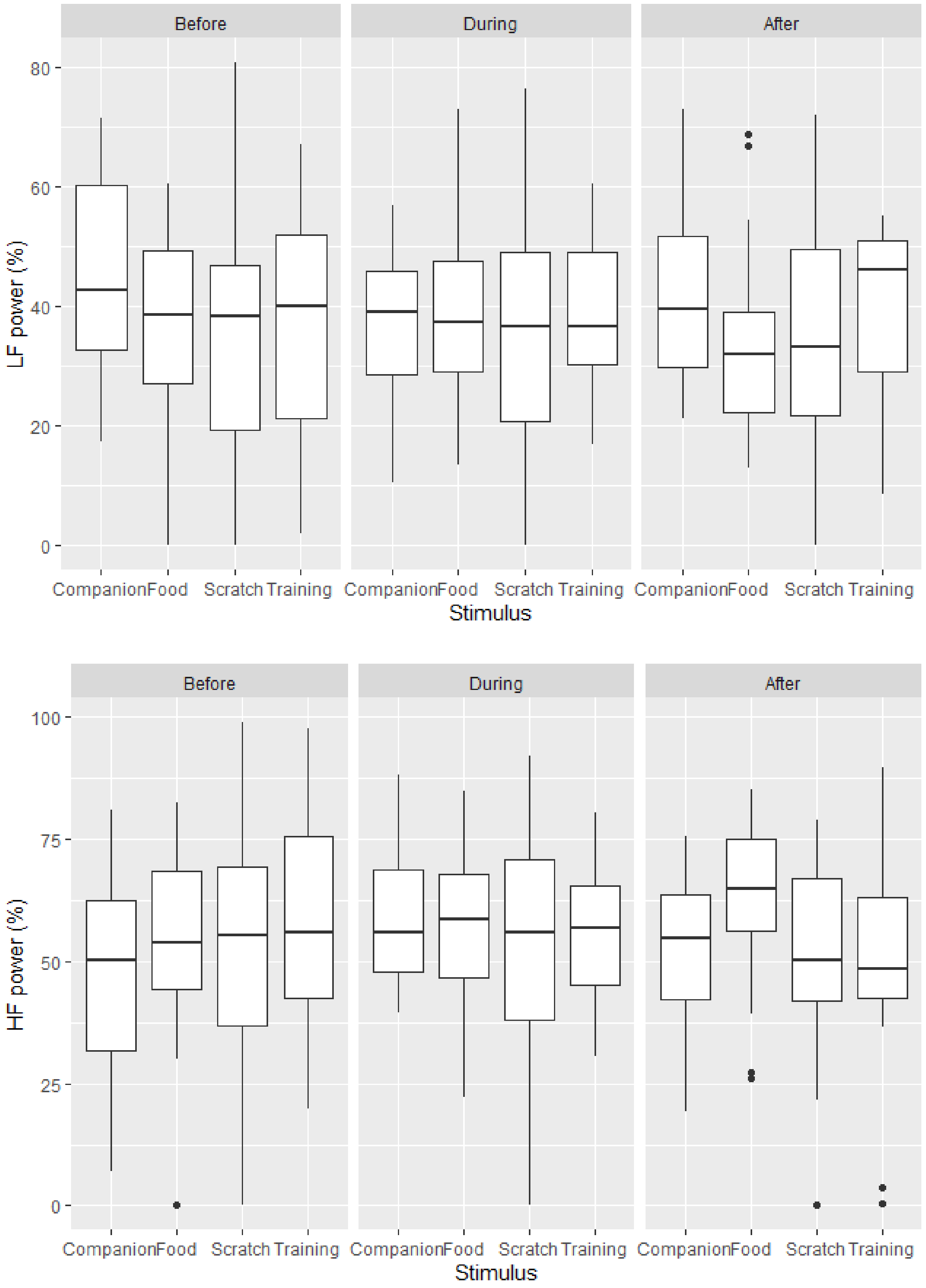
Before
During
After
LF power (%)
80
60
40
20
0
CompanionFood
Scratch Training
Stimulus
HF power (%)
100
75
50
25
0

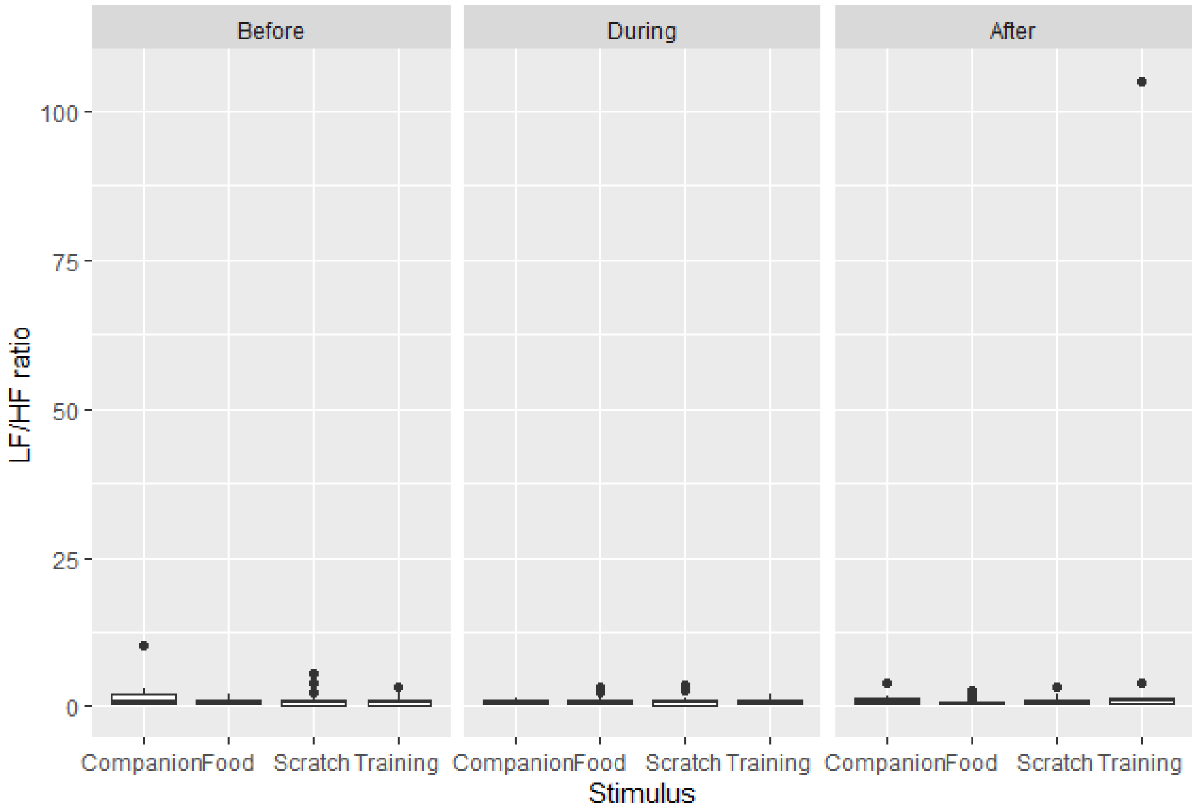

S4 Additional discussion of measures

Within quadrant one there are clusters of measures that are suggested within the literature to indicate a state of alertness such as 'LF power', indicative of sympathetic arousal [5] which appears statistically close to 'QBA alarmed' and 'mean heart rate', suggesting that these are likely to be measures of arousal rather than valence, a premise supported by Hall et al [17]. This is supported within quadrant one, where 'LF:HF ratio' is identified as having a positive loading on both PC's; and is identified within the literature as a measure to quantify the changing relationship between sympathetic and parasympathetic activities indicating the level of parasympathetic dominance [18], with a low 'LF:HF ratio' suggested by many to be indicative of high parasympathetic dominance [5, 19, 20]. However there is disagreement regarding this by others such as Shaffer and Ginsberg [21] and Billman [22] who suggest that this oversimplifies the complex non-linear interactions between sympathetic and parasympathetic divisions of the autonomic nervous system; with 'LF power' being particularly difficult to disentangle from its indications within parasympathetic drive, sympathetic drive and other unidentified factors. However, 'HF power' has been strongly linked to parasympathetic activity and is much less disputed [22] and within this study is shown within the loading plot to be appositional to 'LF power' and to 'LF:HF ratio'. This appears to indicate that, within these results, 'LF power' indicates a higher sympathetic state and that it is clustered with increased 'LF:HF ratio' within quadrant 1. Supporting this is the clustering of 'cortisol' within quadrant one, close to 'LF:HF ratio'. This cluster supports the denotation of quadrant one as an alert, potentially reward acquisition, appetitive state with the possibility that rises in cortisol level are resultant of increased arousal emanating from a state of anticipation and excitation. However, it is important to evaluate the fact that cortisol does not only represent a measure of stress, rather it plays important roles in blood sugar regulation, inflammation reduction, metabolism regulation and memory formation meaning that it is possible that rises in cortisol may represent other elements aside from a

stress response [23]. This is further supported when considering that cortisol was seen to increase across all induction stimuli which may have been the result of increased arousal or alternatively could be related to learning and memory processing [24].

‘RR mean’, the time between successive heartbeats (also termed inter-beat intervals, IBI), should be appositional to heart rate (as seen by its placement within quadrant 2), with increasing heart rate leading to shorter IBI’s; within this analysis ‘RR mean’ was directly appositional to ‘mean heart rate’ loaded negatively on PC1 (-0.32) and almost neutral on PC2 (0.02) indicating that a high ‘RR mean’ may suggest low arousal [21], potentially with positive valence depending on the other variables it is associated with. Supporting this the QBA variables ‘at ease’, ‘relaxed’ and ‘friendly’ identified within quadrant two as were the behavioural variables ‘ears side’, ‘eye half’ and ‘ear forward’. This cluster may indicate states of relaxation and contentment and as such could be proxy measures of RR mean and HF power, within positive valence.

Within quadrant three ‘pNN50’ and ‘RMSSD’ were closely clustered with negative loadings on both PCs 1 and 2. ‘pNN50’ is the percentage of successive RR intervals that differ by more than 50ms, ergo, it indicates high heart rate variability. Similarly, ‘RMSSD’ is the root mean square of successive RR interval differences therefore it is logical that these measures correlate closely. Within quadrant three these measures may indicate an association with low heart rate variability (these variables are lower in Q3), indicative of a shutdown, low motivation affective state. ‘SDRR’, the standard deviation of RR intervals, is also clustered close to both ‘RMSSD’ and ‘pNN50’ which would be expected given their similar properties [21]. All these variables sit with the time domain indices of HRV which quantify the amount of heart rate variability observed during the monitoring period [21]. ‘pNN50’ is closely correlated with parasympathetic activity alongside ‘RMSSD’ which is often the preferred metric by researchers [25], this is especially true to short-term HRV testing utilised within the design of this study [26]. Interestingly ‘SDRR’ and ‘pNN50’ are described within the literature as being more accurate measures of longer-term HRV over a 24-hour period therefore the finding that these measures are closely correlated within this study is interesting in terms of their prospective use over shorter measurement periods [21]. The only behavioural measure sitting within this quadrant is ‘ear asymmetrical’, loading neutrally onto PC1 (-0.007) and negatively on PC2 (-0.31) potentially indicating low levels of parasympathetic dominance (its placement is closer to the midline) in negative affect (strongly negatively loaded on the affect dimension), thus potentially a measure indicative of a rising level of arousal [27].

Quadrant four is suggested to be representative of negative affective state in high arousal. Within this quadrant several previously identified behavioural markers of negative affect are closely clustered including ‘ear fully back’ [9], ‘eye sclera’ [9], and ‘QBA uneasy’ [14]. Also clustered close to these measures are the eye bias measures ‘right’ and ‘both’ with each measure being highly positively loaded on PC1 (0.25 and 0.20 respectively) and negatively loaded on PC2 (-0.11 for both measures) which could be considered as potential measures of negative valence in a highly aroused context. However, these variables are located relatively close to ‘eye bias left’ which also loads highly on the arousal dimension potentially signifying that eye bias may be indicative of arousal more generally, rather than specific to valence or that it may be specific within individuals leading to a variety of responses across this cohort as indicated previously by Leliveld et al [11]. The ‘respiratory rate’ variable sits just within this quadrant, being moderately positively loaded on PC1 (0.10) and just negatively loaded on PC2 (-0.07); although this variable is along the same plane within PC1 as ‘eye

bias left' and 'mean heart rate' with no visual relationship being identified when examining these variables in a paired manner. 'Respiratory rate' did not vary widely during the different stimuli or trials phases and as such may be a less nuanced measure of the valence of affective state and of subtle increases in arousal.

Behavioural variables 'QBA uneasy', 'frustration acute locomotion', 'frustration acute conflict' and 'ears gently back' were relatively closely clustered within the middle of quadrant four. Logically, behaviours indicative of uncertainty such as conflict, increased movement and general uneasiness would be expected to be closely aligned [28] and of a lower level of arousal than more overt signs such as 'ears fully back' and 'eye sclera' that are loaded more significantly on PC1. 'Ears gently back' was not anticipated to be identified within this quadrant as prior research has indicated that this ear position, with the ears roughly in line with the nostrils, is indicative of positive emotions in horses [27]. Within this cohort this does not appear to be supported however it is clear is that 'ear gently back' is indicated as a variable in lower arousal than 'ears fully back' and potentially of greater negative valence, perhaps indicative of general uneasiness given its relative statistical proximity to 'QBA uneasy'. Interestingly 'ear gently back' increased markedly during wither scratching as has been found previously [9, 29] which may support its use as measure more attributable to quadrant 2; 'ear gently back' frequency rate varied hugely across the other three induction stimuli which could account for its ambiguous placement within the PCA analysis.

S5 Pre-trial questionnaire and subsequent short form personality scoring

Measuring Positive Affective State in Equines: Pre-trial equine questionnaire

This form is used to collect relevant information regarding your equine prior to the trial sessions. The data provided will allow for careful analysis of the results while considering the individual situation of each equine and their personality facets. Please do complete the questionnaire as fully and accurately as possible. We are very grateful for your participation in this project which will help horse owners and professionals to understand how happy their equine is.

The project has full ethical approval from Newcastle University's Animal Welfare Ethical Review Board (AWERB project ID: 1025). If you have any questions about the study please email the lead researcher Loni Loftus at i.l.loftus@newcastle.ac.uk

Please read the study information document which gives you an overview of the purpose of the study and the methods and processes we will be using over the two data collection days spent with you and your equine. Following this, please check the 'I consent' box below to consent to your equine's data being collected and used in the study.

Please state your full name *

Your answer

Please provide a contact phone number

Your answer

Please provide a contact email address *

Your answer

I consent to my equine's data being collected and used within the study. *
I understand that once data are collected they will be anonymised and therefore I will be unable to withdraw the data from the study.
I understand and consent to images of my equine potentially being used within publications and presentations relevant to the study.

◯ I consent

Equine information

This section collects demographic information on your equine and their environment.

Equine name *

Your answer

Equine age in years (in number digits) *

Your answer

Equine breed *

Your answer

Sex *

- ○ Mare
- ○ Gelding
- ○ Stallion

Equine activities (select all that apply) *

- ☐ Non-ridden
- ☐ Elite competition (such as SJ, BD, BE, Racing, Driving)
- ☐ Affiliated competition
- ☐ Unaffiliated competition
- ☐ Hacking /pleasure riding
- ☐ Breeding
- ☐ Education (such as riding school, equine assisted therapy)
- ☐ Other:

How long has this equine been in your ownership or care? *

Choose

Has anything changed in relation to your equines environment (location, other horses, routine etc) in the last 6 weeks? *

- Yes
- No

If you answered yes to the question above regarding your equine's environment, please state how this has changed below.

Your answer

Is your equine healthy to the best of your knowledge? *

- Yes
- No
- Unsure

If you stated No or Unsure regarding your equines general health please give further details of medical conditions here.

Your answer

Does your equine have any dietary intolerances or are they on a specialised diet for health purposes? *

- Yes
- No

If you answered Yes to dietary issues/specialised diet please give further details here

Your answer

Does your equine have another equine friend at their location? *Please note that access to another friendly horse is required for this research. *

◯ Yes

◯ No

Has your horse ever experienced target training? *

◯ Yes

◯ No

◯ Unsure

Do you think that your horse enjoys scratches (such as wither scratches)? *

◯ Yes

◯ No

How do you reward your horse for correct behaviours? Please select all that apply. *

- [ ] Food treats
- [ ] Verbal praise (e.g. good boy/girl)
- [ ] Scratches
- [ ] Pressure release
- [ ] Patting
- [ ] Stroking
- [ ] Head rubbing
- [ ] No reward

Is your equine used to wearing a girth, surcingle or roller? *Please note that this trial requires the horse to wear a Polar equine heart rate monitor band. *

- ○ Yes
- ○ No

Will your horse be comfortable with having salivary cortisol swabs taken? This is required for the trial. Three swabs per test will be taken (12 in total across the four tests) with a small cotton collecting stick inserted into the mouth at the corner where the bit usually sits. *

- ○ Yes
- ○ No

Does your horse have any behavioural issues? Please select all that apply. *

- [ ] Repetitive behaviours (such as crib biting, wind sucking, box walking, weaving, fence pacing)
- [ ] Self mutilation
- [ ] Aggression towards humans (bite, kick behaviours)
- [ ] Aggression towards other equines (bite, kick behaviours)
- [ ] Aggression towards other species (dogs, sheep etc)
- [ ] Fears or phobias (such as aversion to noises, headshyness etc)
- [ ] Handling issues (such as barging, pulling away from the handler etc)
- [ ] Ridden issues (bucking, rearing, bolting)
- [ ] Resource guarding (for example food)
- [ ] None

Is your horse comfortable being tied up? *

- ○ Yes, totally comfortable
- ○ For short periods of time
- ○ No

Short form of the Equine Personality Eight-factor Model (EPEM-A)

Equine personality group assessments can be made with this questionnaire by assigning a numerical score for all the personality groups listed on the following pages. Make your judgments based on your own understanding of the group guided by the list of included traits. The equine's own behaviours and interactions with other equines should be the basis for your numerical ratings. Use your own subjective judgment of typical equine behaviour to decide if the equine you are scoring is above, below, or average for a group. The following seven-point scale should be used to make your ratings.

Scoring system

1	2	3	4	5	6	7
Least or negligible amount of the trait	Low amount of the trait	Less than average amount of the trait	Average amount of the trait	Slightly higher than average amount of the trait	High amount of the trait	Very high amount of the trait

Please give a rating for each group even if your judgment seems to be based on a purely subjective impression of the equine and you are somewhat unsure about it. Indicate your rating by placing a cross in the box underneath the chosen number as shown, X

EXUBERANT: Includes Excitable, Playful, Active, Distractible, Impulsive.

Least	1	2	3	4	5	6	7	Most

NERVOUS: Includes Cautious, Fearful, Acquiescent, Vulnerable, Sensitive, Timid.

Least	1	2	3	4	5	6	7	Most

UNRESPONSIVE: Includes Depressed, Unemotional, Solitary, Unperceptive.

Least	1	2	3	4	5	6	7	Most

STEADY: Includes Predictable, Conventional, Cool.

Least	1	2	3	4	5	6	7	Most

BOLD: Includes Assertive, Independent, Decisive, Persistent, Individualistic.

Least	1	2	3	4	5	6	7	Most

AFFILIATIVE: Includes Friendly, Helpful, Sociable, Sympathetic, Gentle, Affectionate.

Least	1	2	3	4	5	6	7	Most

UNFRIENDLY: Includes Irritable, Defiant, Bullying, Confrontational.

Least	1	2	3	4	5	6	7	Most

INVESTIGATORY: Includes Inventive, Innovative, Curious.

Least	1	2	3	4	5	6	7	Most

Equine Personality Eight-Factor Model (EPEM)

Equine personality assessments can be made with this questionnaire by assigning a numerical score for all the personality traits listed on the following pages. Make your judgments based on your own understanding of the trait guided by the short clarifying definition following each trait. The equine's own behaviours and interactions with other equines should be the basis for your numerical ratings. Use your own subjective judgment of typical equine behaviour to decide if the equine you are scoring is above, below, or average for a trait. The following seven-point scale should be used to make your ratings.

Scoring system

1	2	3	4	5	6	7
Least or negligible amount of the trait	Low amount of the trait	Less than average amount of the trait	Average amount of the trait	Slightly higher than average amount of the trait	High amount of the trait	Very high amount of the trait

Please give a rating for each trait even if your judgment seems to be based on a purely subjective impression of the equine and you are somewhat unsure about it. Indicate your rating by placing a cross in the box underneath the chosen number as shown, | X |

DISTRACTIBLE: Equine is easily distracted and has a short attention span.

Least	1	2	3		4	5	6	7	Most

PLAYFUL: Equine is eager to engage in lively, vigorous, sportive, or acrobatic behaviours with or without other horses.

Least	1	2	3	4	5	6	7	Most

ACTIVE: Equine spends little time idle and seems motivated to spend considerable time either moving around or engaging in some overt, energetic behaviour.

Least	1	2	3	4	5	6	7	Most

EXCITABLE: Equine is easily aroused to an emotional state. Equine becomes highly aroused by situations that would cause less arousal in most horses.

Least	1	2	3	4	5	6	7	Most

IMPULSIVE: Equine often displays some spontaneous or sudden behaviour that could not have been anticipated. There often seems to be some emotional reason behind the sudden behaviour.

Least	1	2	3	4	5	6	7	Most

FEARFUL: Equine reacts excessively to real or imagined threats by displaying behaviours such as snorting, shying, moving away or other signs of anxiety or distress.

Least	1	2	3	4	5	6	7	Most

CAUTIOUS: Equine often seems attentive to possible harm or danger from its actions. Equine avoids risky behaviours.

Least	1	2	3	4	5	6	7	Most

TIMID: Equine lacks self-confidence, is easily alarmed and is hesitant to venture into new social or non-social situations.

Least	1	2	3	4	5	6	7	Most

VULNERABLE: Equine is prone to be physically or emotionally hurt as a result of highly assertive behaviour, aggression, or attack by another horse.

Least	1	2	3	4	5	6	7	Most

ACQUIESCENT: Equine often gives in or yields to another horse. Equine acts as if it is subordinate or defers to other horses.

Least	1	2	3	4	5	6	7	Most

SENSITIVE: Equine can understand or read the mood, disposition, feelings, or intentions of other horses often on the basis of subtle, minimal cues.

Least	1	2	3	4	5	6	7	Most

SOLITARY: Equine prefers to spend considerable time alone not seeking or avoiding contact with other horses.

Least	1	2	3	4	5	6	7	Most

UNPERCEPTIVE: Equine is slow to respond or understand moods, dispositions, or behaviours of others.

Least	1	2	3	4	5	6	7	Most

DEPRESSED: Equine does not seek out social interactions with others and often fails to respond to social interactions of other horses. Equine often appears isolated, withdrawn and has reduced activity.

Least	1	2	3	4	5	6	7	Most

UNEMOTIONAL: Equine is relatively placid and unlikely to become aroused, upset, happy, or sad.

Least	1	2	3	4	5	6	7	Most

COOL: Equine seems unaffected by emotions and is usually undisturbed, assured, and calm.

Least	1	2	3	4	5	6	7	Most

PREDICTABLE: Equine's behaviour is consistent and steady over extended periods of time. Equine does little that is unexpected or deviates from its usual behavioural routine.

Least	1	2	3	4	5	6	7	Most

CONVENTIONAL: Equine seems to lack spontaneity or originality. Equine behaves in a consistent manner from day to day and stays well within the social rules of the group.

Least	1	2	3	4	5	6	7	Most

ASSERTIVE: Equine is able to displace, threaten, or take resources from other horses. Or equine may express high status by decisively intervening in social interactions.

Least	1	2	3	4	5	6	7	Most

INDIVIDUALISTIC: Equine's behaviour stands out compared to that of the other individuals in the group. This does not mean that it does not fit or is incompatible with the group.

Least	1	2	3	4	5	6	7	Most

PERSISTENT: Equine tends to continue in a course of action, task, or strategy for a long time or continues despite opposition from other horses or humans.

Least	1	2	3	4	5	6	7	Most

DECISIVE: Equine is deliberate, determined, and purposeful in its activities.

Least	1	2	3	4	5	6	7	Most

INDEPENDENT: Equine is individualistic and determines its own course of action without control or interference from other horses.

Least	1	2	3	4	5	6	7	Most

SOCIABLE: Equine seeks and enjoys the company of other horses and engages in amicable, affable, interactions with them.

Least	1	2	3	4	5	6	7	Most

SYMPATHETIC: Equine seems to be considerate and kind towards others as if sharing their feelings or trying to provide reassurance.

Least	1	2	3	4	5	6	7	Most

HELPFUL: Equine is willing to assist, accommodate, or cooperate with other horses.

Least	1	2	3	4	5	6	7	Most

GENTLE: Equine responds to others in an easy-going, kind, and considerate manner. Equine is not rough or threatening.

Least	1	2	3	4	5	6	7	Most

AFFECTIONATE: Equine seems to have a warm attachment or closeness with other horses or humans. This may entail frequently grooming, touching, or spending time next to others.

Least	1	2	3	4	5	6	7	Most

FRIENDLY: Equine often seeks out contact with other horses for amiable, genial activities. Equine infrequently initiates hostile behaviours towards other horses.

Least	1	2	3	4	5	6	7	Most

BULLYING: Equine is overbearing and intimidating towards younger or more acquiescent horses.

Least	1	2	3	4	5	6	7	Most

CONFRONTATIONAL: Equine often initiates fights or other agonistic encounters with other horses.

Least	1	2	3	4	5	6	7	Most

IRRITABLE: Equine often seems in a bad mood or is impatient and easily provoked to anger, exasperation, and consequent agonistic behaviour.

Least	1	2	3	4	5	6	7	Most

DEFIANT: Equine is assertive or contentious in a way inconsistent with the usual dominance order. Equine maintains these actions despite unfavourable consequences or threats from others.

Least	1	2	3	4	5	6	7	Most

CURIOUS: Equine has a desire to see or know about objects, people, or other horses. This includes a desire to know about the affairs of other horses or activities that do not directly concern the equine.

Least	1	2	3	4	5	6	7	Most

INNOVATIVE: Equine engages in new or different behaviours that may involve the use of objects or materials or ways of interacting with others.

Least	1	2	3	4	5	6	7	Most

INVENTIVE: Equine is more likely than others to do new things including novel social or non-social behaviours. Novel behaviour may also include innovative ways of using objects or materials.

Least	1	2	3	4	5	6	7	Most

INTELLIGENT: Equine is quick and accurate in judging and comprehending both social and non-social situations. Equine is perceptive and discerning about social relationships.

Least	1	2	3	4	5	6	7	Most

S6 Preference testing

Food: At least 24 hours prior to the trial day (but not more than 72 hours prior) each subject undertook a food preference test to ensure that a high value food reinforcer was selected for their food trial. The method was similar to that of Vinassa, Cavallini [30] was followed whereby a three-choice repeated measures preference test was set up. A negative control (0.5kg of the horse's usual hay or haylage) was placed in one red bucket (previously left with the owner of the subject for habituation through provision of food in the bucket on two occasions prior to the preference test) two additional trial feedstuffs (500 grams of Baileys Horse Fibre Plus Nuggets and one Horslyx Mini Original equine lick tub) were placed separately in two other identical red buckets [31]. The buckets were placed 0.5m apart opposite the entrance, in a test space (usually the horses stable) and the equine released to approach the buckets at will. The feedstuff approached first, time spent eating each foodstuff and eaten weight after a two-minute period were recorded for each horse. This method was repeated twice more with the presentation of buckets in a randomised location order at each trial presentation to reduce order or location effect. The feedstuff that the horse spent longest eating and ate most of on each trial was recorded as the preferred substrate and the modal feedstuff recorded across the trials was selected as the feedstuff to be presented during the study.

Wither scratching: Prior to the trial day each horse was tested on three occasions for a two-minute period to identify their responses to different style (fast or slow scratching), and location of wither scratching. Preferences were recorded for each trial and the modal preference was selected for the study. Behaviours such as leaning into the scratches, head and neck extension, changes in mouth position (Figure 1) and attempts to groom the human scratching them or to groom inanimate objects were identified as positive indicators of enjoyment of the scratching style [32]. Avoidance behaviours [28] were identified as indication of a negative response to the scratching style. Scratching style preferred was then utilised on the individual equine during their trial.

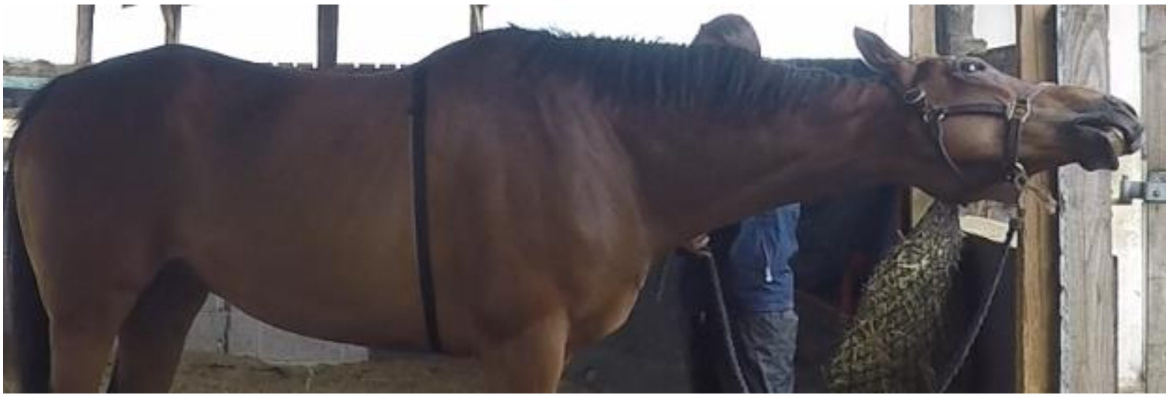

a

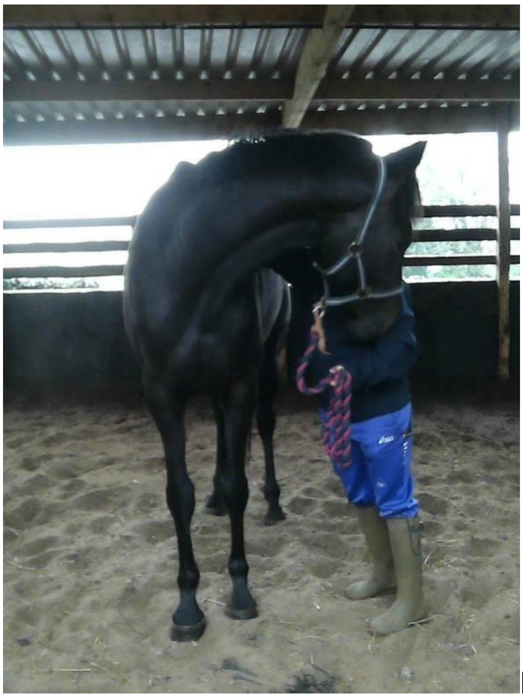

b

Figure 1 Examples of positive responses to wither scratching a) head and neck extension with mouth position changes and b) grooming of the human scratcher during wither scratching.

Preference test results for each subject are presented in Table 1 below.

Table 1 Preference test results for wither scratching area and food preference. Results for each of the three tests per induction method are presented.

LF = Left-hand side fast, LS = left-hand side slow, RF = right-hand side fast, RS = right-hand side slow, TF = top of wither fast, TS = top of wither slow. H = Hay, N = Nuggets, L = Lick.

**Equine number**	**Wither preference test result**			**Method selected**	**Food preference test result**			**Method selected**
	**Test 1**	**Test 2**	**Test 3**		**Test 1**	**Test 2**	**Test 3**	
1	LF	LF	LF	LF	N	N	N	N
2	TS	TS	TF	TS	L	N	N	N
3	LF	LF	LF	LF	N	N	N	N
4	RS	RS	TS	RS	L	L	L	L
5	LS	LS	LS	LS	L	N	N	N
6	RF	RF	RF	RF	N	N	N	N
7	LS	LS	LS	LS	L	N	N	N
8	LF	LF	LF	LF	N	N	N	N
9	LF	LF	LF	LF	L	L	L	L
10	TS	TS	TF	TS	N	N	N	N
11	RS	RS	RS	RS	N	L	N	N
12	LS	LS	LS	LS	N	N	N	N
13	LS	LS	LS	LS	N	N	N	N
14	TS	TS	TS	TS	L	L	L	L
15	RS	RS	RS	RS	N	N	N	N
16	LF	LF	LF	LF	N	N	N	N
17	LF	LF	LF	LF	N	N	N	N
18	LF	LF	LF	LF	N	N	N	N
19	LS	LS	LS	LS	L	N	N	N
20	TS	TS	TS	TS	N	N	N	N

S7 Induction trial randomised allocation

Horse number	Trial 1	Trial 2	Trial 3	Trial 4
1	Training	Wither scratch	Companion	Food
2	Food	Companion	Wither scratch	Training
3	Wither scratch	Companion	Training	Food
4	Food	Companion	Wither scratch	Training
5	Food	Wither scratch	Companion	Training
6	Wither scratch	Food	Companion	Training
7	Food	Companion	Training	Wither scratch
8	Training	Food	Wither scratch	Companion
9	Food	Wither scratch	Companion	Training
10	Food	Companion	Training	Wither scratch
11	Wither scratch	Food	Training	Companion
12	Wither scratch	Training	Companion	Food
13	Wither scratch	Companion	Food	Training
14	Companion	Wither scratch	Training	Food
15	Training	Food	Companion	Wither scratch
16	Wither scratch	Companion	Food	Training
17	Food	Training	Companion	Wither scratch
18	Training	Food	Wither scratch	Companion
19	Companion	Food	Wither scratch	Training
20	Companion	Food	Training	Wither scratch

S8 The ear and eye positions with descriptors and images as utilised by the video scorers. Ethogram developed from prior published ethograms[9].

Ears forwards	Ears sideways	Ear asymmetrical	Ear gently back	Ear back	Open	Half	Sclera
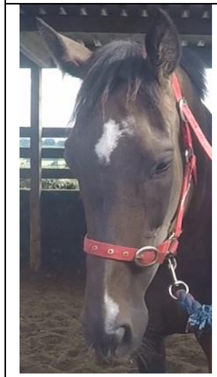	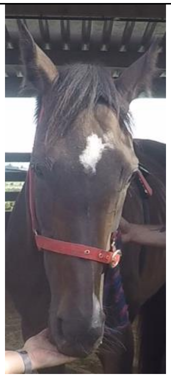	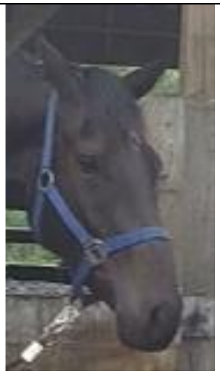	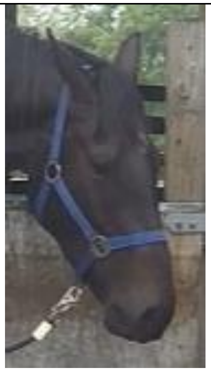	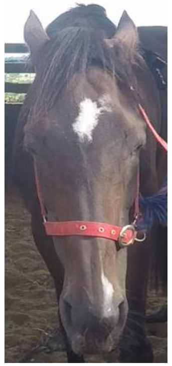	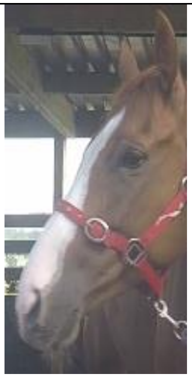	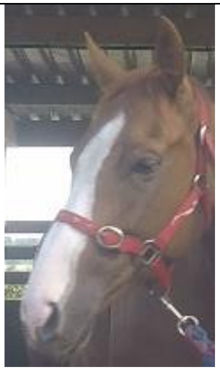	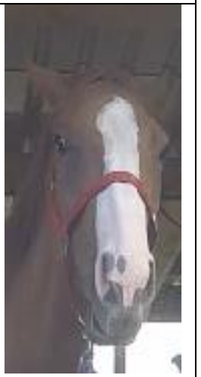
Both ears facing forwards. The inner ear can be seen.	Boths ears facing to the side and relaxed.	Each ear facing in a different direction, may be forward, sideways or backwards.	Both ears rotated backwards but are relaxed, inner ear not seen.	Both ears rotated fully backwards and flattened.	The eye is fully open with no sclera (eye white) showing.	The eye is half closed with the upper eyelid partially covering the lower eye.	Some white of the eye is showing.

Eye bias positions with images and descriptors. Ethogram developed from Des Roches, Richard-Yris [2] and Austin and Rogers [33].

Both	Left	Right
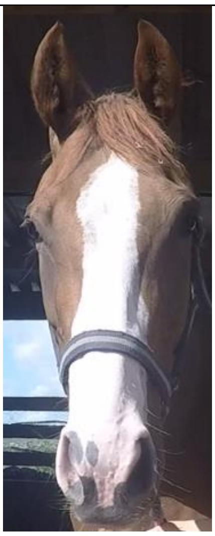	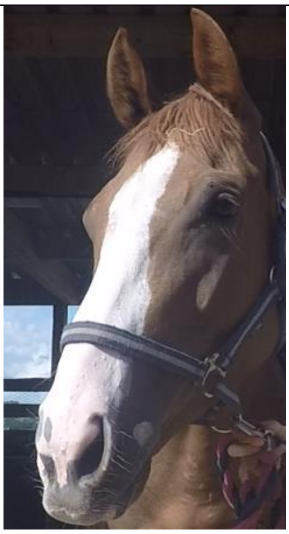	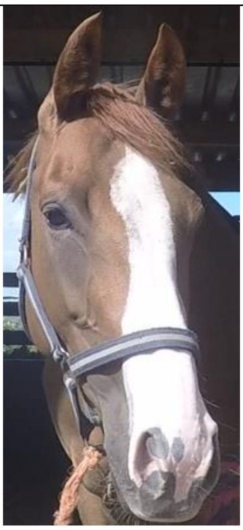
The horse is focussing on the stimulus with both eyes equally or is looking straight ahead.	The horse angles or turn the head to focus more with the left eye than the right eye.	The horse angles or turn the head to focus more with the right eye than the left eye.

S9 QBA names and descriptors from AWIN [13]

Indicator name	Descriptor
Aggressive	Hostile, attacking, wants to fight/attack, dominance, defensive aggression, (i.e. may display the following: bite/kick, position of ears flat-back against head, dilated nostrils, turns the hindquarters towards object of aggression, intention to harm, tail-swishing)
Alarmed	Worried/tense, apprehensive, jumpy, nervous, watchful, on guard against a possible threat/danger (i.e. rigid stance, startled reaction to loud noise, looking around/vigilant, moving ears)
Annoyed	Irritated, displeased, bothered by something, disturbed, upset, troubled, exasperated (i.e. may display rapid tail-swishing, stomping)
Apathetic	Having or showing little or no emotion; disinterested, indifferent, isolated, depressed, unresponsive, motionless
At ease	Calm, carefree, peaceful
Curious	Inquisitive, desire to investigate (i.e. approach person/object of curiosity, engaged in exploratory behaviour; possibly displaying

	head and neck extended toward object of curiosity, with ears pricked forward)
Friendly	Affectionate, kind, not hostile, receptive, positive feelings toward people, confident (i.e. the horse approaches the person, may sniff or interact in some way)
Fearful	Afraid, hesitant, timid, not confident, not necessarily linked with something going on in the environment (i.e. you may see the body tremble, flared nostrils, tail clamped)
Happy	Feeling, showing or expressing joy, pleased, lively, playful, satisfied
Look for contact	Actively looking for interaction, interested, close proximity, eager to approach
Relaxed	Not tense or rigid, easy-going, tranquil
Pushy	Assertive or forceful (i.e. not leaving space, head butting out of the way, exhibits dominant behaviour, may be mouthy or nippy)
Uneasy	Afflicted, uncomfortable, unsettled, restless

S10 Indicators of frustration from Pannewitz and Loftus [3]

Indicator name	Descriptor
Acute - conflict	The horse shows some resistance to the stimulus or a person including avoiding interaction, trying to move away, being hesitant
Acute - Displacement	The horse engages in another behaviour such as bed eating, increased eating or drinking, active looking away, mouthing items or people, undertaking crib-biting, wind sucking, weaving or repetitive pacing) rather than engaging with the stimulus
Acute - vocalisation	The horse produces a sound
Acute – redirected aggression	The horse is aggressive to something that is not the stimulus being presented
Acute – direct aggression	The horse is aggressive to the stimulus being presented
Acute – increased locomotion	The horse moved around (this may be anything from one step to an entire movement sequence
Acute – muscle tension	The horse becomes tense in its posture

Chronic – repetitive behaviours	The horse engages in repetitive behaviours for a prolonged period of time
Chronic – learned helplessness	The horse is unresponsive and inappetent for a prolonged period of time
Chronic – altered cognitive bias	The horse shows a prolonged change in judgement about the stimulus

S11 Salivary cortisol lateral flow device testing process

Step one

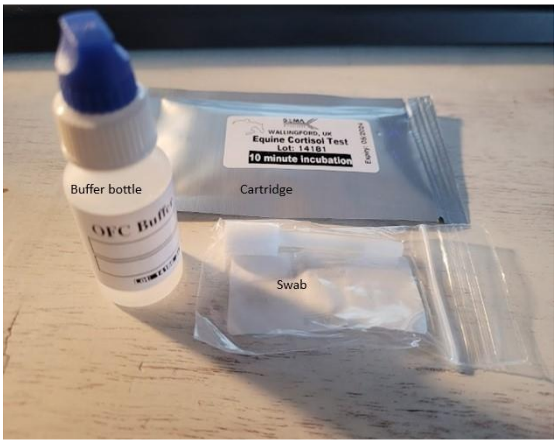

One oral swab, LFD cartridge and oral fluid collector (OFC) bottle with buffer fluid were used per phase of each trial in each horse.

Step two

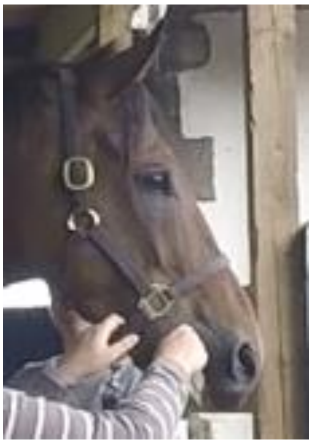

The saliva sample is collected using the swab inserted in the diastema between the incisor and molar teeth and maintained in position until the blue indicator line shows on the stem of the swab.

Step three

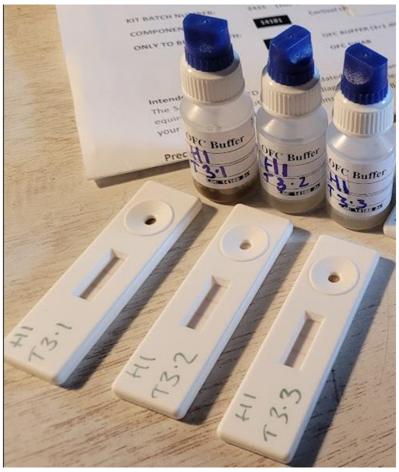

The swab is placed in an OFC bottle labelled with the horse number, trial number (T1-T4) and trial phase (.1 = before, .2 = immediately after, .3 = 15 minutes after) and gently inverted for 1-2 minutes. The corresponding LFD cartridge is labelled in the same manner as the bottle.

Step four

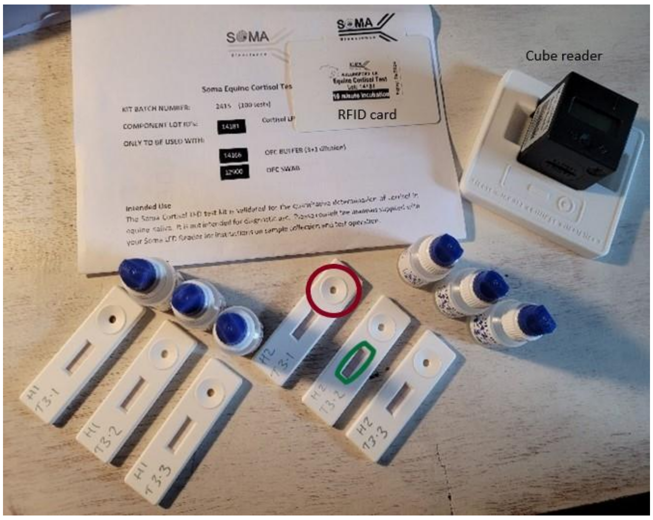

After a 10-minute incubation period two drops of the saliva-buffer mix are dropped onto the sample window (circled red) of the LFD cartridge and when the test window (green rectangle) turns red in colour the test is ready to run. The cube reader is turned on and the RFID calibration card is placed on the cube reader to verify the test. The test is run by pressing the run button on the top of the cube reader and the result appears on the cube screen within 4 seconds. Results were recorded in an excel sheet.

S12 Eye and ear thermography measurements

Data reading positions for the left eye (Sp1), right eye (Sp2), left ear (Sp3) and right ear (Sp4). A marker line indicated the slope across the base of each ear to aid data spot recording accuracy as per the method of Kappel[34]. Eye temperature was taken from the medial canthus of the eye as suggested by Kim and Cho [35].

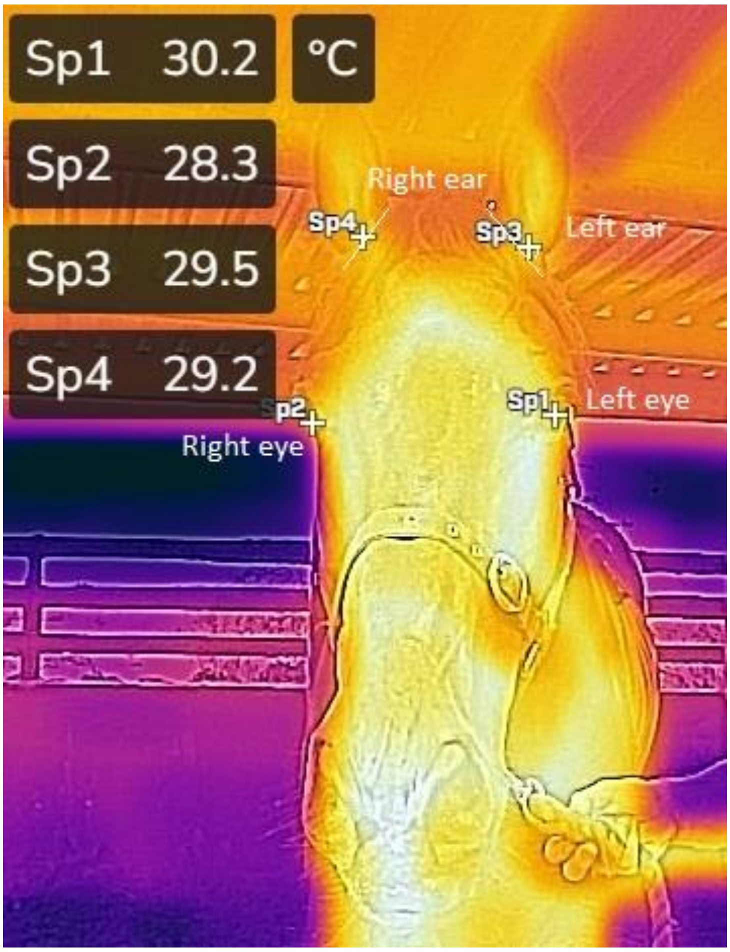

S13 Video and physiological data storage and handling

Following each day of data collection video data were uploaded to secure cloud storage and each file named with the subject number and trial number (blinded to induction method treatment) as well as the date and time of the trial session, they were stored in folders names ‘front, ‘back’ and ‘side’ according to the camera angle they were taken from. Thermography data were uploaded to FLIR Ignite [36] secure cloud storage after each day of data collection and the files named in the same method as for video files. Cortisol buffer bottles were labelled at the time of collection with the horse number and trial number and the swab immediately placed in the buffer bottle and sealed once saliva collection was complete. The saliva samples were analysed via Lateral Flow Device (LFD) on the day of collection and then sent in batches for laboratory Elisa analysis. Heart rate and heart rate variability data were uploaded to Polar Flow software [37] at the end of each day of data collection with each dataset labelled with the subject number and trial number and the date and time automatically attributed via the Polar software.

Behavioural data were scored by the principal researcher and five further scorers using CowLog software with predetermined items (figure below).

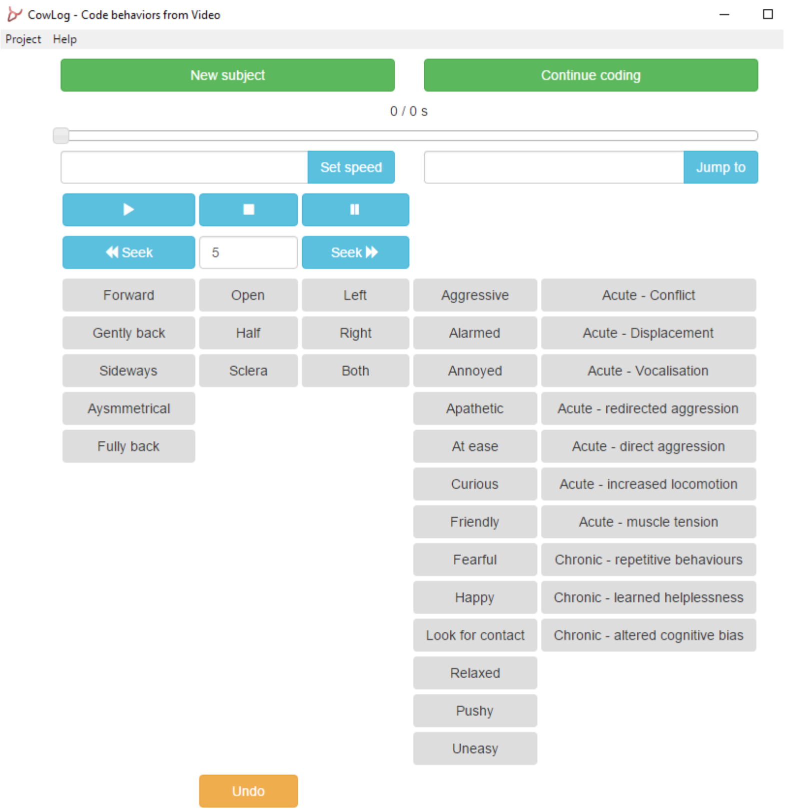

S14 Interval plots

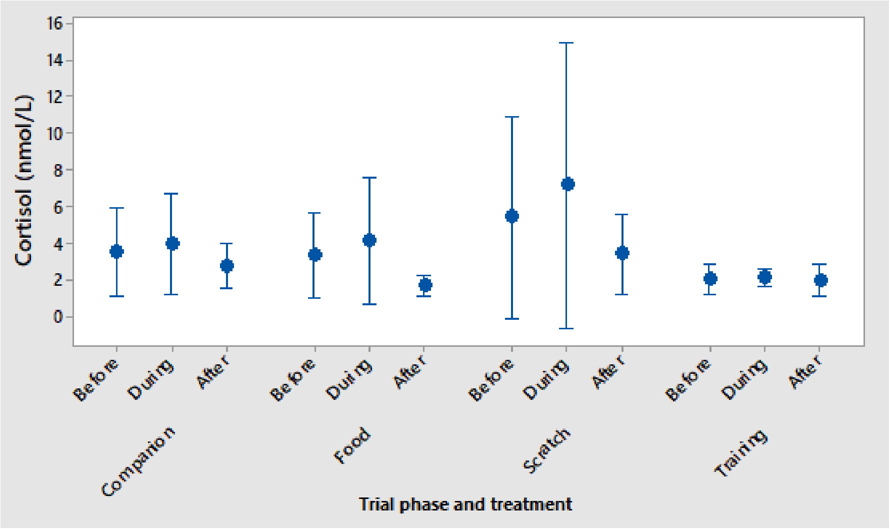

Interval plot of cortisol data by trial phase and treatment with confidence intervals.

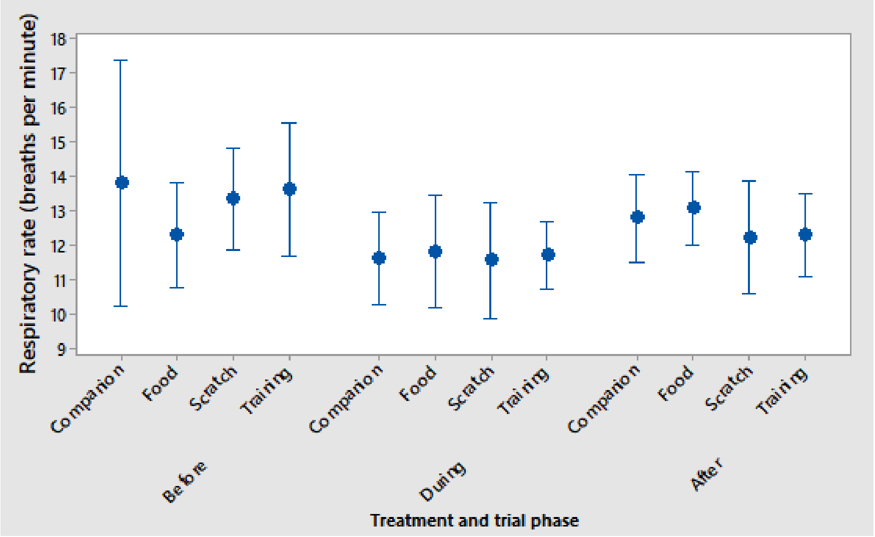

Interval plot of respiratory data by trial phase and treatment with confidence intervals.

References

1. Tomberg, C., M. Petagna, and L.-A. de Selliers de Moranville, *Horses (Equus caballus) facial micro-expressions: insight into discreet social information.* Scientific Reports, 2023. **13**(1): p. 8625.
2. Des Roches, A.D.B., et al., *Laterality and emotions: Visual laterality in the domestic horse (Equus caballus) differs with objects' emotional value.* Physiology & Behavior, 2008. **94**(3): p. 487-490.
3. Pannewitz, L. and L. Loftus, *Frustration in horses: Investigating expert opinion on behavioural indicators and causes using a delphi consultation.* Applied Animal Behaviour Science, 2023. **258**: p. 105818.
4. Loftus, L., et al., *Monty Roberts' public demonstrations: Preliminary report on the heart rate and heart rate variability of horses undergoing training during live audience events.* Animals, 2016. **6**(9): p. 55.
5. Scopa, C., et al., *Inside the Interaction: Contact With Familiar Humans Modulates Heart Rate Variability in Horses.* Frontiers in Veterinary Science, 2020. **7**.
6. Bornmann, T., H. Randle, and J. Williams, *Investigating equestrians' perceptions of horse happiness: An exploratory study.* Journal of Equine Veterinary Science, 2021. **104**: p. 103697.
7. Ricci-Bonot, C. and D.S. Mills, *Recognising the facial expression of frustration in the horse during feeding period.* Applied Animal Behaviour Science, 2023. **265**: p. 105966.
8. Wathan, J., et al., *EquiFACS: The equine facial action coding system.* PLoS one, 2015. **10**(8): p. e0131738.
9. Lansade, L., et al., *Facial expression and oxytocin as possible markers of positive emotions in horses. .* Scientific reports, 2018. **8**(11): p. 1-11.
10. Hintze, S. and L. Schanz, *Using the Judgment Bias Task to Identify Behavioral Indicators of Affective State: Do Eye Wrinkles in Horses Reflect Mood?* Front Vet Sci, 2021. **8**: p. 676888.
11. Leliveld, L.M., J. Langbein, and B. Puppe, *The emergence of emotional lateralization: evidence in non-human vertebrates and implications for farm animals.* Applied Animal Behaviour Science, 2013. **145**(1-2): p. 1-14.
12. Evans, L., H. Cameron-Whytock, and C. Ijichi, *Eye understand: Physiological measures as novel predictors of adaptive learning in horses.* Applied Animal Behaviour Science, 2024. **271**: p. 106152.
13. AWIN, *AWIN Welfare assessment protocol for horses*, M. M., E. Dalla Costa, and F. Dai, Editors. 2015.
14. Dalla Costa, E., et al., *Welfare assessment of horses: the AWIN approach.* Animal Welfare, 2016. **25**(4): p. 481-488.
15. Ruet, A., et al., *Horse welfare: A joint assessment of four categories of behavioural indicators using the AWIN protocol, scan sampling and surveys.* Animal Welfare, 2022. **31**(4): p. 455-466.
16. Minero, M., et al., *Using qualitative behaviour assessment (QBA) to explore the emotional state of horses and its association with human-animal relationship.* Applied Animal Behaviour Science, 2018. **204**: p. 53-59.
17. Hall, C., et al., *Assessing equine emotional state.* Applied Animal Behaviour Science, 2018. **205**: p. 183-193.

18. Pagani, M., et al., *Power spectral density of heart rate variability as an index of sympatho-vagal interaction in normal and hypertensive subjects.* Journal of hypertension. Supplement: official journal of the International Society of Hypertension, 1984. **2**(3): p. S383-5.
19. Stucke, D., M.G. Ruse, and D. Lebelt, *Measuring heart rate variability in horses to investigate the autonomic nervous system activity–Pros and cons of different methods.* Applied animal behaviour science, 2015. **166**: p. 1-10.
20. Pham, T., et al., *Heart Rate Variability in Psychology: A Review of HRV Indices and an Analysis Tutorial.* Sensors, 2021. **21**(12): p. 3998.
21. Shaffer, F. and J.P. Ginsberg, *An Overview of Heart Rate Variability Metrics and Norms.* Front Public Health, 2017. **5**: p. 258.
22. Billman, G.E., *The LF/HF ratio does not accurately measure cardiac sympatho-vagal balance.* Front Physiol, 2013. **4**: p. 26.
23. Mormède, P., et al., *Breeding for robustness: the role of cortisol.* animal, 2011. **5**(5): p. 651-657.
24. van Ast, V.A., et al., *Modulatory mechanisms of cortisol effects on emotional learning and memory: Novel perspectives.* Psychoneuroendocrinology, 2013. **38**(9): p. 1874-1882.
25. Otzenberger, H., et al., *Dynamic heart rate variability: a tool for exploring sympathovagal balance continuously during sleep in men.* American Journal of Physiology-Heart and Circulatory Physiology, 1998. **275**(3): p. H946-H950.
26. Ciccone, A.B., et al., *Reminder: RMSSD and SD1 are identical heart rate variability metrics.* Muscle & nerve, 2017. **56**(4): p. 674-678.
27. Maurício, L.S., D.P. Leme, and M.J. Hötzel, *How to Understand Them? A Review of Emotional Indicators in Horses.* Journal of Equine Veterinary Science, 2023. **126**: p. 104249.
28. Draaisma, R., *Language signs and calming signals of horses: recognition and application*. 2017, New york: CRC Press.
29. Lansade, L., et al., *Automatic brushes induce positive emotions and foster positive social interactions in group-housed horses.* Applied animal behaviour science, 2022. **246**: p. pp. 105538.
30. Vinassa, M., et al., *Palatability assessment in horses in relation to lateralization and temperament.* Applied Animal Behaviour Science, 2020. **232**: p. 105110.
31. RedGorilla. *Gorilla Tub Mini Shallow 5L*. 2024; Available from: https://www.redgorilla.red/products/gorilla-tub-mini-5l?variant=47370075570486.
32. McGreevy, P.D., et al., *The horse-human dyad: can we align horse training and handling activities with the equid social ethogram?* The Veterinary Journal, 2009. **181**(1): p. 12-8.
33. Austin, N.P. and L.J. Rogers, *Asymmetry of flight and escape turning responses in horses.* Laterality, 2007. **12**(5): p. 464-474.
34. Kappel, S., *Towards the Validation of Novel Cognitive and Physiological Indicators of Affective State in Horses (Equus caballus)*. 2023, University of Plymouth.
35. Kim, S.-M. and G.-J. Cho, *Validation of Eye Temperature Assessed Using Infrared Thermography as an Indicator of Welfare in Horses.* Applied Sciences, 2021. **11**(16): p. 7186.
36. TeledyneFlir, *FLIR Ignite*. 2024, Teledyne Flir.
37. PolarElectro, *Polar Flow*. 2024.
